# Evolution of an adenine base editor into a small, efficient cytosine base editor with low off-target activity

**DOI:** 10.1038/s41587-022-01533-6

**Published:** 2022-11-10

**Authors:** Monica E. Neugebauer, Alvin Hsu, Mandana Arbab, Nicholas A. Krasnow, Amber N. McElroy, Smriti Pandey, Jordan L. Doman, Tony P. Huang, Aditya Raguram, Samagya Banskota, Gregory A. Newby, Jakub Tolar, Mark J. Osborn, David R. Liu

**Affiliations:** 1 Merkin Institute of Transformative Technologies in Healthcare, Broad Institute of MIT and Harvard, Cambridge, MA USA; 2 Department of Chemistry and Chemical Biology, Harvard University, Cambridge, MA USA; 3 Howard Hughes Medical Institute, Harvard University, Cambridge, MA USA; 4 Department of Pediatrics, University of Minnesota Medical School, Minneapolis, MN USA

**Keywords:** Genetic engineering, Targeted gene repair

## Abstract

Cytosine base editors (CBEs) are larger and can suffer from higher off-target activity or lower on-target editing efficiency than current adenine base editors (ABEs). To develop a CBE that retains the small size, low off-target activity and high on-target activity of current ABEs, we evolved the highly active deoxyadenosine deaminase TadA-8e to perform cytidine deamination using phage-assisted continuous evolution. Evolved TadA cytidine deaminases contain mutations at DNA-binding residues that alter enzyme selectivity to strongly favor deoxycytidine over deoxyadenosine deamination. Compared to commonly used CBEs, TadA-derived cytosine base editors (TadCBEs) offer similar or higher on-target activity, smaller size and substantially lower Cas-independent DNA and RNA off-target editing activity. We also identified a TadA dual base editor (TadDE) that performs equally efficient cytosine and adenine base editing. TadCBEs support single or multiplexed base editing at therapeutically relevant genomic loci in primary human T cells and primary human hematopoietic stem and progenitor cells. TadCBEs expand the utility of CBEs for precision gene editing.

## Abstract (web summary)

Improved cytosine base editors are generated by phage-assisted evolution of a deoxyadenosine deaminase.

## Main

Base editors consist of a programmable DNA-binding protein fused to a deaminase and enable precise nucleotide changes at targeted genomic loci without requiring a double-stranded break^1–4^. Current CBEs, which convert C•G base pairs to T•A, consist of cytidine deaminases fused to a Cas9 nickase or a TALE repeat array and uracil glycosylase inhibitor (UGI) domains^1,5^. All CBEs published to date harness naturally occurring cytidine deaminases that operate on DNA or laboratory-engineered variants thereof. ABEs convert A•T base pairs to G•C. Because no natural enzyme is known to deaminate deoxyadenosine, we previously evolved an adenosine deaminase that acts on transfer RNA (tRNA) to accept DNA substrates, resulting in the deoxyadenosine deaminase TadA-7.10 (refs. ^2,6,7^). All reported ABEs to date^2,6–8^, including those already in clinical trials^9^ or cleared for clinical trials^10^, use TadA-7.10 or evolved or engineered descendants of this deaminase.

ABEs exhibit many properties desirable for precision gene editing. Current-generation ABE variants, such as ABE8e, typically achieve higher editing efficiencies than existing CBEs, despite the strong tRNA substrate preference of wild-type TadA^7,11,12^. Compared to most CBE deaminases, TadA enzymes are less processive and, therefore, typically enable greater single-nucleotide editing precision^1,5,6,11^. ABEs also offer lower levels of Cas-independent off-target editing compared to CBEs^6,7,13–15^. This advantage likely arises from tighter unassisted binding of commonly used cytidine deaminases to nucleic acid substrates (for example, APOBEC1 K_m_ = 0.21 nM for mRNA^16^) compared to that of wild-type TadA (K_m_ = 830 nM for a tRNA stem^17^), the inability of wild-type TadA to process DNA and the fact that we evolved TadA-7.10 solely in a Cas-dependent manner^2,6,7,18^. Genome mining^19^ and protein engineering have provided alternative cytidine deaminases with lower Cas-independent DNA and RNA editing, but, to date, these variants suffer from reduced on-target editing activity and/or larger size^15,20–24^.

At 166 amino acids, evolved TadA deoxyadenosine deaminases are substantially smaller than commonly used cytidine deaminases such as APOBEC1 (227 amino acids), AID (182 amino acids)^25^, CDA (207 amino acids)^5^ or A3A (198 amino acids)^26^, making TadA-derived base editors easier to deliver into cells by size-constrained methods, such as adeno-associated virus (AAV). Indeed, the small size of TadA has enabled ABEs, but not CBEs, to be delivered into animal tissues in vivo using a single AAV^27,28^.

We envisioned that the directed evolution of a TadA-derived deoxyadenosine deaminase to perform deoxycytidine deamination might yield CBEs that maintain high on-target activity but inherit the lower Cas-independent off-target editing and smaller size of current ABEs. Wild-type TadA is evolutionarily related to cytidine deaminases^29,30^, raising the possibility that laboratory evolution could traverse a fitness landscape to enable cytidine deamination. Indeed, low levels of cytidine deamination have been reported in evolved ABE variants^11,31,32^. Further mutagenesis of TadA-7.10 (TadA-7.10 P48R) was shown to disrupt deoxyadenosine selectivity and increase cytidine deamination in 5′-TC contexts at protospacer position 6 in the editing window (counting the SpCas9 PAM as positions 21–23)^32^, although adenosine deamination is still preferred at other contexts and positions. In addition, adenosine deaminases acting on RNA (ADARs) have been evolved to perform both cytidine and adenosine deamination in RNA^33^.

**Fig. 1:**
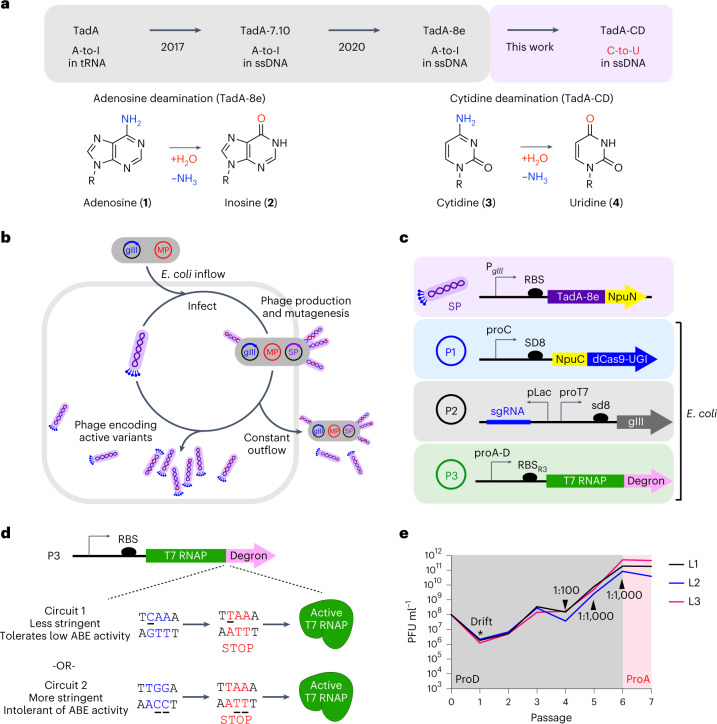
Phage-assisted evolution of a cytidine deaminase from TadA-8e. **a**, Evolutionary trajectory of a TadA-based cytidine deaminase from the tRNA deaminase, TadA. **b**, PACE overview. The selection phage (purple) encodes the evolving protein. *E. coli* hosts (gray) contain (1) a mutagenesis plasmid to diversify the phage (red) and (2) a plasmid system that regulates the expression of pIII (blue, encoded by gIII). Only variants with the desired activity trigger production of pIII and propagate. Phage without the desired activity cannot propagate and are diluted out of the lagoon. **c**, Selection circuit for cytidine deamination. TadA-8e variants are encoded on the SP (purple). The *E. coli* harbor three plasmids that establish the selection circuit, in addition to the mutagenesis plasmid: P1 contains the Cas9-UGI components of the base editor. Upon phage infection, the full base editor is reconstituted though the split Npu intein system (yellow). P2 encodes the guide RNA and gIII, which is under transcriptional control of the T7 promoter. P3 contains T7 RNA polymerase that is inactivated by fusion to a degron tag. C•G-to-T•A editing activity inserts a stop codon between T7 RNAP and the degron to yield active T7 RNAP, which leads to transcription of gIII and phage propagation. **d**, Two versions of the CBE circuit used in this work. In both cases, C•G-to-T•A editing inserts a stop codon before the degron tag, leading to active T7 RNAP. The less stringent circuit requires a C•G-to-T•A edit on the coding strand (top) and can tolerate one undesired A-to-G edit. The more stringent circuit requires a C•G-to-T•A edit on the non-coding, transcription template strand and cannot tolerate any undesired A•T-to-G•C edit. **e**, PANCE of a deoxycytidine deaminase from TadA-8e. The ProD (stronger, less stringent) or ProA (weaker, more stringent) promoter used in each PANCE passage is shown. At each passage, phage are diluted 1:50 unless indicated otherwise. After several rounds of evolution, phage titers stabilize despite increasing dilution rates between passages, suggesting the evolution of deoxycytidine deamination activity. ssDNA, single-stranded DNA. Source data

In this study, we used phage-assisted continuous and non-continuous evolution (PACE and PANCE) to change the substrate specificity of TadA-8e, resulting in a new class of selective cytidine deaminases (TadA-CDs) and CBEs (Fig. 1a). To enable cytidine deamination, TadA-CD variants acquired mutations at residues that interact with the DNA backbone near the active site. TadA-CD cytosine base editors (TadCBEs) are highly active and exhibit similar or higher C•G-to-T•A editing efficiencies compared to current BE4max, evoAPOBEC1-BE4max (evoA) and evoFERNY-BE4max (evoFERNY) CBEs across a variety of sites in mammalian cells. Off-target analysis reveals that TadCBEs induce lower Cas-independent off-target DNA and RNA editing than widely used APOBEC-based CBE variants. The addition of a V106W mutation^7,34^ further reduces off-target editing by TadCBEs, refines their editing window and improves C•G-to-T•A selectivity while preserving peak on-target editing efficiency. We extensively characterized evolved TadCBEs using a library of 10,638 genomically integrated, highly variable target sites in mouse embryonic stem cells (mESCs) to determine the selectivity and sequence context preferences of TadCBEs. TadA-CDs are also compatible with SpCas9 (PAM = NGG), evolved eNme2-C Cas9 (PAM = N_4_CN) variants and SaCas9 (PAM = NNGRRT), facilitating broad target accessibility. Finally, we demonstrate that TadCBEs can be used for efficient multiplexed cytosine base editing in primary human T cells at therapeutically relevant loci and for cytosine base editing at a therapeutically relevant site in primary human hematopoietic stem and progenitor cells (HSPCs). Taken together, these findings reveal a new family of small CBEs with high on-target activity, well-defined editing windows that facilitate precise base editing and low off-target activity. Our findings also establish the potential of deoxyadenosine deaminases to evolve into selective deoxycytidine deaminases.

## Results

### Design of a selection for deoxycytidine deamination

PACE has enabled the rapid laboratory evolution of diverse protein functions, including protein–protein interactions^35^, tRNA synthetases^36^, DNA-binding proteins^37–39^, proteases^40,41^, polymerases^42^, metabolic enzymes^43–45^ and base editors^7,12^. During PACE, the evolving protein is encoded on the selection phage (SP), which infect *Escherichia coli* host cells^46^. The *E. coli* harbor a mutagenesis plasmid (MP) that constantly mutagenizes the phage genome as well as accessory plasmid(s) (AP) that establish a selection circuit that regulates the expression of gene III, which encodes pIII, a critical protein for phage replication. Because gIII has been removed from the SP genome, only phage that encode evolving variants with the desired activity trigger the production of pIII in *E. coli* and replicate, resulting in the propagation of active gene variants (Fig. 1b). Under constant mutagenesis and dilution, phage lacking the desired activity are rapidly diluted from the selection vessel (‘lagoon’), whereas phage that evolve beneficial mutations persist.

Previously, we developed a CBE-PACE selection^12^ in which a cytidine deaminase is encoded within the SP, and host *E. coli* cells contain (1) the MP, (2) an accessory plasmid that encodes SpCas9, (3) a self-inactivating T7 RNA polymerase (T7 RNAP) fused to a C-terminal degron and (4) gene III under T7 RNAP transcriptional control. Upon phage infection, the SP-encoded deaminase is joined to Cas9 by trans-intein splicing to reconstitute the base editor. To activate the selection circuit, the base editor must perform C•G-to-T•A editing to create a stop codon between T7 RNAP and the degron, yielding active T7 RNAP. Degron-free T7 RNAP then transcribes gIII, leading to phage propagation^12^.

To develop a PACE circuit to select for cytidine deamination by TadA, we modified our previous CBE selection circuit to accommodate an enzyme with high initial deoxyadenosine deamination activity (Fig. 1c). In the original circuit, TGG (Trp) is edited into a stop codon (TAG, TGA or TAA) through C-to-T conversion of CCA in the template strand. This strategy, however, places adenine, which is opposite thymine in all stop codons (TAG, TGA and TAA), at position 6 within the target protospacer. Given that position 6 is highly edited by ABE8e^7^, and that A-to-G editing of A_6_ precludes stop codon formation because CGG, CAG, CGA and CAA all encode amino acids, this original circuit would require high selectivity for deoxycytidine over deoxyadenosine deamination that is unlikely to be found among early-stage evolved ABE8e variants.

To address this problem, we developed a new selection circuit that instead edits the non-template strand (Fig. 1d). In the new circuit, C_6_A_7_A_8_ is edited to T_6_A_7_A_8_ to introduce a stop codon upon deoxycytidine deamination. Deoxyadenosine deamination does not prevent stop codon installation (TAA, TGA or TAG) in the new selection unless both A_7_ and A_8_ are converted to Gs (TGG = Trp), making this circuit tolerant to modest levels of deoxyadenosine deamination and, thus, more suitable for early-stage TadA8e evolution (Circuit 1). After initial evolution in the new circuit, we envisioned switching to the original template-strand circuit (Circuit 2) to take advantage of its inherent strong negative selection against deoxyadenosine deamination (Supplementary Fig. 1).

### Deoxycytidine deaminase evolution

We initiated PANCE of TadA-8e using Circuit 1 (Fig. 1e). In PANCE, *E. coli* host cells containing the AP and MP are infected with phage containing the gene of interest and grown overnight, without continuous dilution. The next day, the supernatant containing the phage is diluted into a fresh host cell culture, and the process is repeated to enrich for phage harboring active cytidine deaminases. Compared to PACE, PANCE offers lower stringency and, thus, is helpful during early-phase evolution campaigns in which preserving genetically diverse variants with low initial activity can be critical^7,41,43^. After four rounds of PANCE with induced MP6 mutagenesis^47^, the phage began to propagate >100-fold overnight, suggesting improved activity for cytidine deamination. To increase the stringency of the selection, we increased the fold dilution between passages and decreased the strength of the promoter upstream of T7 RNAP (Supplementary Fig. 2). Next, we switched to Circuit 2 for additional passages of PANCE (Supplementary Fig. 2) to select against deoxyadenosine deamination while maintaining deoxycytidine deamination activity. To further increase selection stringency, we performed 159 hours of continuous evolution (PACE) on phage pools surviving PANCE using Circuit 2 (Supplementary Fig. 3). TadA-8e variants emerging from all phases of PANCE and PACE survived an average total dilution of ~10^139^-fold.

**Fig. 2:**
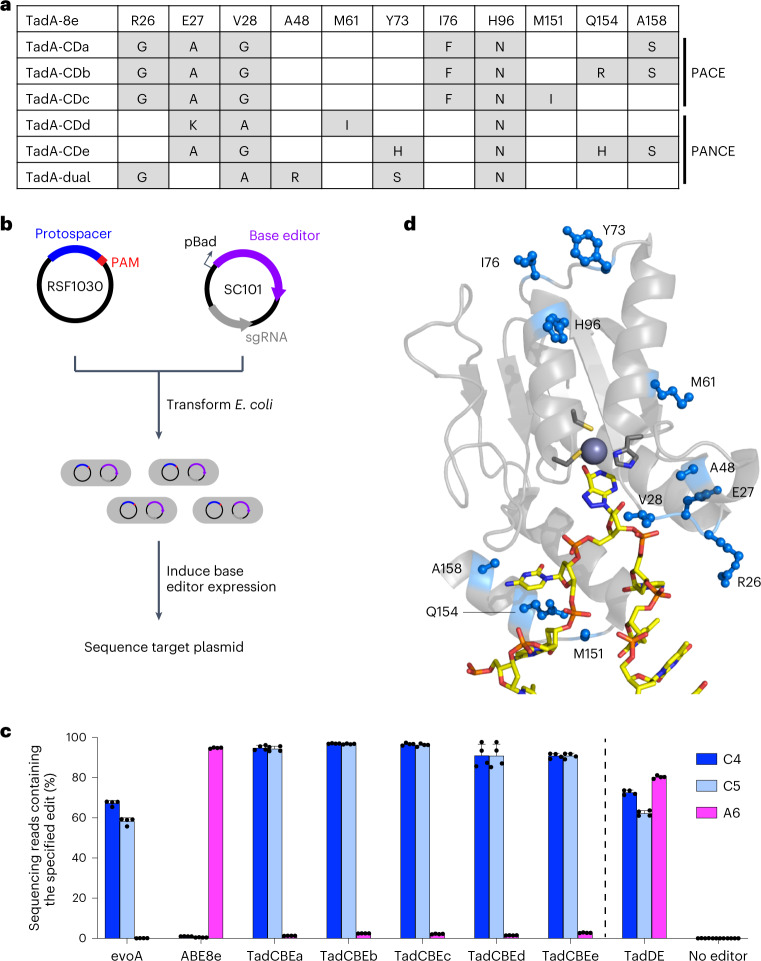
Evolved TadA* variants catalyze deoxycytidine deamination. **a**, Summary of TadA-8e variants evolved and characterized in this work. The variants are representative of conserved mutations after nine passages of PANCE or after 159 hours of PACE. For a full list of mutations, see Supplementary Figs. 2 and 3. **b**, Method for assessing base editing of target plasmids in *E. coli*. Cells are co-transformed with a target plasmid (blue) and a base editor plasmid (purple). Base editor expression is induced with arabinose. After 16 hours, cells are harvested, and the target plasmid is analyzed by high-throughput sequencing. **c**, Base editing in *E. coli* of a protospacer matching the selection circuit target site. C•G-to-T•A edits are shown in blue. A•T-to-G•C edits are shown in magenta. Dots represent individual biological replicates, and bars represent mean ± s.d. from four independent biological replicates. **d**, Locations of evolved mutations in the cryo-EM structure of ABE8e (PDB: 6VPC)^18^. Source data

We isolated and sequenced individual phage surviving PANCE and PACE to identify TadA-8e mutations acquired during evolution (Fig. 2a and Supplementary Figs. 2 and 3). We observed a striking prevalence of mutations in residues 26–28 across all the sequenced phages, with R26G, E27K, E27A and V28G mutations highly represented across several separately evolved lagoons. Next, we assayed the evolved variants for base editing in *E. coli*. We sub-cloned five evolved TadA variants (TadA-CDa–e) from phage into the BE4max architecture^48^ (from N-terminus to C-terminus: TadA*–SpCas9–UGI–UGI) on a low-copy plasmid and designed a high-copy target plasmid containing sequences from the selection circuits on which the phage evolved. We co-transformed the base editor plasmid, which also encodes the guide RNA, and the target plasmid into *E. coli* cells, allowed editing after arabinose induction to occur overnight, and performed high-throughput sequencing of the target plasmid (Fig. 2b).

The sequencing results revealed a striking shift in selectivity of the evolved TadA variants compared to the starting TadA-8e variant. Although base editors containing TadA-8e yielded 94% A•T-to-G•C editing at A_6_ and 1% C•G-to-T•A editing at C_4_ and C_5_ in the target plasmid, the evolved variants instead resulted in 90–97% editing of cytosines and 1–3% editing of adenine (Fig. 2c), representing a >3,000-fold change in cytosine versus adenine base editing. These results indicate that PANCE and PACE using selection Circuits 1 and 2 evolved TadA variants, hereafter referred to as TadA-cytidine deaminases (TadA-CDs), with strong cytidine deamination activity and high selectivity for cytosine over adenine base editing.

From a lagoon infected with TadA-8e A48R, containing a mutation that increases promiscuity in TadA-7.10 (ref. ^32^), we also identified a variant that performed both A•T-to-G•C (80%) and C•G-to-T•A (73%) editing in the *E. coli* editing assay (Fig. 2c). This variant thus serves as a TadA-based dual editor (TadDE). TadDE is smaller than previously reported dual editors that fuse both cytidine and adenosine deaminases to a Cas domain^49–53^ and may be especially useful for applications requiring broad mutagenesis^54^, such as genetic screens^55,56^.

To identify potential roles for the evolved mutations, we mapped them onto the cryogenic electron microscopy (cryo-EM) structure of ABE8e (Protein Data Bank (PDB): 6VPC)^18^. The highly conserved mutations are predicted to localize to a loop near the active site (Fig. 2d). This loop interacts with the backbone of the single-stranded DNA substrate near the target base and supports productive orientation of the base relative to the catalytic zinc ion. Other conserved mutations, including A158S and Q154R, also mapped to the interface of TadA and the single-stranded DNA substrate. A structural prediction of TadA-CDa using AlphaFold2^57,58^ suggests that the mutations are not predicted to alter the structure of TadA compared to the cryo-EM structure of ABE8e (6VPC; Supplementary Fig. 4). Instead, the observed mutation of residues 26–28 from Arg-Glu-Val to smaller amino acids such as Gly-Ala-Gly during evolution may alleviate the steric clash that otherwise is predicted to block proper positioning of the pyrimidine C_4_ for nucleophilic attack and deamination (Supplementary Fig. 4). These observations collectively suggest that the evolved mutations may alter the conformation of the bound DNA substrate to enable efficient cytidine deamination and impede adenosine deamination.

We next performed mutagenesis and reversion analysis to interrogate the roles of the mutations found through evolution. In isolation, none of the mutations are sufficient to alter selectivity (Supplementary Fig. 5). However, the addition of just two mutations to the loop region (E27A V28G in TadCBEa–c,e and E27K V28A in TadCBEd) is sufficient to alter the selectivity of TadCBEs to modestly favor cytidine deamination, albeit with low editing efficiency (Supplementary Fig. 5). Additional mutations evolved during PANCE or PACE greatly increase activity and improve selectivity for C•G-to-T•A conversion. The reversion of mutations outside of the loop region generally decreases activity but not selectivity (Supplementary Fig. 6). This reversion analysis thus supports the importance of residues 26–28 in modulating the deamination selectivity of evolved TadA variants.

### Characterization of TadA-CDs in mammalian cells

**Fig. 3:**
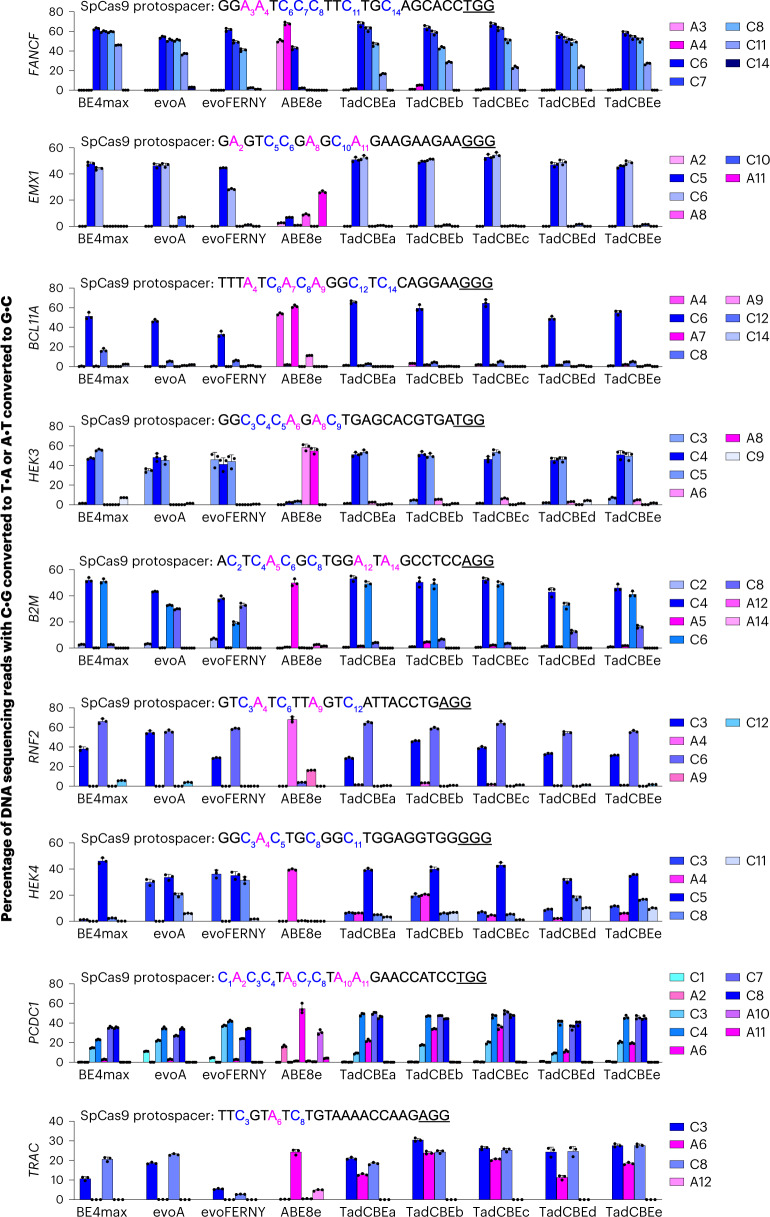
Characterization of evolved TadCBEs with SpCas9 domains in mammalian cells. The specified base editors using SpCas9 nickase domains in the BE4max architecture or ABE8e with 2×UGI were transfected along with each of nine guide RNAs targeting the protospacers shown in each graph. Target cytosines are blue, target adenines are magenta, and PAM sequences are underlined. C•G-to-T•A base editing is shown in shades of blue. A•T-to-G•C base editing is shown in shades of magenta. Dots represent individual values, and bars represent mean ± s.d. of three independent biological replicates. HEK293T site 3 is abbreviated *HEK3*, and HEK293T site 4 is abbreviated *HEK4*. Source data

Encouraged by the characteristics of the TadA-CDs in bacteria, we evaluated the evolved TadCBEs in mammalian cells. We cloned five TadCBE variants (TadCBEa–e) into mammalian expression vectors regulated by a cytomegalovirus (CMV) promoter in the BE4max architecture^48^. These five TadCBE variants were assayed alongside three of the most widely used engineered and evolved CBEs: BE4max^48^, evoA^12^ and evoFERNY^12^. We co-transfected HEK293T cells with each base editor plasmid and a single guide RNA (sgRNA) plasmid, allowed editing to occur for 72 hours and then sequenced target sites from genomic DNA. Across nine different target sites tested in HEK293T cells, TadCBE variants generally yielded target C•G-to-T•A editing (averaging 51–60% peak editing for TadCBEa–e across all nine tested sites) that was similar to or higher than that observed from canonical BE4max, evoA and evoFERNY CBEs (averaging 47%, 55% and 41% peak editing, respectively, across all nine sites) (Fig. 3 and Supplementary Fig. 7). These results demonstrate that TadCBEs can perform highly efficient C•G-to-T•A editing in mammalian cells.

Evolved TadCBE variants generally showed low residual A•T-to-G•C editing, averaging 1.5–4.5% editing for TadCBEa–e across adenosines in all nine tested sites and, thus, excellent selectivity for C•G-to-T•A editing over A•T-to-G•C editing (Fig. 3). By comparison, ABE8e in the same base editor architecture (with 2×UGI) averaged 31% A•T-to-G•C editing and 2.0% C•G-to-T•A editing across the nine sites. Ratios of desired C•G-to-T•A editing to residual A•T-to-G•C editing for seven of the nine tested sites was very high, averaging 21-fold to 42-fold for TadCBE variants a, c, d and e and 9.2-fold for TadCBEb (Fig. 3). Taken together, these observations suggest that residual A•T-to-G•C editing is generally low among evolved TadCBE variants, limited primarily to a small subset of target sites, protospacer positions and TadCBE variants. The introduction of V106W in the deaminase domain can further reduce residual A•T-to-G•C editing when necessary (vide infra).

### On-target and off-target editing by TadCBEs

Highly active cytidine deaminases that natively modify DNA, such as APOBEC family enzymes, can deaminate transiently exposed single-stranded DNA beyond those in the R-loop defined by Cas9, leading to low-level but widespread Cas-independent modification of the genome^13–15,19^. Likewise, high-activity cytidine deaminases that can potently engage RNA can also mediate unguided off-target RNA deamination^21^. Cas-independent off-target DNA and RNA editing activity could limit the use of some CBEs in applications for which off-target editing must be minimized^15^. Cas-independent off-target DNA editing has been found to be undetected or much less frequent for several TadA*-based ABEs^13^, although overexpression of some ABEs can result in low-level RNA deamination^6,7,34^.

The TadA origin of TadCBEs offers several advantages for minimizing off-target editing, including the potential to include mutations that were found to reduce off-target DNA or RNA editing in previous TadA engineering efforts^34,59,60^. For ABEs, the addition of V106W to TadA-7.10, TadA-8e or TadA-8.17-m reduced Cas-independent off-target editing of DNA and RNA in all three cases while maintaining high levels of on-target activity^6,7,34^. We sought to test whether the V106W mutation when introduced into TadCBEs could reduce off-target DNA or RNA editing while maintaining on-target activity and selectivity. Because several evolved mutations in TadA-CDs are proximal to V106, it was not clear if the addition of V106W would disrupt desired TadA-CD properties (Supplementary Fig. 8).

**Fig. 4:**
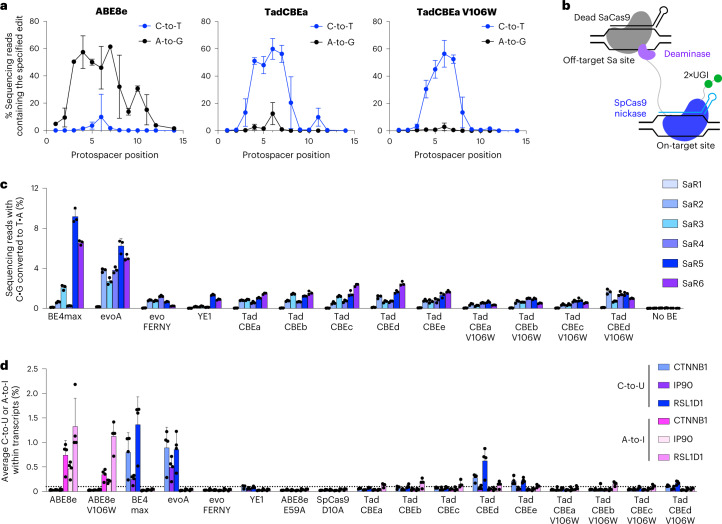
Characterization of base editing window and Cas-independent off-target DNA and RNA editing by TadCBEs. **a**, Base editing activity window for ABE8e with 2×UGI, TadCBEa and TadCBEa V106W across nine different target genomic sites in HEK293T. Dots represent average editing across all sites containing the specified base at the indicated position within the protospacer. Individual data points used for this analysis are in Fig. 3 and Supplementary Figs. 9 and 11. **b**, Method for measuring Cas-independent off-target DNA editing with the orthogonal R-loop assay. **c**, Average Cas-independent off-target editing across all cytosines within six orthogonal R-loops (SaR1–SaR6) generated by dead *S. aureus* Cas9. **d**, Off-target RNA editing. RNA was harvested from HEK293T cells 48 hours after transfection with the indicated base editor. After cDNA synthesis, *CTNNB1*, *IP90* and *RSL1D1* were amplified and analyzed by high-throughput sequencing. For **c** and **d**, dots represent individual biological replicates, and bars represent mean ± s.d. of three (**c**) or four (**d**) independent biological replicates. Source data

We first evaluated the on-target activity of TadCBEs containing V106W. We constructed V106W variants of TadCBEa–e and evaluated editing efficiency at nine target sites in HEK293T cells. TadCBE variants a–e tolerated the addition of V106W and maintained high on-target cytidine deamination activity, averaging 56% peak C•G-to-T•A target editing efficiency across the nine tested target sites for TadCBEa–e V106W, nearly matching 57% average peak editing efficiency for TadCBEa–e (Fig. 4a and Supplementary Figs. 9–12). The TadCBEa–e V106W variants exhibited a slightly narrower editing window than TadCBEa–e while maintaining high peak editing efficiency (Supplementary Fig. 12). Encouragingly, cytosine versus adenine base editing selectivity was improved 3.1-fold on average for TadCBE V106W variants compared to the corresponding TadCBE variants across these nine sites (Supplementary Fig. 12). TadCBE-V106W variants, thus, can retain efficient cytosine base editing with improved selectivity for deoxycytidine over deoxyadenosine deamination and refined editing windows.

Next, we evaluated Cas-independent DNA editing by TadCBEs and TadCBE-V106W variants using the previously established orthogonal R-loop assay^15,19^ (Fig. 4b). This assay measures the propensity of a base editor to modify single-stranded DNA in an off-target R-loop generated by an orthogonal, catalytically inactive *Staphylococcus aureus* Cas9 (SaCas9). By sequencing genomic DNA across six unrelated off-target SaCas9 R-loops, we determined that TadCBEs, on average, have 3.7-fold lower Cas-independent off-target C•G-to-T•A editing (0.84%–1.2%) compared to BE4max (3.6%) and evoA (3.8%) (Fig. 4c and Supplementary Figs. 13–16). The average off-target activity of evoFERNY (0.58%) and YE1 (0.53%) were also low. The addition of V106W further reduced Cas-independent off-target editing of TadCBEs by an average factor of 1.9 (to 0.38%, 0.62% 0.48%, 1.1% and 0.11% for V106W TadCBE variants a–e, respectively). Consistent with the selectivity of TadCBEs for deoxycytidine deamination, we did not detect appreciable off-target A•T-to-G•C editing by any TadCBEs (Supplementary Fig. 17). These findings indicate that evolved TadCBEs have inherently low Cas-independent off-target DNA editing that can be further suppressed by adding V106W while retaining high on-target C•G-to-T•A editing and low residual A•T-to-G•C editing.

We also evaluated off-target RNA editing by TadCBEs (Fig. 4d and Supplementary Figs. 18 and 19). After transfection of HEK293T cells by TadCBEa–e, BE4max, evoA, evoFERNY, ABE8e or ABE8e-V106W, RNA was extracted from cells. After complementary DNA (cDNA) synthesis, three target transcripts (*CTNNB1*, *IP90* and *RSL1D1*) previously used to measure off-target RNA editing due to their abundance or sequence similarity to the native TadA tRNA^Arg2^ substrate^2,15,19,34^ were amplified by RT–PCR and analyzed for C-to-U or A-to-I editing by high-throughput sequencing. Although BE4max and evoA edited, on average, ~0.7% of the analyzed cytosines in these transcripts, evoFERNY, YE1, TadCBEa, TadCBEb and TadCBEc all edited ≤0.1% of the cytosines (our limit of detection) (Fig. 4d and Supplementary Fig. 18). TadCBEd and TadCBEe edited, on average, 0.3% and 0.2% of cytosines across the three transcripts, respectively. The addition of V106W reduced average off-target RNA editing down to ≤0.13% in both cases (Fig. 4d and Supplementary Fig. 18).

Taken together, these data suggest that TadCBEs offer much lower frequencies of Cas-independent off-target DNA and RNA editing compared to BE4max and evoA. Off-target editing by TadCBEs is substantially less frequent than that of any other CBE of similar on-target activity and size. When further reduction of off-target editing is essential, the addition of V106W minimizes off-target DNA and RNA editing, focuses the editing window to ~4–5 base pairs and minimizes residual deoxyadenosine deamination, with only a small reduction in maximal on-target activity.

Finally, Cas-dependent off-target editing occurs when base editors engage a non-target site that resembles the target site through imperfect Cas9 binding^61^. We analyzed Cas-dependent off-target activity in HEK293T cells at 22 known off-target sites for SpCas9 base editors and sgRNAs targeting HEK293T site 3 (hereafter referred to as *HEK3*), HEK293T site 4 (hereafter referred to as *HEK4*), *EMX1* and *BCL11A* (Supplementary Figs. 20–25). Across multiple validated off-target sites, we observed that Cas-dependent off-target editing by TadCBEs was generally similar to the low level observed for BE4max and evoA variants (Supplementary Figs. 20–25). The Cas-dependent off-target activity of YE1 and evoFERNY was still lower, consistent with the lower on-target activity of these variants (Supplementary Figs. 20–25).

Collectively, these findings suggest that TadCBEs offer lower Cas-independent off-target DNA and RNA editing compared to canonical CBEs and low levels of Cas-dependent off-target DNA editing consistent with those observed for currently used CBEs of similar on-target editing efficiencies. The use of high-fidelity Cas proteins that engage fewer off-target loci is known to reduce Cas-dependent off-target DNA base editing^62^, and their use in TadCBEs may offer the same benefits.

### Characterization of TadCBEs on 10,638 target sites

TadCBE activity can vary substantially by target site (Fig. 3). To comprehensively characterize the activity of TadCBEs across a wide range of sites in mammalian cells, we performed high-throughput analysis of base editing outcomes for TadCBE variants using our previously reported ‘comprehensive context library’ of 10,638 paired sgRNA and target sites integrated into an mESC line (Supplementary Fig. 26)^11^. These libraries include target sites with all possible 6-mers surrounding a substrate A or C nucleotide at protospacer position 6 and all possible 5-mers across positions −1 to 13 (counting the position immediately upstream of the protospacer as position 0) with minimal sequence bias^11^. Base editing conditions were optimized to allow differences between base editors to be detected. We maintained an average cell coverage of ≥300× per library member throughout the course of the experiment and an average sequencing depth of ≥2,800× per target, which enabled us to detect editing outcomes with high sensitivity. We collected two biological replicates per base editor for TadCBEa–e, V106W variants of TadCBEa–d, TadDE, and BE4max as a reference^11^, and validated that the library assay data have strong consistency between biological replicates (Supplementary Fig. 27).

**Fig. 5:**
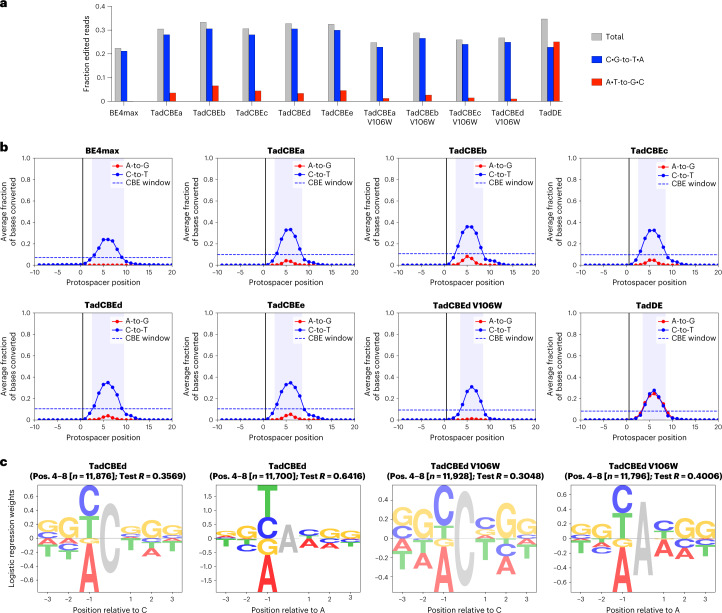
Characterization of TadCBEs using a genomically integrated mESC target sequence library. **a**, Overall efficiency and selectivity of base editors analyzed through editing of the library. Data show the average fraction of edited sequencing reads across all library members between protospacer positions −9 to 20, where positions 21–23 are the PAM. **b**, BE4max, TadCBEa–e, TadCBEd V106W and TadDE editing profiles across 10,638 genomically integrated target sites. The editing window is defined as the protospacer positions for which average editing efficiency is ≥30% of the average peak editing efficiency. Window plots for all variants tested in the library experiment can be found in Supplementary Fig. 28. **c**, Sequence motifs of TadCBEd and TadCBEd V106W for cytosine and adenine base editing outcomes determined by performing regression on editing efficiencies. Opacity of sequence motifs is proportional to the test *R* on a held-out set of sequences. Complete sequence motif plots for all variants are in Supplementary Fig. 30. Source data

We used the resulting library data to quantify editing activity and C•G-to-T•A selectivity for each TadCBE (Fig. 5a). Across the 10,638 integrated target sites, all TadCBE and TadCBE-V106W variants edited with greater average efficiency (28–31% of reads on average with any C•G-to-T•A editing) than BE4max (21%) (Fig. 5a)^11^. We next characterized the editing windows, which we defined as positions within the protospacer that averaged ≥30% of the peak average editing efficiency (Fig. 5b and Supplementary Fig. 28). TadCBE editing is generally centered around protospacer position 6. The most active variant, TadCBEd, has a similar editing window (protospacer positions 3–9) to that of BE4max (positions 3–9), whereas the remaining TadCBEs and V106W-TadCBEs have slightly narrower windows (positions 3–8; Fig. 5b and Supplementary Fig. 28).

TadCBE selectivity for cytosine editing over adenine editing varied by base editor. Among the canonical TadCBEs (without V106W), TadCBEd showed the highest C•G-to-T•A selectivity, with a geometric mean of the ratio of C•G-to-T•A versus A•T-to-G•C editing at each position in its editing window of 26.8 (Methods and Supplementary Table 1). Notably, the addition of V106W substantially improved C•G-to-T•A selectivity for all TadCBE variants (TadCBEd V106W selectivity = 47.8) while minimally affecting base editing activity at the maximally edited position (Fig. 5a,b and Supplementary Fig. 29). For example, the addition of V106W to TadCBEd reduced peak editing among the library targets from 35% to 31%.

Consistent with the discrete target site examples shown above, C•G-to-T•A selectivity of TadCBEs varied by target site across the comprehensive context library. Adenine base editing was observed in 3.4–6.6% of reads (on average) for TadCBEs and 1.0–2.7% of reads for V106W-TadCBEs across all target sites in the comprehensive context library (Fig. 5a). We generated sequence motifs by performing regression on the editing efficiencies to determine the sequence characteristics that affect cytosine and adenine deamination (Methods, Fig. 5c and Supplementary Fig. 30). TadCBEs have similar sequence context preferences to their ancestor, ABE7.10, favoring editing of cytosine and adenine bases preceded by 5′ Y (Y = T/C) while disfavoring 5′ A (ref. ^11^). When performing bystander adenine base editing, TadCBEs retain the sequence context preference of ABE7.10 (favoring 5′ YAY and disfavoring 5′ AAA). However, TadCBEs instead slightly disfavor 5′ ACT. The difference in 3′ preference may be due to differences in substrate positioning required to achieve altered selectivity, because interactions with adjacent bases could alter placement of the target cytidine in the active site (Supplementary Fig. 4).

TadDE performs very similar levels of adenine and cytosine base editing (ABE:CBE ratio = 1.1) and has similar sequence context dependence to TadCBEs (Fig. 5a,b, Supplementary Fig. 30 and Supplementary Table 1). TadDE is highly efficient, editing 35% of the reads on average in the library experiment (Fig. 5a). The probability of observing A•T-to-G•C editing given that C•G-to-T•A editing is observed is 0.62 for TadDE compared to 0.04 for TadCBEd-V106W, our most selective TadCBE variant (Supplementary Table 1). The high activity, promiscuity and small size of TadDE makes it a promising tool for concurrent A•T-to-G•C and C•G-to-T•A editing.

Collectively, these data show that TadCBEs have greater cytosine deamination activity than conventional narrow-window CBEs. Furthermore, the introduction of V106W in the deaminase domain reduces residual A•T-to-G•C editing activity while minimally impacting C•G-to-T•A editing for all TadCBEs in this experiment. Overall, TadCBEd enables the greatest cytosine deamination activity with high C•G-to-T•A selectivity, which is further improved by the addition of V106W.

### TadCBE compatibility with Cas9 orthologs

The use of Cas9 orthologs with diverse PAM requirements expands the targetable sequence space of base editors. To test if TadCBEs are compatible with Cas9 homologs beyond *Streptococcus pyogenes* Cas9, we constructed TadCBE variants with PACE-evolved variants of Nme2Cas9 from *Neisseria meningitidis* that broaden the scope of accessible PAMs beyond the canonical NGG PAM of SpCas9 (ref. ^63^). We recently evolved Nme2Cas9 variants that access a wide range of single-pyrimidine PAM sites as nucleases or as base editors^64^. We generated fusions of TadA-CDs with an eNme2-C variant nickase (PAM = N_4_CN) and two UGI domains, co-transfected the resulting eNme2-C-TadCBEs with a guide RNA plasmid and examined base editing at six genomic loci in HEK293T cells. Across all tested sites, the peak editing efficiency of TadCBEs was similar to that of BE4max, evoFERNY and evoA (Supplementary Figs. 31 and 32). Although C•G-to-T•A editing exceeded 50% at some sites, residual A•T-to-G•C editing never exceeded 5.3% at any of the six eNme2 target sites tested. TadCBEs thus exhibited robust activity and selectivity with eNme2-CCas9 variants.

We next tested TadCBEs with SaCas9 in the BE4max architecture^65^. SaCas9 (1,053 amino acids) is smaller than SpCas9 (1,368 amino acids) and recognizes a different PAM sequence (PAM = NNGRRT). We found that TadCBEs using SaCas9 have robust C•G-to-T•A editing across nine sites (4.1–44%) with less than 5.5% A•T-to-G•C editing at any site (Supplementary Figs. 33 and 34). These observations suggest potential compatibility with other Cas proteins that, together with SpCas9, eNme2-C Cas9 and SaCas9, may offer access to a variety of PAM sequences for versatile targeting of TadCBEs. We additionally found that TadDE performed both A•T-to-G•C and C•G-to-T•A editing with SpCas9, eNme2-C Cas9 and SaCas9 in mammalian cells at sites where TadCBEs were selective, suggesting broad Cas9 compatibility of the dual editor as well (Supplementary Figs. 31–36).

TadCBEs exhibit a narrower editing window than BE4max, evoA and evoFERNY CBEs while maintaining similar or higher maximal editing efficiencies (Supplementary Fig. 31). For example, BE4max and evoA edited *Neisseria meningitidis* site 50 (hereafter referred to as *Nme50*) at protospacer positions 3–18 with 4.2–47% efficiency, whereas TadCBEa, TadCBEb and TadCBEc modify only the narrower position 3–8 window with 5–48% efficiency (Supplementary Fig. 31). The narrower base editing activity window of TadCBEs could arise from a less processive deaminase, because the processive APOBEC family deaminases can catalyze multiple hydrolytic deamination reactions per DNA-binding event^66^. Although a wide editing window can be useful for some applications, such as targeted gene disruption or base editing screens, the narrower window of TadCBEs should benefit precision editing applications in which modification of only one target base is desirable, particularly when using Cas9 domains that support a wider base editing window^63,67^. Taken together, the small size of TadCBEs, their compatibility with eNmeCas9 and SaCas9, their more focused editing windows and their high editing efficiencies and selectivities for cytosine over adenine base editing demonstrate their suitability for a variety of precision cytosine base editing applications.

### Multiplexed base editing in primary human T cells

**Fig. 6:**
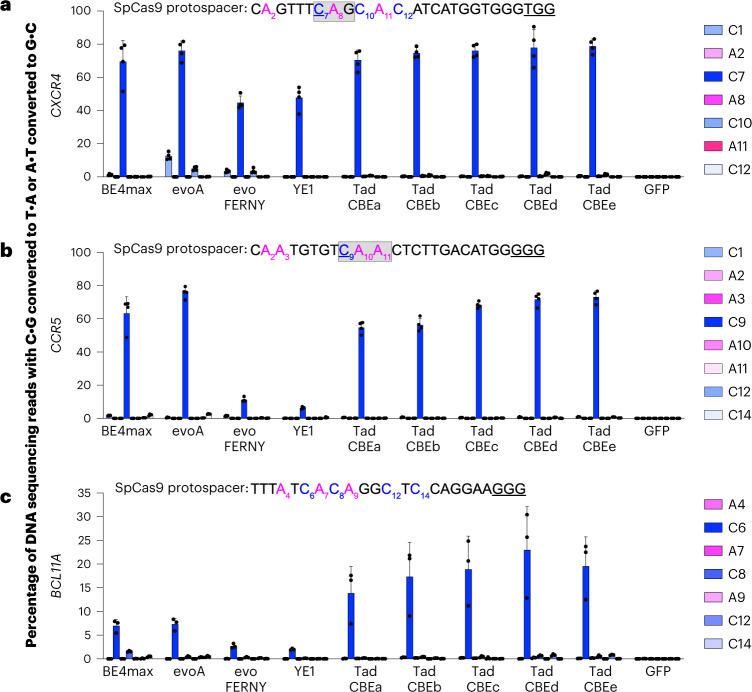
Base editing at therapeutically relevant loci by TadCBEs in primary human T cells and HSPCs. mRNA encoding the indicated base editor or GFP as a negative control was electroporated into human T cells (*n* = 4 donors) along with two synthetic guide RNAs targeting *CXCR4* (**a**) or *CCR5* (**b**) at the specified protospacers. Target cytosines are blue, target adenines are magenta, and PAM sequences are underlined. After 3 days, genomic DNA was harvested from T cell lysates and analyzed by high-throughput sequencing. The gray boxes indicate the desired location of stop codon installation in *CXCR4* and *CCR5*. The targeted cytidine to yield TAG (*CXCR4*) and TAA (*CCR5*) stop codons upon cytosine base editing is underlined. **c**, mRNA encoding the indicated base editor or GFP as a negative control was electroporated into HSPCs along with a synthetic guide RNA targeting the *BCL11A* enhancer. After 3 days, genomic DNA was harvested from cell lysates and analyzed by high-throughput sequencing. C•G-to-T•A base editing is shown in shades of blue, and A•T-to G•C-base editing is shown in shades of magenta. Dots represent individual biological replicates, and bars represent mean ± s.d. from *n* = 4 donors (**a** and **b**) or *n* = 3 donors (**c**). Source data

We evaluated whether TadCBEs can perform multiplexed editing of target loci in T cells in support of therapeutic applications. Multiplexed base editing in T cells can modify or disrupt multiple genes with minimized risks of chromosomal abnormalities and cell state perturbations that arise from multiple double-stranded breaks^68–72^. To determine whether TadCBEs can perform multiplexed editing in primary human T cells, we targeted the *CXCR4* and *CCR5* loci for simultaneous base editing to install premature stop codons in both HIV co-receptors (Fig. 6a,b)^73^. We performed in vitro transcription (IVT) of TadCBE variants a, b, c, d and e. We then electroporated the TadCBE mRNA along with guide RNAs targeting *CXCR4* and *CCR5* (Fig. 6a,b)^73^ into primary human T cells and analyzed editing efficiencies at both target sites.

TadCBEs performed efficient (averaging 70%) and selective editing of the target cytosines (C_7_ in *CXCR4* and C_9_ in *CCR5*), resulting in premature stop codon installation in each gene (Fig. 6a,b). Editing efficiencies of TadCBEs were similar to those of BE4max (67%) and evoA (76%) (Fig. 6a,b). Observed indel frequencies of all the tested base editors was comparably low (typically ≤0.68%; Supplementary Fig. 37). Consistent with data in HEK293T cells (Supplementary Fig. 12), TadCBEs exhibited a more precise editing window with fewer bystander edits at *CXCR4* and *CCR5* in primary human T cells. Because TadCBEs maintain high editing efficiencies and product purities but offer substantially lower Cas-independent off-target DNA and RNA editing than APOBEC and evoA (Fig. 4c,d and Supplementary Figs. 13–17), TadCBEs provide a promising alternative for multiplexed cytosine base editing of T cells.

We also compared T cell editing by TadCBEs to that of evoFERNY and YE1, which offer similarly low off-target editing as TadCBEs (Figs. 6a,b and 4c,d and Supplementary Figs. 13–17). TadCBEs supported substantially higher editing efficiencies in T cells than evoFERNY and YE1. At *CXCR4*, target C•G-to-T•A editing efficiency by TadCBEs averaged 1.5-fold to 1.7-fold that of evoFERNY and YE1, whereas, at *CCR5*, average TadCBE editing efficiencies were 4.9-fold to 11-fold higher on average. We analyzed three known Cas-dependent off-target sites for the *CCR5* guide RNA and one known off-target for *CXCR4* and found that Cas-dependent off-target editing was lower for TadCBEa–e, evoFERNY and YE1 (≤0.12%) than for BE4max (0.1–0.58%) and evoA (0.1–1.0%) (Supplementary Fig. 38). Next, we tested V106W variants of TadCBEa–e in T cells. Relative to their TadCBE counterparts, the V106W variants displayed 1.3-fold to 1.9-fold lower average activity at C_7_ of *CXCR4* and 1.4-fold to 3.3-fold lower average activity at C_9_ of CCR5, with a proportional drop in C•G-to-G•C editing (Supplementary Figs. 39–41). These data are consistent with the narrower editing window of V106W variants and suggest that the more transient mRNA delivery of TadCBEs may reveal a greater range of editing activity compared to plasmid transfections of HEK293T cells. Overall, these findings demonstrate that TadCBEs offer a favorable combination of on-target and off-target editing features compared to currently used CBEs when base editing primary human T cells at target sites of therapeutic relevance.

### Editing in human HSPCs

Finally, we evaluated the editing efficiency of TadCBEs in human HSPCs. We electroporated TadCBEa–e mRNA along with a synthetic guide targeting the enhancer of *BCL11A* into primary human CD34^+^ cells. Mutations at the enhancer can decrease the expression of *BCL11A*, leading to induction of fetal hemoglobin expression as a potential treatment for sickle cell disease^74,75^. For comparison, we electroporated mRNA encoding BE4max, evoA, evoFERNY, YE1 or GFP (as a negative control) in parallel. evoFERNY and YE1 yielded only 2.7% and 2.0% average editing, respectively, whereas BE4max and evoA averaged 7.0% and 7.4% editing efficiencies, respectively (Fig. 6c). All five of the tested TadCBEs supported 2–3-fold-higher editing efficiencies than BE4max or evoA, averaging 14–23% (Fig. 6c). All of the tested CBEs yielded low levels of indels (≤1.1%; Supplementary Fig. 42a) and Cas-dependent off-target editing (≤0.87%; Supplementary Fig. 42b). These results demonstrate that the editing efficiencies of TadCBEs can exceed that of the most commonly used CBEs for some therapeutically relevant sites and cell types.

## Discussion

TadA has been evolved and engineered in the laboratory from a tRNA-editing enzyme found in *E. coli* into widely used ABEs, including several that are already in the clinic^9^ or headed to clinical trials^10^. Evolved TadA variants offer many characteristics that are beneficial for precision gene editing applications, including some features not previously present in CBEs. The evolution of TadA variants that catalyze efficient and selective cytidine deamination in this study enabled the development of TadCBEs, a class of CBEs that offer high on-target editing, low off-target Cas-independent and Cas-dependent DNA editing, low off-target RNA editing and size small enough to fit into a single AAV^27,28^. In HEK293T cells, TadCBEs perform highly efficient C•G-to-T•A editing across a range of sites with SpCas9, eNme-2C variants and SaCas9. These results demonstrate directed evolution of a deaminase to selectively deaminate a different base rather than simply relaxing target base specificity—an outcome of the simultaneous positive and negative selection system that evolved selective TadCBE deaminases.

A side-by-side comparison with commonly used CBEs revealed that TadCBEs offer unique properties that make them well-suited for applications where canonical BE4max, evoA, evoFERNY and YE1 may face limitations. The narrow editing window of TadCBEs is beneficial when precision editing is required. Despite having similar on-target editing efficiencies as BE4max and evoA, TadCBEs exhibit lower Cas-independent off-target DNA and RNA editing. evoFERNY and YE1 also exhibit low Cas-independent editing but display different editing profiles and achieve substantially lower editing efficiency at some target loci, including *CXCR4* and *CCR5* in T cells and *BCL11A* in HSPCs. The evolution of TadA-CDs from TadA-8e, therefore, extends the utility of TadA for gene editing, demonstrates a new strategy for generating base editors and provides a family of CBEs with favorable editing properties.

Based on these findings, we recommend TadCBEd, which offers the highest on-target editing and selectivity of the TadCBE variants, for general cytosine base editing applications. When off-target DNA or RNA editing or residual A•T-to-G•C editing must be kept to an absolute minimum, we recommend TadCBEd-V106W.

## Methods

### General methods and molecular cloning

Gibson assembly (New England Biolabs), USER cloning (New England Biolabs) or SapI-Golden Gate (New England Biolabs) was used to carry out all plasmid construction. Nuclease-free water (Qiagen) was used for PCR reactions and cloning. For all other experiments, water was purified using a MilliQ purification system (Millipore). PCR was performed using Phusion HiFi polymerase or Phusion U Green Hot Start II DNA polymerase (Thermo Fisher Scientific). After Gibson, USER or Golden Gate cloning, cloning products were transformed into Mach1 chemically competent *E. coli* (Themo Fisher Scientific). Selection antibiotics were used at the following final concentrations: carbenicillin: 100 μg ml^−1^; spectinomycin: 50 μg ml^−1^; kanamycin: 50 μg ml^−1^; chloramphenicol: 25 μg ml^−1^; and tetracycline: 10 μg ml^−1^. Plasmid DNA was amplified using the Illustra Templiphi 100 Amplification Kit (GE Healthcare Life Sciences) before Sanger sequencing (Quintara Biosciences). Sequence-confirmed plasmids for bacterial transformation were purified using the Miniprep Kit (Qiagen). Plasmids for mammalian transfection were purified using the Plasmid Plus Midi Kit (Qiagen) according to the manufacturer’s instructions. Plasmid concentrations were quantified by NanoDrop. The amino acid sequences of all CBE and ABE variants are listed in Supplementary Notes 1 and 2. A full list of bacterial plasmids used in this work is provided in Supplementary Table 2.

### Bacteriophage cloning

For USER assembly of phage, 0.2 pmol of each PCR fragment was added to a final volume of 20 µl. After USER assembly, the 20-µl USER reaction was transformed into 100 µl of chemically competent S2060 *E. coli* host cells containing pJC175e^46^. For Gibson assembly of phage, 0.2 pmol of each PCR fragment was added to make up a final volume of 20 µl. After Gibson assembly, the 20-µl Gibson reaction was transformed into 100 µl of chemically competent S2060 *E. coli* host cells containing pJC175e^46^. Cells transformed with pJC175e enable activity-independent phage propagation and were grown for 5 hours at 37 °C with shaking in antibiotic-free 2×YT media. Bacteria were then centrifuged for 1 minute at 10,000 *g* and plaqued as described below to isolate clonal phage populations. Individual plaques were grown in DRM media (prepared from United States Biological CS050H-001/CS050H-003) for 6–8 hours. Bacteria were centrifuged for 10 minutes at 6,000 *g* to remove *E. coli* from the supernatant. The supernatant containing the phage was filtered through a 0.22-µm PVDF Ultrafree centrifugal filter (Millipore) to remove residual bacteria. For sequencing, the gene of interest within the phage was amplified with primers AB1793 (5′-TAATGGAAACTTCCTCATGAAAAAGTCTTTAG) and AB1396 (5′-ACAGAGAGAATAACATAAAAACAGGGAAGC), and the PCR product was sequenced by Sanger sequencing (Quintara Biosciences). The primers (Integrated DNA Technologies) anneal to the phage backbone, flanking the evolving gene of interest. Sequence-confirmed phage were stored at 4 °C.

### Preparation and transformation of chemically competent cells

Strain S2060 (ref. ^76^) was used in all phage propagation, PANCE and PACE experiments. To prepare competent cells, an overnight culture was diluted 250-fold into 50 ml of 2×YT media (United States Biological) supplemented with tetracycline and grown at 37 °C with shaking at 230 r.p.m. to OD_600_ ~0.4–0.6 and then incubated on ice for 20 minutes. Cells were then pelleted by centrifugation at 4,000 *g* for 10 minutes at 4 °C. The cell pellet was resuspended by the addition of 5 ml of TSS (LB media supplemented with 5% v/v DMSO, 10% w/v PEG 3350 and 20 mM MgCl_2_). The cell suspension was pipetted gently to mix completely, aliquoted into 100-µl volumes, flash-frozen in liquid nitrogen and stored at −80 °C.

To transform cells, 100 μl of competent cells thawed on ice was added to a pre-chilled mixture of plasmid (1–2 μl each, up to three plasmids per transformation) in 20 μl of 5× KCM solution (500 mM KCl, 150 mM CaCl_2_ and 250 mM MgCl_2_ in water) and 80 μl of water and mixed gently by pipetting. The mixture was incubated on ice for 15 minutes and heat-shocked at 42 °C for 90 seconds before adding 800 μl of SOC media (New England Biolabs) to rescue. Cells were allowed to recover at 37 °C with shaking at 230 r.p.m. for 1–1.5 hours, plated on 2×YT media + 1.5% agar (United States Biological) containing the appropriate antibiotics and incubated at 37 °C for 16–18 hours.

### Plaque assays for phage titer quantification and cloning

Phage were plaqued on S2060 *E. coli* host cells containing the pJC175e plasmid to enable activity-independent propagation^46^. To prepare *E. coli* host cells at the appropriate growth phage for plaquing, an overnight culture of host cells (fresh or stored at 4 °C for up to 3 days) was diluted 50-fold in DRM containing the appropriate antibiotics. Cells were grown at 37 °C to an OD_600_ of 0.8–1.0 (~2 hours), at which point they were moved to an ice bucket during preparation of the phage. Phage stocks were serially diluted with DRM by a factor of 10 (up to 10^6^-fold). To prepare plates for plaquing, molten 2×YT agar (1.5% agar, 55 °C) was mixed with Bluo-gal (Gold Biotechnology, 4% w/v in DCM) to a final concentration of 0.08% Bluo-gal. The molten agar mixture was pipetted into quadrants of a quartered Petri dish (2 ml per quadrant) and left at room temperature for 5 minutes to solidify. To prepare top agar, a 3:2 mixture of 2×YT medium and molten 2×YT medium agar (1.5%, resulting in a 0.6% agar final concentration) was prepared and stored at 55 °C until use. To plaque, 100 µl of cells were mixed with 10 µl of phage in 2-ml library tubes (VWR International). Then, 900 µl of warm top agar was added to the cell and phage mixture, pipetted to mix and then immediately pipetted onto the solid agar medium in one quarter of the petri dish. Top agar was allowed to set undisturbed for 2 minutes at 25 °C. Plates were then incubated, without inverting, at 37 °C overnight. Phage titers were determined by quantifying blue plaques. For higher-throughput plaquing, the reagents were adjusted for the wells of a 12-well plate as follows: 900 µl of bottom agar, 450 µl of top agar, 10 µl of phage and 100 µl of cells.

### Phage overnight propagation assays

S2060 cells transformed with the AP and CP plasmids of interest were prepared as described above and inoculated into DRM. Cells were grown overnight. The next day, host cells were diluted 50-fold into fresh DRM and grown at 37 °C to an OD_600_ of 0.3–0.5. Host cells were distributed into the wells of a 96-well plate (1 ml per well, Axygen), and phage of a known titer were then added to an input concentration of 10^5^ plaque-forming units per milliliter (PFU ml^−1^^)^. The cultures were grown overnight (14–20 hours) with shaking at 230 r.p.m. at 37 °C. Plates were then centrifuged at 4,000 *g* for 10 minutes to remove cells, leaving phage in the supernatant. The supernatants were then titered by plaquing as described above. Fold enrichment was calculated by dividing the output propagated phage titer by the input phage titer.

### PANCE

PANCE experiments were performed according to published protocols^77^. S2060 host cells transformed with AP and CP were made chemically competent as described above. Chemically competent host cells were transformed with mutagenesis plasmid (MP6)^47^ and plated on 2×YT agar containing 100 mM glucose along with the appropriate antibiotics. Between four and eight colonies were picked into individual wells of a 96-well plate containing 1 ml of DRM and the appropriate antibiotics. The colonies were resuspended and serially diluted ten-fold, eight times into DRM. The plate was sealed with a porous sealing film and grown at 37 °C with shaking at 230 r.p.m. for 16–18 hours. Wells containing dilutions with OD_600_ ~0.3–0.4 were combined, treated with 20 mM arabinose to induce mutagenesis and distributed into the desired number of 1-ml cultures in a 96-well plate. The cultures were then inoculated with selection phage at the indicated dilution (Supplementary Fig 2). Infected cultures were grown for 12–18 hours at 37 °C and harvested the next day by centrifugation at 4,000 *g* for 10 minutes. Then, 100 µl of the supernatant containing the evolved phage was transferred to a 96-well PCR plate, sealed with foil and stored at 4 °C. Isolated phage were then used to infect the next passage, and the process was repeated for the duration of the selection. Phage titers were determined by qPCR as described previously^77^ or by the plaque assay as described above. The sequences of the promoters and ribosome binding sites used during evolution are listed in Supplementary Table 3.

### PACE

PACE experiments were performed according to previously published protocols^77^. Host cells containing the mutagenesis plasmid were prepared as described for PANCE above. Twelve colonies were picked into individual wells of a 96-well plate containing 1 ml of DRM and the appropriate antibiotics. The colonies were resuspended and serially diluted by a factor of ten, eight times into DRM. The plate was sealed with a porous sealing film and grown at 37 °C with shaking at 230 r.p.m. for 16–18 hours. Wells containing dilutions with OD_600_ ~0.3–0.4 were combined and used to inoculate a chemostat containing 100 ml of DRM. The chemostat was grown to OD_600_ ~0.4–0.8 and then continuously diluted with fresh DRM at a rate of 1–1.5 chemostat volumes per hour to keep the cell density constant. The chemostat was maintained at a volume of 80–100 ml.

Before SP infection, lagoons were filled with 15 ml of culture from the chemostat and pre-induced with 10 mM arabinose for at least 1 hour. Lagoons were infected with SP at a starting titer of 10^8^ PFU ml^−1^. To increase stringency, the lagoon dilution rates increased over time as indicated in Supplementary Fig. 3. During the evolution, samples (800 µl) of the SP were collected from the lagoon waste lines at the indicated times. Samples were centrifuged at 6,000 *g* for 10 minutes, and the supernatant was stored at 4 °C. Titers of SP samples were determined by plaque assays using S2060 cells transformed with pJC175e^46^. The sequences of individual plaques were determined by PCR amplification with the AB1793/AB1396 primer pair, followed by Sanger Sequencing, as described above in the ‘Bacteriophage cloning’ methods. Mutation analyses were performed using Mutato. Mutato is available as a Docker image at https://hub.docker.com/r/araguram/mutato (ref. ^4^).

### High-throughput sequencing of plasmid editing in *E. coli*

To generate the base-editor-expressing cells, 20 µl of 10-beta electrocompetent *E. coli* (New England Biolabs) were distributed into each well of a 16-well Nucleocuvette strip. Target plasmid and editor plasmid (0.5 µl each at 100–200 ng µl^−1^) were added to each well, and *E. coli* were electroporated with a 4D-Nucleofector System (Lonza) using bacterial program X-5. Electroporated cells were immediately recovered in 120 µl of SOC media (New England Biolabs) by shaking at 230 r.p.m. at 37 °C for 1 hour. Cells were plated on the appropriate selection antibiotics, along with 100 mM glucose to suppress expression of the base editor, and incubated at 37 °C overnight. The next morning, single colonies were inoculated into 300 µl of DRM with antibiotic in separate wells of a 96-well plate (*n* = 4 replicates per condition). The plate was sealed with a porous sealing film, and cells were grown to saturation by shaking at 37 °C (~8 hours). Saturated cultures were diluted 1:50 into 1 ml of DRM with antibiotics and grown to mid-log phase (~1.5 hours). To induce expression of the base editor, arabinose was added to the cultures (30 mM final concentration), and cells were grown overnight at 37 °C with shaking at 230 r.p.m. After 16 hours, cells were resuspended by mixing with a multichannel pipette, and 60 µl from each well was transferred into a PCR plate. Cells were lysed by boiling at 95 °C for 8 minutes using a thermal cycler (Bio-Rad). Cell lysates were stored at −20 °C before analysis.

For high-throughput sequencing, 1 µl of *E. coli* lysate was used as a PCR template for amplification with the Nextera HTS primers (Illumina) to install adapters as indicated in Supplementary Table 4. Phusion HiFi polymerase (New England Biolabs) was used for amplification. Barcoding and high-throughput sequencing was performed as described for mammalian cell experiments below.

### General mammalian cell culture

HEK293T (American Type Culture Collection (ATCC), CRL-3216) cells were purchased from ATCC and cultured in Dulbecco’s Modified Eagle’s Medium (DMEM) plus GlutaMAX (Thermo Fisher Scientific) supplemented with 10% (v/v) FBS (Gibco, qualified). Undifferentiated 129P2/OlaHsd mESC (males) lines were maintained as previously described^11^. In brief, cells were maintained on gelatin-coated plates in mESC media (Knockout DMEM (Life Technologies) supplemented with 15% defined FBS (HyClone), 0.1 mM nonessential amino acids (Life Technologies), 1% Glutamax (Life Technologies), 0.55 mM 2-mercaptoethanol (Sigma-Aldrich) and 1× ESGRO LIF (Millipore), 5 nM GSK-3 inhibitor XV and 500 nM UO126). Cells were incubated, maintained and cultured at 37 °C with 5% CO_2_. Cell lines were authenticated by their respective suppliers and tested negative for mycoplasma.

### HEK293T cell transfection

Cells were seeded at a density of 1.5 × 10^4^ cells per well on 96-well plates (Corning) 16–24 hours before transfection. Transfection conditions were as follows: 0.5 µl of Lipofectamine 2000 (Thermo Fisher Scientific), 100 ng of editor plasmid and 40 ng of guide RNA plasmid were combined and diluted with Opti-MEM reduced serum media (Thermo Fisher Scientific) to a total volume of 12.5 µl and transfected according to the manufacturer’s protocol. Cells were transfected at approximately 60–80% confluency.

### Genomic DNA isolation from mammalian cell culture

After transfection, cells were cultured for 3 days, after which media was removed, cells were washed with 1× PBS solution (100 µl) and genomic DNA was harvested via cell lysis with 50 µl of lysis buffer added per well (10 mM Tris-HCl, pH 8.0, 0.05% SDS, 20 µg ml^−1^ of Proteinase K (New England Biolabs)). The cell lysis mixture was incubated for 1–1.5 hours at 37 °C before being transferred to 96-well PCR plates and enzyme-inactivated for 30 minutes at 80 °C. The resulting genomic DNA mixture was stored at −20 °C until analysis.

### Generation of base editor mRNA from IVT

Base editor mRNA was generated from PCR product amplified from a template plasmid containing an expression vector for the base editor of interest cloned as described previously^6^. PCR product was amplified in a 200 µl of total reaction using forward primer IVT-F and reverse primer IVT-R (Supplementary Table 5), purified using the QIAquick PCR Purification Kit (Qiagen) and eluted in 50 µl of nuclease-free water. IVT reactions were performed using the HiScribe T7 High-Yield RNA Synthesis Kit (New England Biolabs) according to the manufacturer’s protocols but with full substitution of *N*1-methyl-pseudouridine (TriLink BioTechnologies) in place of uridine and co-transcriptional capping with CleanCap AG (TriLink BioTechnologies). mRNA isolation was performed by lithium chloride precipitation. In brief, for 160 µl of IVT reaction, 0.5 volumes of 7.5 M lithium chloride was added (240-µl final volume) and mixed by pipetting. After incubation of the mixture at -20 °C for 30 minutes, samples were centrifuged at 15,000 *g* for 20 minutes. Supernatant was discarded, and pellet was resuspended with 400 µl of ice-cold 70% ethanol. Mixture was centrifuged at 4 °C for 15 minutes, and supernatant was discarded again. The resulting pellet was air-dried at room temperature for 5 minutes and then resuspended in 100–200 µl of nuclease-free water. An aliquot of the re-suspension was diluted five fold for quantification by NanoDrop. Samples were normalized to 2 µg µl^−1^ and stored at −80 °C.

### Electroporation of TadCBE mRNA and sgRNA into T cells or hematopoietic stem cells

Buffy coats from de-identified human donors (*n* = 4) were purchased from Memorial Blood Centers in St. Paul, Minnesota, and peripheral blood mononuclear cells were isolated using Lymphoprep and SepMate tubes (STEMCELL Technologies). From these, CD4^+^ cells were purified with the EasySep Human CD4^+^ T Cell Isolation Kit (STEMCELL Technologies), followed by activation with Dynabeads Human T-Expander CD3/CD28 beads (Thermo Fisher Scientific) and cultured in X-VIVO 15 Serum-free Hematopoietic Cell Medium (Lonza) that contained 5% AB human serum (Valley Biomedical), GlutaMAX (Gibco), *N*-acetylcysteine (Sigma-Aldrich), 50 U ml^−1^ of penicillin and 50 µg ml^−1^ of streptomycin (Gibco) and 300 IU ml^−1^ of IL-2. At 72 hours, the beads were removed, and 300,000 T cells were electroporated with 2 µg of candidate base editor mRNA and 100 pmol of sgRNA (Synthego) using the Neon Electroporation System with 10-µl tips (Thermo Fisher Scientific). Sequences of the chemically synthesized guide RNAs used are listed in Supplementary Table 6.

CD34^+^ cells without any identifying donor information were procured from the Core Center for Excellence in Hematology at the Fred Hutchinson Cancer Research Center and cultured in StemSpan SFEM II media (STEMCELL Technologies) containing 50 U ml^−1^ of penicillin and 50 µg ml^−1^ of streptomycin (Gibco), 100 ng ml^−1^ of each of recombinant human thrombopoietin, stem cell factor (TPO; BioLegend), Flt-3 ligand and IL-6 (PeproTech) and 0.75 µM StemRegenin1 and 500 nM UM729 (STEMCELL Technologies). At 48 hours after thawing (*n* = 3 donors), 2 µg of editor mRNA and 100 pmol of sgRNA were electroporated into 200,000 hematopoietic stem cells (HSCs) using the Amaxa (Lonza) 4D-Nucleofector protocol for P3 Primary Cell Line 4D Nucleofector Kit in Nucleovette strips, program DZ-100. Sequences of the chemically synthesized guide RNAs used are listed in Supplementary Table 6.

At 72 hours after gene transfer, cell pellets were harvested for DNA using the QuickExtractDNA Extraction Solution. PCR amplification for Illumina sequencing was performed using Phusion U Multiplex PCR Master Mix (Thermo Fisher Scientific) under the following conditions: 30 seconds at 98 °C; 30–35 cycles at 98 °C for 10 seconds, 64 °C for 30 seconds and 72 °C for 20 seconds; and a final extension at 72 °C for 5 minutes.

### High-throughput DNA sequencing of genomic DNA samples

High-throughput sequencing of genomic DNA from mammalian cell lines was performed as previously described^2^. Primers for PCR amplification of target genomic sites are listed in Supplementary Table 4. Sequences of the target amplicons are listed in Supplementary Table 4. DNA concentrations were quantified using a Qubit dsDNA High Sensitivity Assay Kit (Thermo Fisher Scientific) or by qPCR with the KAPA Library quantification kit (Roche) before sequencing on an Illumina MiSeq instrument according to the manufacturer’s protocol.

### Analysis of Cas-independent RNA editing

RNA off-target editing analysis was performed as previously described^15^. In brief, two 96-well plates of HEK293T cells were transfected in parallel with 250 ng of plasmid encoding editors and 83 ng of *EMX1* guide RNA plasmid in each well as described above. Forty-eight hours after transfection, one plate was used to evaluate on-target genomic DNA editing at the *EMX1* locus as described above. The other plate was used for RNA editing analysis as follows. Cells were lysed 48 hours after transfection using the RNeasy kit (Qiagen), following the manufacturerʼs instructions. In brief, culture medium was removed, and cells were washed with PBS before lysis in RLT Plus Buffer (Qiagen). Cells were transferred to a DNA eliminator column. Ethanol was added to the flowthrough, which was transferred to an RNeasy spin column. Samples were washed with RW1, and then on-column DNA digestion was carried out with RNase-Free DNase in RDD buffer (Qiagen). Samples were then washed with RW1 buffer, followed by a wash with RPE buffer. RNA was eluted in 45 µl of nuclease-free water, and 2 µl of RNaseOUT (Thermo Fisher Scientific) was added to each sample.

cDNA was generated with the SuperScript IV First-Strand Synthesis Kit (Thermo Fisher Scientific) according to the manufacturer’s instructions. The OligodT primer was annealed to RNA by heating at 65 °C and then cooling on ice for 1 minute. Reverse transcription reactions were prepared and added to the annealing mixtures. No-reverse transcriptase controls were included as a control for genomic DNA contamination. Reactions were incubated at 50 °C for 10 minutes and 80 °C for 10 minutes and then cooled on ice for 1 minute. The optional RNA degradation with RNaseH was carried out to increase the efficiency of cDNA amplification. The first PCR of targeted amplicon sequencing was conducted with 1 µl of each cDNA sample; the remaining sequencing protocol is identical to that described above for high-throughput, targeted genomic DNA sequencing. Primers used for first PCRs are listed in Supplementary Table 7.

### Library analysis of TadCBE editing outcomes

Base editor plasmids were constructed by cloning the new editor sequences into the previously described p2T-CMV-AID-BE4max-BlastR plasmid^11^. Undifferentiated 129P2/OlaHsd mESC (males) lines containing the previously reported 10,638-member ‘comprehensive 12kChar’ library^11^ were thawed and maintained on 15-cm plates as previously described^11^. To integrate the base editor plasmid into the cell lines containing the integrated library, cells were transfected with Tol2 transposase plasmid using Lipofectamine 3000 (Thermo Fisher Scientific) according to the manufacturer’s protocol and selected with blasticidin S (10 µg/ml) starting the day after transfection for 4 days before harvesting. We maintained an average coverage of 300× per library cassette throughout. We performed two biological replicates per base editor. Genomic DNA was collected from cells 4 days after antibiotic selection (5 days after base editor transfection). For library samples, 20 µg of genomic DNA was used for each sample for PCR1 amplification and sequencing, and we maintained an average sequencing depth of 2,800× per target. PCR1 was performed to amplify the endogenous locus or library cassette using the primers specified in Supplementary Table 8. PCR2 was performed to add full-length Illumina sequencing adapters using the NEBNext Index Primer sets 1 and 2 (New England Biolabs). All PCR reactions were performed using NEBNext Ultra II Q5 Master Mix. Extension time for all PCR reactions was extended to 2 minutes per cycle to prevent PCR amplification bias. Samples were quantified by TapeStation (Agilent), pooled and quantified using a KAPA Library Quantification Kit (Roche) before sequencing. Library sequencing was performed on an Illumina NextSeq with paired-end reads (94 forward and 56 reverse).

Data processing and analysis were performed with Python 3.9. Library samples were demultiplexed for each editor/replicate with bcl2fastq2 (Illumina), with all lanes merged. To assign each paired-end read to a library member, we discarded any reads below Q28 in the target sites and sgRNA spacer sequence. We then nominated candidate target sites with locality-sensitive hashing using tiled 6-mers across the target site. We filtered out any reads with where the sgRNA spacer sequenced did not match a candidate target site. Finally, we genotyped each target site by performing Needleman–Wunsch alignment (scoring parameters: match = 1, mismatch = −1, gap open = −5, gap extend = 0 and start gap = 0).

Before further data analysis, we considered two sources of noise in our sequencing data. First, the expansion of the mESC cell line harboring the genomically integrated libraries could lead to the stochastic amplification of errors present in the initial cell library after selection (so-called ‘batch effects’). Second, next-generation sequencing on Illumina systems can occasionally misassign reads. To minimize both error sources, we only considered A-to-G, C-to-T, C-to-G and C-to-A mutations within the −9 through 20 base editing window, in accordance with our previous work with this cell library^11^.

We searched for potential batch effects in our mutation data by comparing the frequencies of each mutation at each position within our window with one-way ANOVA. We were encouraged to see that there were no batch-specific mutations within our window that were outside of the range of statistical noise (at a Bonferroni-corrected significance level of 0.005). This outcome agrees with our previous work with this cell library^11^, in which we also did not observe any significant batch effects within the base editing window.

Finally, we filtered out reads that were likely due to Illumina sequencing noise. We considered that mutations due to rare base editing outcomes would likely still be present across both replicates of our library, even if their presence in each replicate were below the threshold that would be traditionally considered noise. Therefore, we computed the likelihood that each mutation at each position would be observed in the corresponding number of reads in both replicates based on a Bernoulli distribution with a rate parameter of 10^−3^ (Q30). We kept all mutations that were less than 5% likely to be due to sequencing noise.

For position-wise editing efficiency analyses, we combined the number of reads containing each mutation between replicates and divided by the total number of reads observed for each given library member. We chose to combine replicates in this way (rather than, for example, averaging the frequencies for each replicate) because it is the maximum-likelihood estimate of the rate parameter of a hypothetical Bernoulli distribution that describes the base editing efficiency at a given position.

In our analyses, we defined the ‘average editing efficiency’ across our library as the average fraction of (noise-filtered, batch-combined) reads containing our specified editing outcome. To define selectivity for cytosine over adenine deamination, we first computed the average cytosine editing efficiency and the average adenine editing efficiency at positions within the ≥30% editing window across all members of our library. We then computed the geometric mean of the selectivity at each position to obtain a conservative estimate of the ‘overall’ selectivity of each editor. Because a given position can only contain either a cytosine or an adenine, the true selectivity in a given scenario will depend on the positions of the respective bases.

To generate sequence motifs of the context preferences of our editors, we first transformed our editing fraction with a stabilized logit function: ${{{\mathrm{log}}}}({\frac{{x + {\it{\epsilon }}}}{{1 + {\it{\epsilon }} - x}}})$, where ${\it{\epsilon }}$ is a small constant that stabilizes the function behavior for inputs close to 0 or 1. For our purposes, we chose to use ${\it{\epsilon }} = 0.001$, as this is a conservative estimate of the noise due to Illumina sequencing. We then performed a random train/test split (80:20, respectively) and trained a ridge regression with *α* = 10^−5^ to generate weights that were visualized in a sequence logo.

To evaluate the fold changes in C•G-to-T•A and A•T-to-C•G conversion efficiency upon inclusion of the V106W mutation in TadCBEd, we performed total least squares (TLS) regression on the (noise-filtered, batch-corrected) efficiency of installing the specified edit with each editor. We chose to perform TLS rather than ordinary least squares, because we were computing a relationship between two measured variables (as opposed to the dependence of one variable on another, independent variable). We defined the average fold decrease as the reciprocal of the regression weight (where *x* is TadCBEd and *y* is TadCBEd-V106W).

### Analysis of HTS data for DNA sequencing and targeted amplicon sequencing

Individual high-throughput sequencing datasets were demultiplexed using the MiSeq Reporter (Illumina). Subsequent demultiplexed sequencing reads were analyzed using CRISPResso2 (ref. ^78^) and analyzed in Microsoft Excel (version 16.64) as described previously^15^.

### Reporting summary

Further information on research design is available in the Nature Research Reporting Summary linked to this article.

## Online content

Any methods, additional references, Nature Research reporting summaries, source data, extended data, supplementary information, acknowledgements, peer review information; details of author contributions and competing interests; and statements of data and code availability are available at 10.1038/s41587-022-01533-6.

## Supplementary information

Supplementary Information Supplementary Figs. 1–42, Supplementary Tables 1–8 and Supplementary Notes 1 and 2

Reporting Summary

## Data Availability

High-throughput DNA sequencing FASTQ files are available from the National Center of Biotechnologyʼs Information Sequence Read Archive under BioProject PRJNA848090 (ref. ^79^). Amino acid sequences of deaminases in this study are provided in the Supplementary Information as Supplementary Sequences 1 and 2. CSV files containing processed data for library experiments have been uploaded to Figshare and assigned (10.6084/m9.figshare.21210845 (ref. ^80^)). Processed data from Figs. 1–6 are included as Source Data. The previously published structure of ABE8e that was used for mutational analysis is available in the Protein Data Bank (6VPC). Other data files, including phage titers from evolution and mammalian cell data analysis (PRISM and Excel files), are available from the corresponding authors upon reasonable request. Plasmids encoding TadCBEs are available at Addgene. Source data are provided with this paper.

## Source data

Source Data Fig. 1 Source data for Fig. 1
Source Data Fig. 2 Source data for Fig. 2
Source Data Fig. 3 Source data for Fig. 3
Source Data Fig. 4 Source data for Fig. 4
Source Data Fig. 5 Source data for Fig. 5
Source Data Fig. 6 Source data for Fig. 6

## Code availability

All code used for processing library data is available on GitHub at https://github.com/alvin-hsu/BELib_Processing (ref. ^81^). All other scripts used for analysis in this work were previously reported by Doman et al.^15^. Source data are provided with this paper.
