# Supplementary material for: Evolution of an adenine base editor into a small, efficient cytosine base editor with low off-target activity
Source: Nat Biotechnol. 2022 Nov 10;41(5):673–85. doi: 10.1038/s41587-022-01533-6 (PMC10188366; doi:10.1038/s41587-022-01533-6)
Supplement: Supplementary file 1 — Supplementary Figs. 1–42, Supplementary Tables 1–8 and Supplementary Notes 1 and 2 [file 41587_2022_1533_MOESM1_ESM.pdf]

nature biotechnology

Article

https://doi.org/10.1038/s41587-022-01533-6

Evolution of an adenine base editor into a small, efficient cytosine base editor with low off-target activity

In the format provided by the authors and unedited

Supplementary Figures: Table of Contents

1. Basis of deamination selectivity selection in PACE and PANCE circuits.
2. PANCE titers and evolved TadA-CD genotypes.
3. PACE titers and evolved TadA-CD genotypes.
4. AlphaFold model of TadA-CDa.
5. Testing individual mutations in TadCBEs.
6. Reversion analysis of TadCBEs.
7. Indels and C•G-to-G•C editing by SpCas9 variants at nine genomic target sites.
8. V106W proximity to evolved TadA-CD mutations.
9. Base editing by V106W variants at six genomic target sites.
10. Indels and C•G-to-G•C editing by V106W variants at six genomic target sites.
11. Base editing, indel formation, and C•G-to-G•C editing by V106W variants at three additional genomic target sites.
12. Base editing activity windows of CBEs across nine genomic target sites.
13. On-target editing of *EMX1* in the Cas-independent R-loop editing experiment.
14. Cas-independent off-target C•G-to-T•A editing at individual sites within six orthogonal R-loops generated by SaCas9.
15. Cas-independent off-target C•G-to-T•A editing by TadCBEe V106W at individual sites within six orthogonal R-loops generated by SaCas9.
16. Cas-independent off-target DNA editing at six genomic SaCas9 R-loops.
17. Cas-independent off-target DNA editing at six genomic SaCas9 R-loops.
18. Cas-independent off-target RNA editing by TadCBEe V106W of all cytosines and adenines examined across three transcripts.
19. On-target editing of *EMX1* in the RNA off-target editing experiment.
20. Cas-dependent off-target editing of known off-target sites for *HEK3*.
21. Cas-dependent off-target editing of known off-target sites for *HEK4*.
22. Cas-dependent off-target editing of known off-target sites for *EMX1*.
23. On-target editing of *EMX1.*
24. On-target and off-target editing of *EMX1* by TadCBEe V106W.
25. Cas-dependent off-target editing of known off-target sites for *BCL11A*.
26. Schematic of the mESC library experiment.
27. Correlation between replicates in the mESC library experiment.
28. Editing windows of TadCBE variants in the mESC library editing experiment.
29. Effect of V106W on peak editing in the mESC library experiment.
30. Sequence motifs for context preferences of TadCBEs.
31. Characterization of evolved deaminases with evolved eNme2-C Cas9 domains.
32. Indels and C•G-to-G•C editing by eNme2-C Cas9 variants at six genomic target sites.
33. Characterization of evolved deaminases with SaCas9 domains.
34. Indels and C•G-to-G•C editing by SaCas9 Cas9 variants at six genomic target sites.
35. Characterization of TadDE with SpCas9 in mammalian cells.

36. Indels and C•G-to-G•C editing by TadDE with SpCas9 at nine genomic target sites.
37. C•G-to-G•C editing and indels for T-cell experiments targeting *CXCR4* and *CCR5*.
38. Cas-dependent off-target editing in T-cell experiments targeting *CXCR4* and *CCR5*.
39. On-target editing of V106W variants for T-cell experiments targeting *CXCR4* and *CCR5*.
40. C•G-to-G•C editing and indels for T-cell experiments targeting *CXCR4* and *CCR5* with TadCBE V106W variants.
41. Cas-dependent off-target editing in T-cell experiments targeting *CXCR4* and *CCR5* with TadCBE V106W variants.
42. C•G-to-G•C editing, indels, and Cas-dependent off-target editing for editing of *BCL11A* in hematopoietic stem and progenitor cells.

Supplementary Tables

1. Selectivity of TadCBEs and TadDE calculated from the mESC library experiment.
2. Plasmids and selection phage (SP) used in this work.
3. Promoter and RBS sequences for plasmids and phage used in evolution.
4. Target protospacers and amplicons used in this study with corresponding primers for genomic DNA amplification.
5. Primers for generating base editor amplicons for IVT.
6. Chemically synthesized guide RNAs used for T-cell and HSPC experiments.
7. cDNA amplicon sequences and primers for RNA off-target analysis.
8. Primer sequences for library analysis.

Supplementary Notes

1. Evolved TadA-CD amino acid sequences.
2. Sequences of Cas9 domains used in this study.

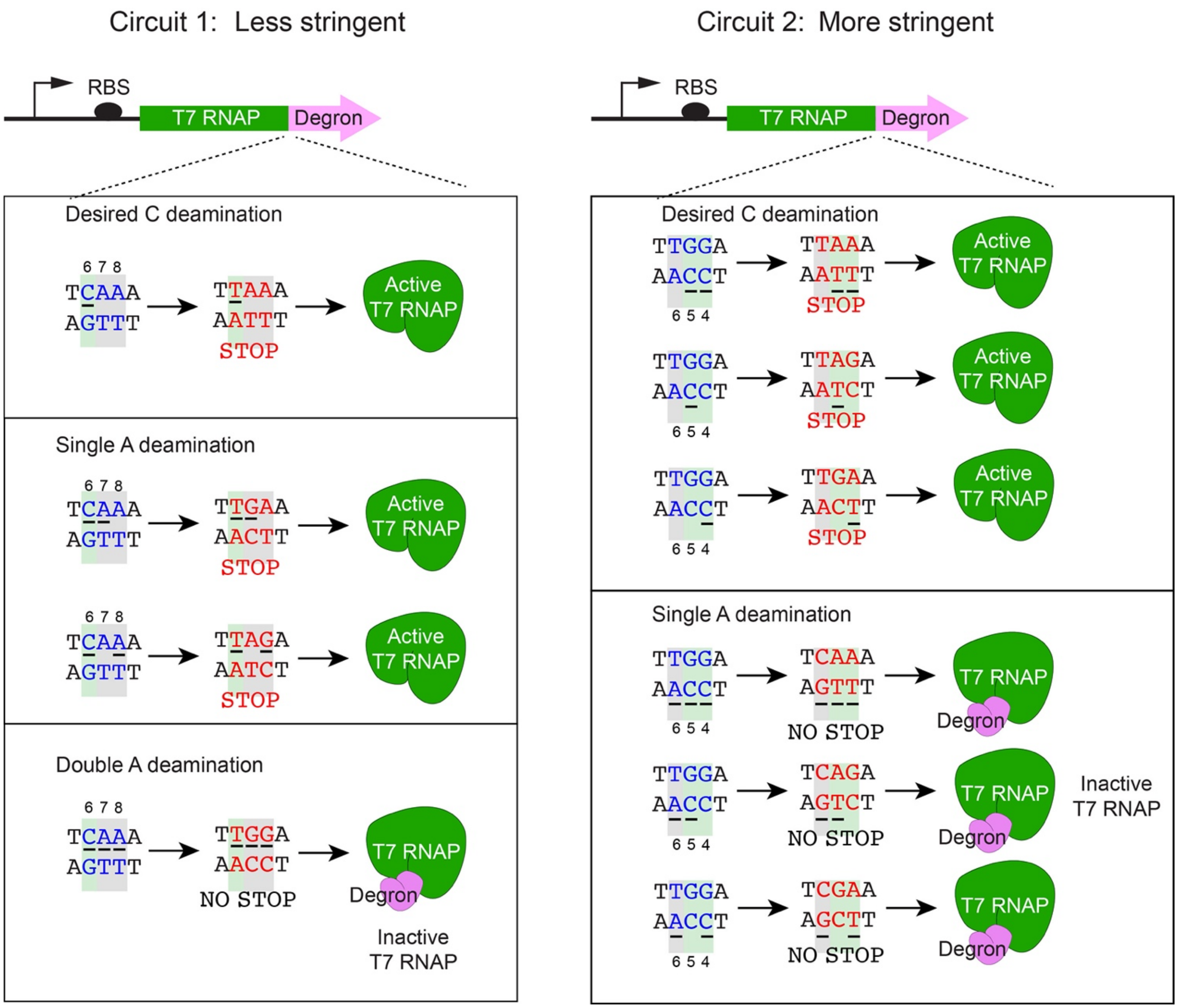

Supplementary Figure 1. Basis of deamination selectivity selection in PACE and PANCE circuits. In Circuit 1, stop codon formation is only impeded if the base editor deaminates *both* A_7 and A_8. Circuit 1 is thus tolerant to modest levels of A deamination. In Circuit 2, deamination of a single adenine A_6 will prevent stop codon formation and impede circuit activation and phage propagation. Circuit 2 is thus more stringent for selecting against deoxyadenosine deamination.

a

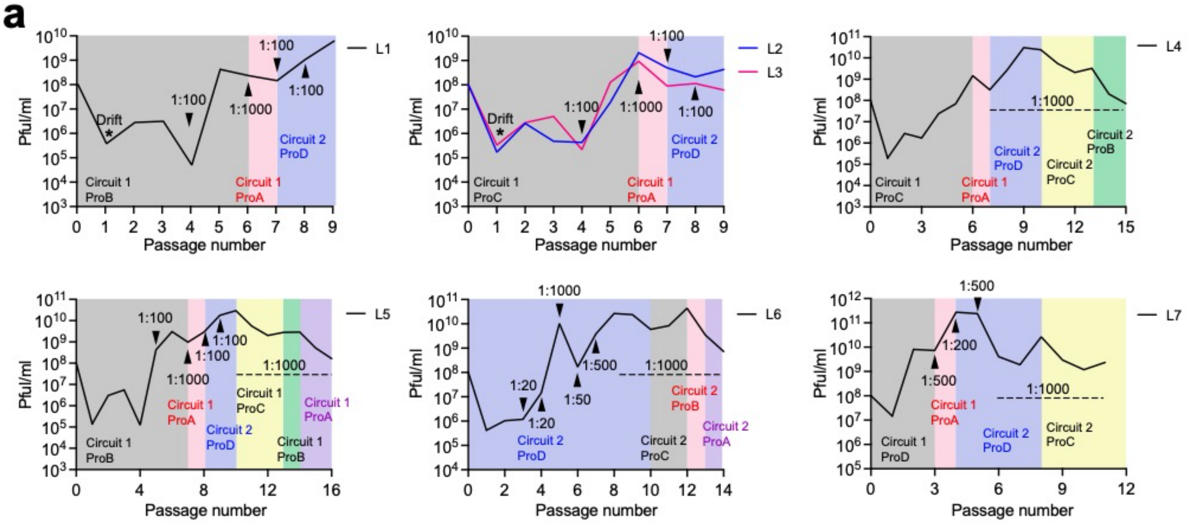

b

TadA-8e	R26	E27	V28	V29	A48	M61	G66	H95	S109	R129	Q154	A158
L1-1												
L1-2		K										
L1-3		K										
L1-4		A	G									S
L2-1		A	G									
L2-2			G									
L2-3			G									
L2-4			G									
L3-1		K	A									
L3-2		A	G									
L3-3		A	G						L			
L3-4			G	G								
L4-1		K	A			I						
L4-2		K	A			I						
L4-3		K	A									
L4-4		K	A									
L5-1		K	A									
L5-2		K	A							L		
L5-3		K	A									
L5-4		K	A									
L6-1		K	A									
L6-2		K	A								R	
L6-3		K	A									
L6-4		K	A								R	
L7-1	G		A		R		V	N				
L7-2	G		A		R		V	N				
L7-3	G		A		R		V	N				

Supplementary Figure 2. PANCE titers and evolved TadA-CD genotypes. (a) Phage titers during PANCE for Lagoons 1–7. Stringency was modulated by increasing the promoter strength from ProD (strongest, least stringent) to ProA (weakest, most stringent), increasing the dilution factor, and by switching from Circuit 1 to Circuit 2. Lagoons 1–6 were inoculated with phage encoding TadA8e-NpuN, while Lagoon 7 was inoculated with phage encoding TadA8e A48R-NpuN. **(b)** Genotypes from various PANCE lagoons (L1–L7) after PANCE.

a

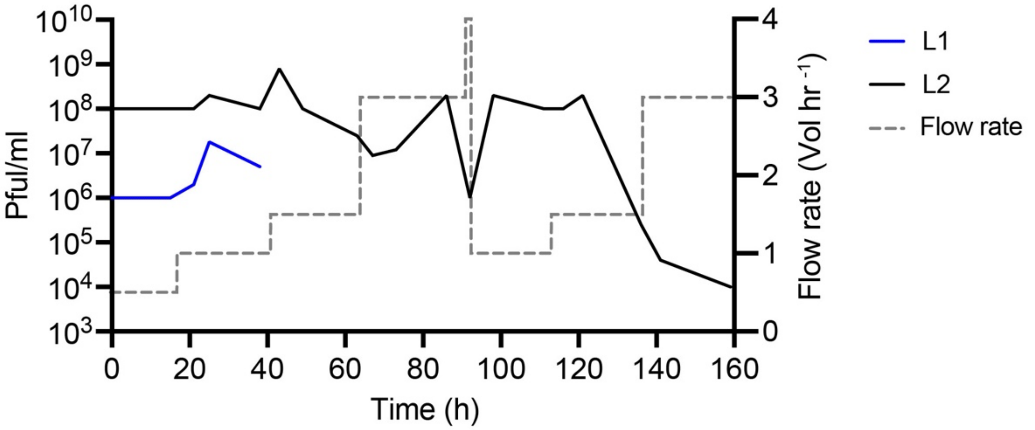

b

t = 43 h	E27	V28	M61	Q154
L1-1	K	A		R
L1-2	K	A	I	
L1-3	K	A	I	R
L1-4	K	A		R

c

Time (h)	R26	E27	V28	G50	Y73	I76	H96	S97	G100	M151	Q154	A158	S165	T180	T183	S186
43			G													
43			G													
43			G									S				
67		A	G		H	F		A				S				
67	G	A	G			F	N					S				
73	G	A	G			F	N					S				
73	G		G			F	N					S				
92	G		G			F	N					S	F	A	P	
98	G		G	S							R	S				I
98	G	A	G			F	N					S				
111	G	A	G	V						I	R	S				
111	G		G			F	N					S				
116	G		G			F	N					S				
116	G		G			F	N					S				
121	G		G			F	N					S				
121	G	A	G			F	N					S				
121	G		G			F	N				R	S				
141	G		G			F	N					S				
141	G		G			F	N					S				
159	G		G			F	N		S			S				
159	G	A	G			F	N					S				
159	G	A	G			F	N					S				N

Supplementary Figure 3. PACE titers and evolved TadA-CD genotypes. (a) Phage titers and lagoon flow rate during PACE. Lagoon 1 showed activity-independent propagation in S2060 cells after t=43 h, signifying phage that evolved selection-independent replication, and was not continued. **(b)** Genotypes of evolved TadA* variants from lagoon 1 at t=43 h, before the appearance of selection-independent propagation. **(c)** Genotypes of evolved TadA* variants from lagoon 2 at various time points.

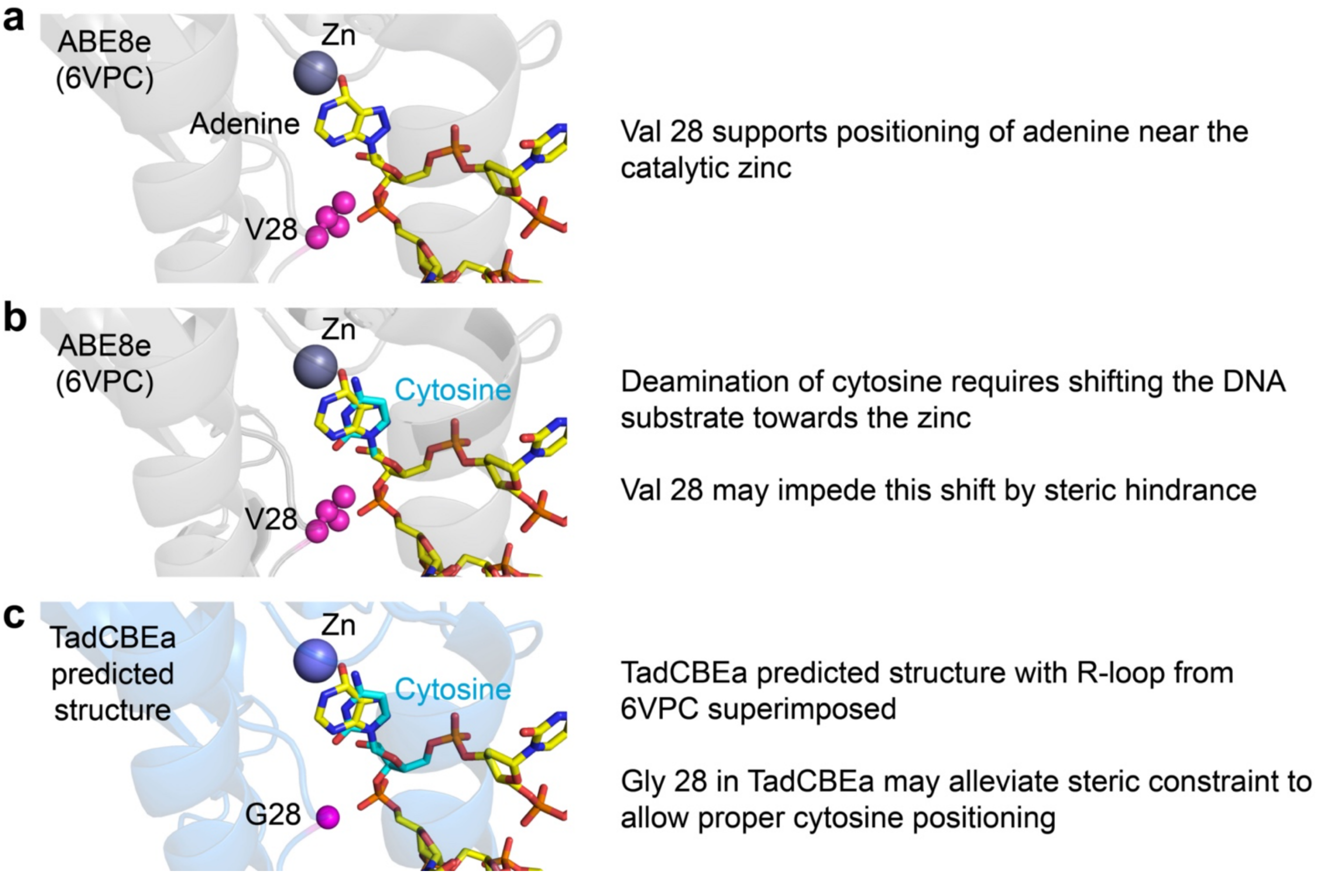

Supplementary Figure 4. AlphaFold model of TadA-CDa. (a) The cryo-EM structure of ABE8e (PDB ID 6VPC)[1] is shown bound to DNA containing the 8-azanebularine (8Az) substrate mimic of adenosine. Val 28 (magenta) supports proper positioning of the adenine substrate relative to the catalytic zinc. **(b)** 8Az was replaced with cytidine using the “Swapna” function in the Chimera software[2]. In the resulting model, C4 of cytosine, which is targeted for nucleophilic attack during deamination, is ~1 Å away from the target carbon of 8Az, and thus may require shifting of the DNA substrate for productive catalysis. Val 28 may impede this shift of the DNA substrate deeper into the TadA-8e pocket. **(c)** AlphaFold[3] was used to generate a model of evolved TadA-CDa. The ABE8e structure was superimposed to generate a model with the DNA substrate R-loop from 6VPC. The evolved enzyme is not predicted to adopt any apparent differences in secondary structure compared to TadA8e. Evolved replacement of Val 28 in TadA-8e to the smaller Ala or Gly residues found in TadA-CDs may alleviate steric constraints that are predicted to impede productive positioning of the target C4 in cytosine relative to the catalytic zinc ion.

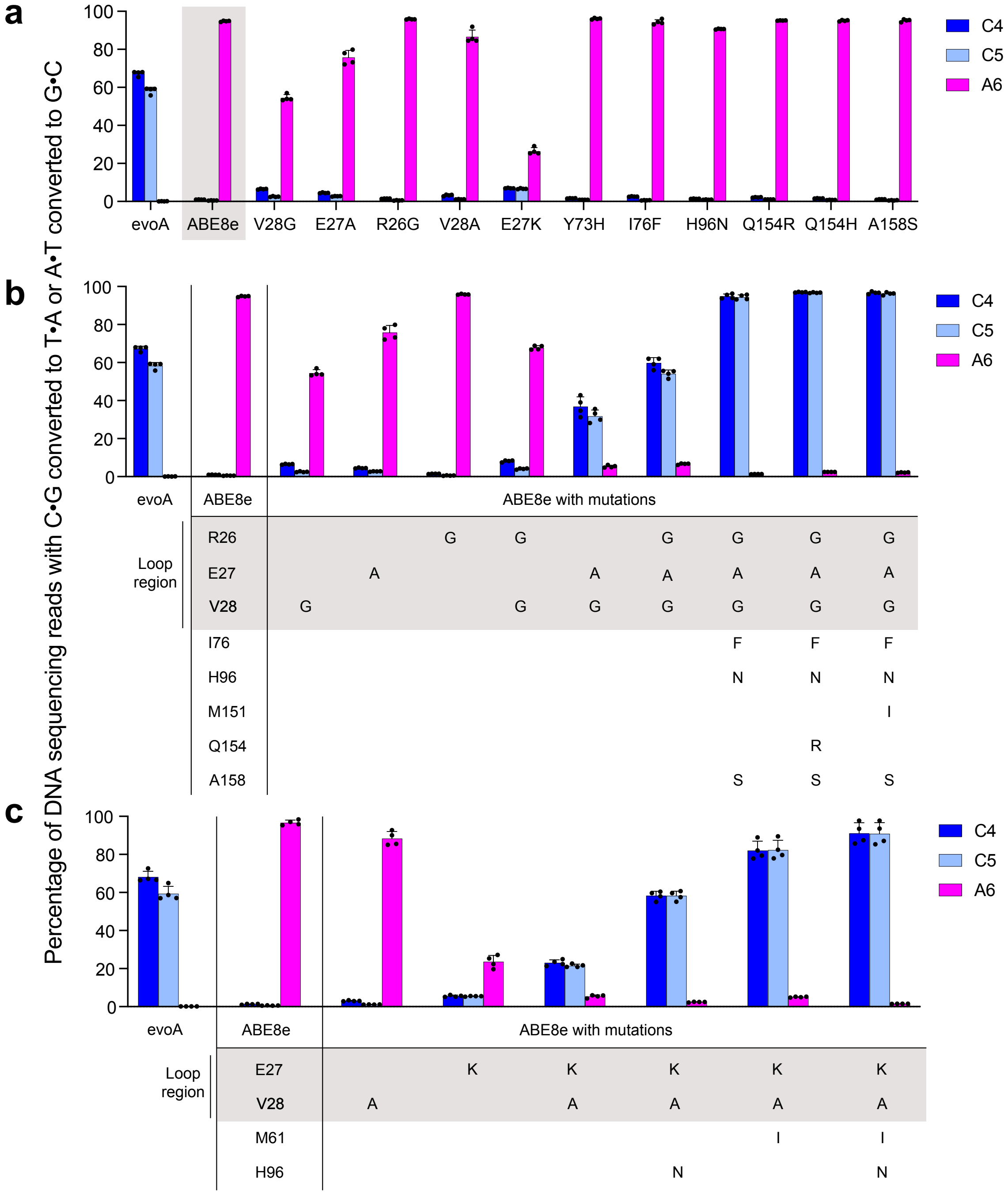

Supplementary Figure 5. Testing individual mutations in TadCBEs. Base editing in *E. coli* of a protospacer matching the selection circuit target site. Cells are co-transformed with a target plasmid and a base editor plasmid. Base editor expression is induced with arabinose. After 16 hours, cells are harvested, and the target plasmid is analyzed by high-throughput sequencing. **(a)** Addition of individual mutations identified through evolution to ABE8e is insufficient for generating a CBE. **(b)** Analysis of mutations in TadCBEa-c and TadCBEe. Mutations in the loop region of ABE8e imparts selectivity for cytidine deamination, while auxiliary mutations boost activity. **(c)** Analysis of mutations in TadCBEd. C•G-to-T•A edits are shown in blue. A•T-to-G•C edits are shown in magenta. Dots represent individual biological replicates and bars represent mean±s.d. from four independent biological replicates.

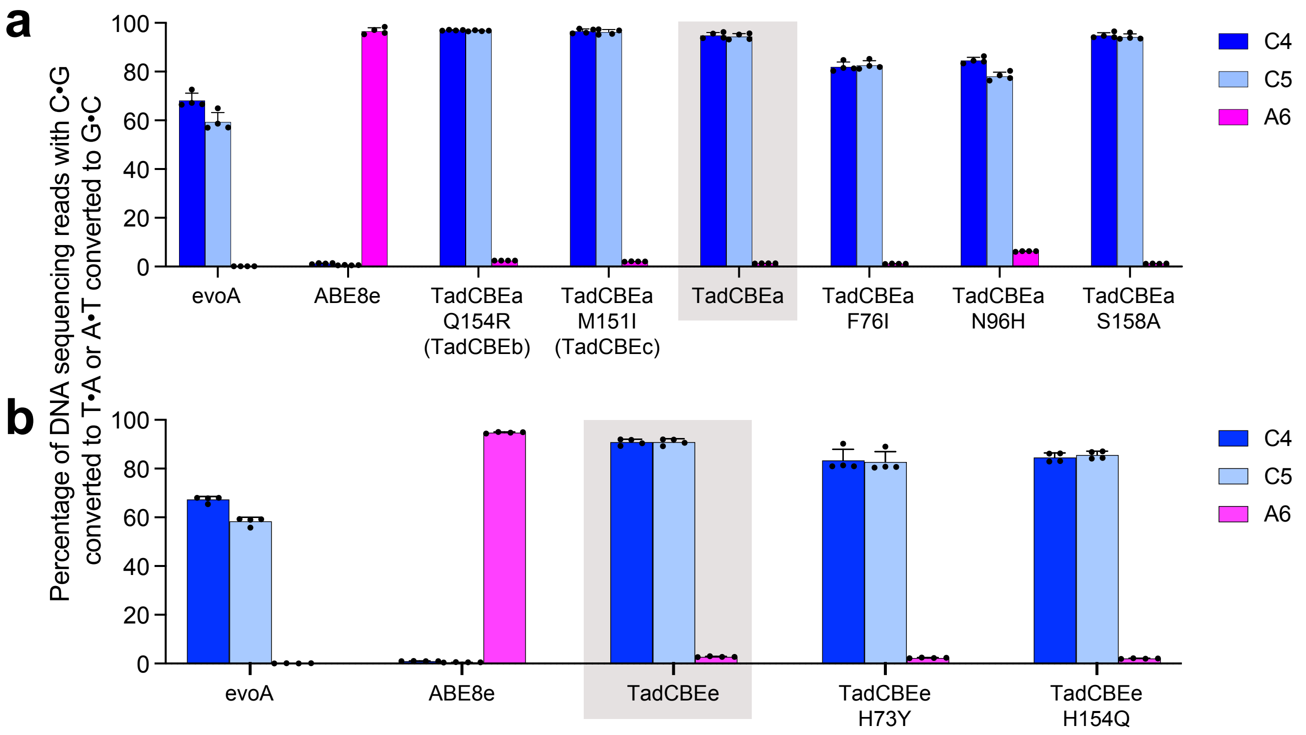

Supplementary Figure 6. Reversion analysis of TadCBEs. Base editing in *E. coli* of a protospacer matching the selection circuit target site. Cells are co-transformed with a target plasmid and a base editor plasmid. Base editor expression is induced with arabinose. After 16 hours, cells are harvested, and the target plasmid is analyzed by high-throughput sequencing. **(a)** Mutations shown are relative to TadCBEa (grey box). **(b)** Mutations shown are relative to TadCBEe (grey box). C•G-to-T•A edits are shown in blue. A•T-to-G•C edits are shown in magenta. Dots represent individual biological replicates and bars represent mean±s.d. from four independent biological replicates.

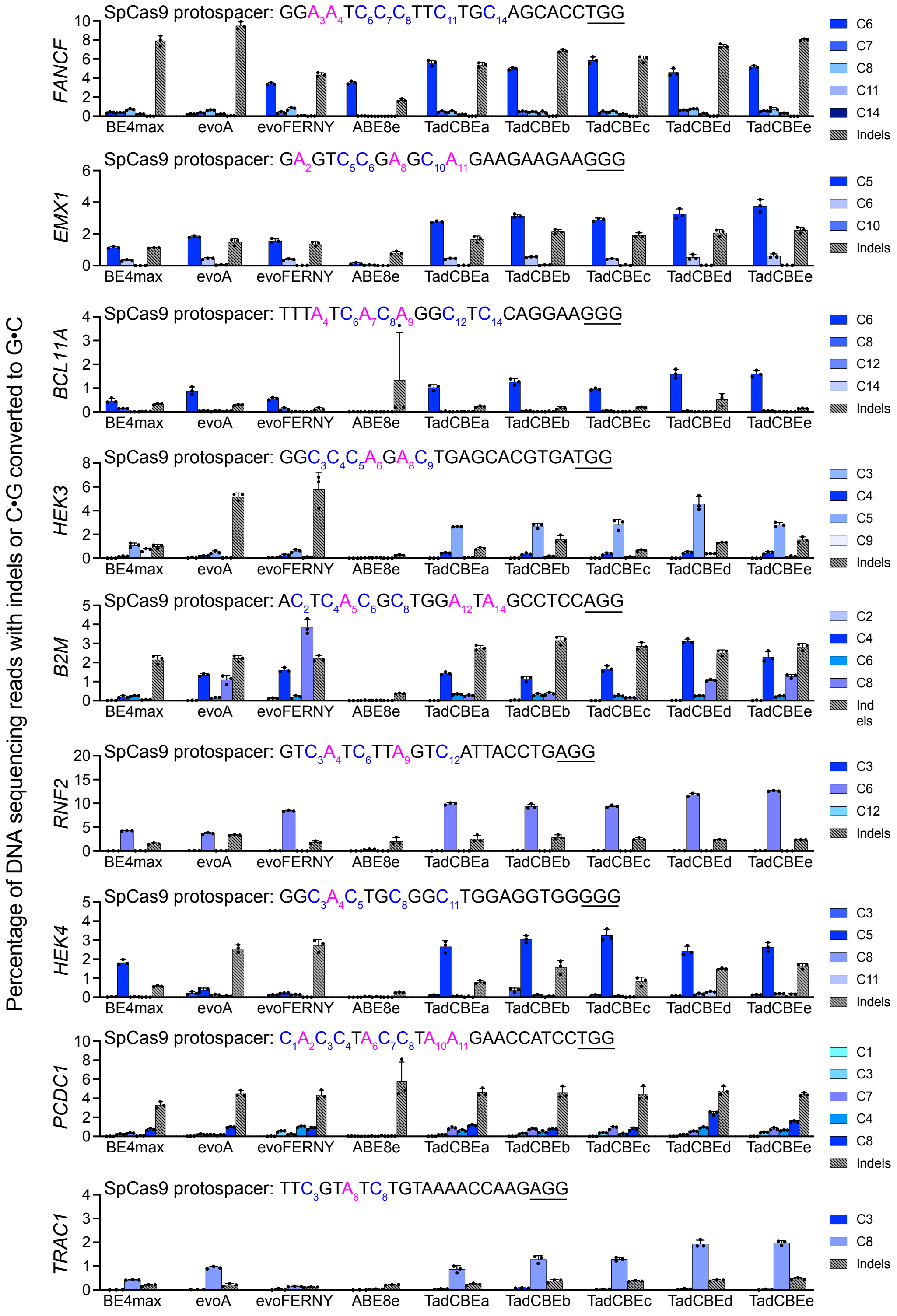

Supplementary Figure 7. Indels and C•G-to-G•C editing by SpCas9 variants at nine genomic target sites. The specified base editors using SpCas9 nickase domains in the BE4max architecture or ABE8e with 2xUGI were transfected into HEK293T cells along with each of nine guide RNAs targeting the protospacers shown in each graph. C•G-to-G•C base editing is shown in shades of blue. Indels are shown in grey. Dots represent individual values and bars represent mean±s.d. of three independent biological replicates. The corresponding on-target editing data can be found in Figure 3.

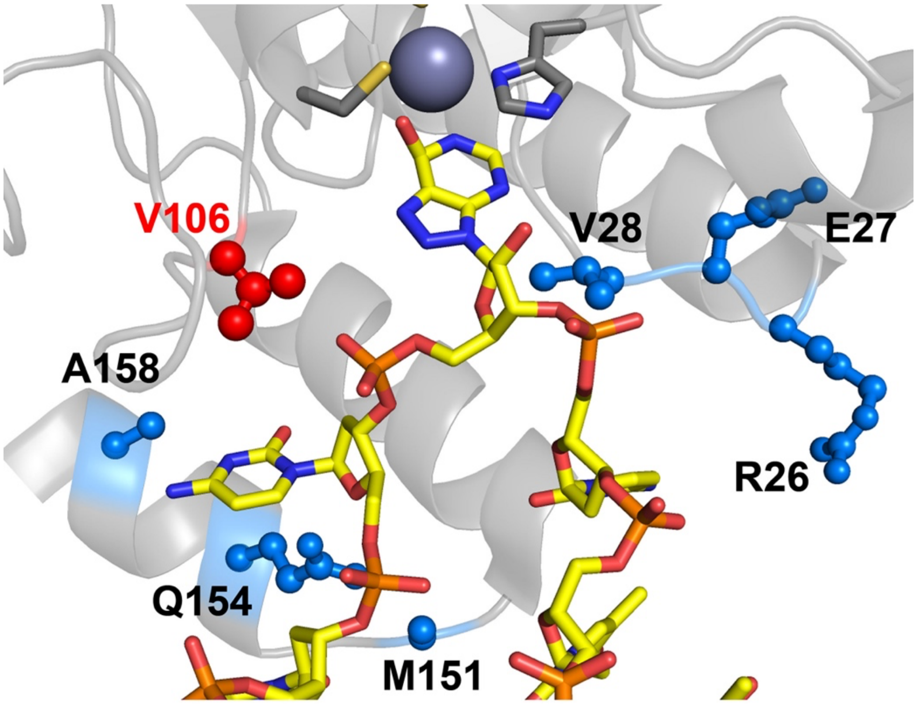

Supplementary Figure 8. V106W proximity to TadA-CD mutations. Mutations generated during the evolution of TadA-CDs are shown in blue. Residue V106 is shown in red. The addition of V106W to TadA-7.10, TadA-8e, and TadA-8.17 reduces off-target editing activity[4–6]. The addition of V106W to TadCBEa-e increases selectivity for deaminating deoxycytidine over deoxyadenosine and also reduces off-target editing activity.

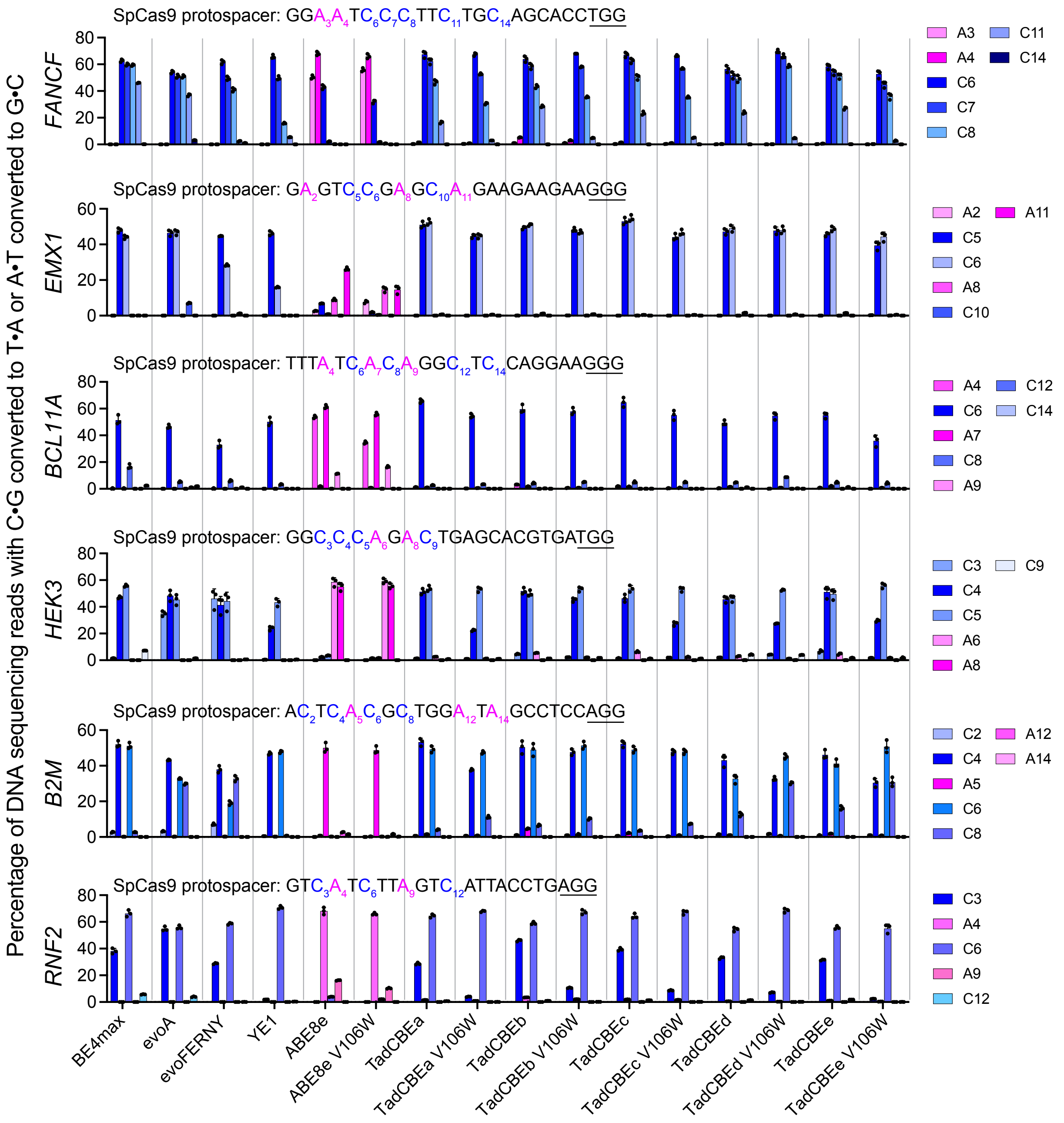

Supplementary Figure 9. Base editing by V106W variants at six genomic target sites. The specified base editors using SpCas9 nickase domains in the BE4max architecture or ABE8e with 2xUGI were transfected into HEK293T cells along with each of six guide RNAs targeting the protospacers shown in each graph. Target cytosines are blue, target adenines are magenta, and PAM sequences are underlined. C•G-to-T•A base editing is shown in shades of blue. A•T-to-G•C base editing is shown in shades of magenta. Dots represent individual values and bars represent mean±s.d. of three independent biological replicates.

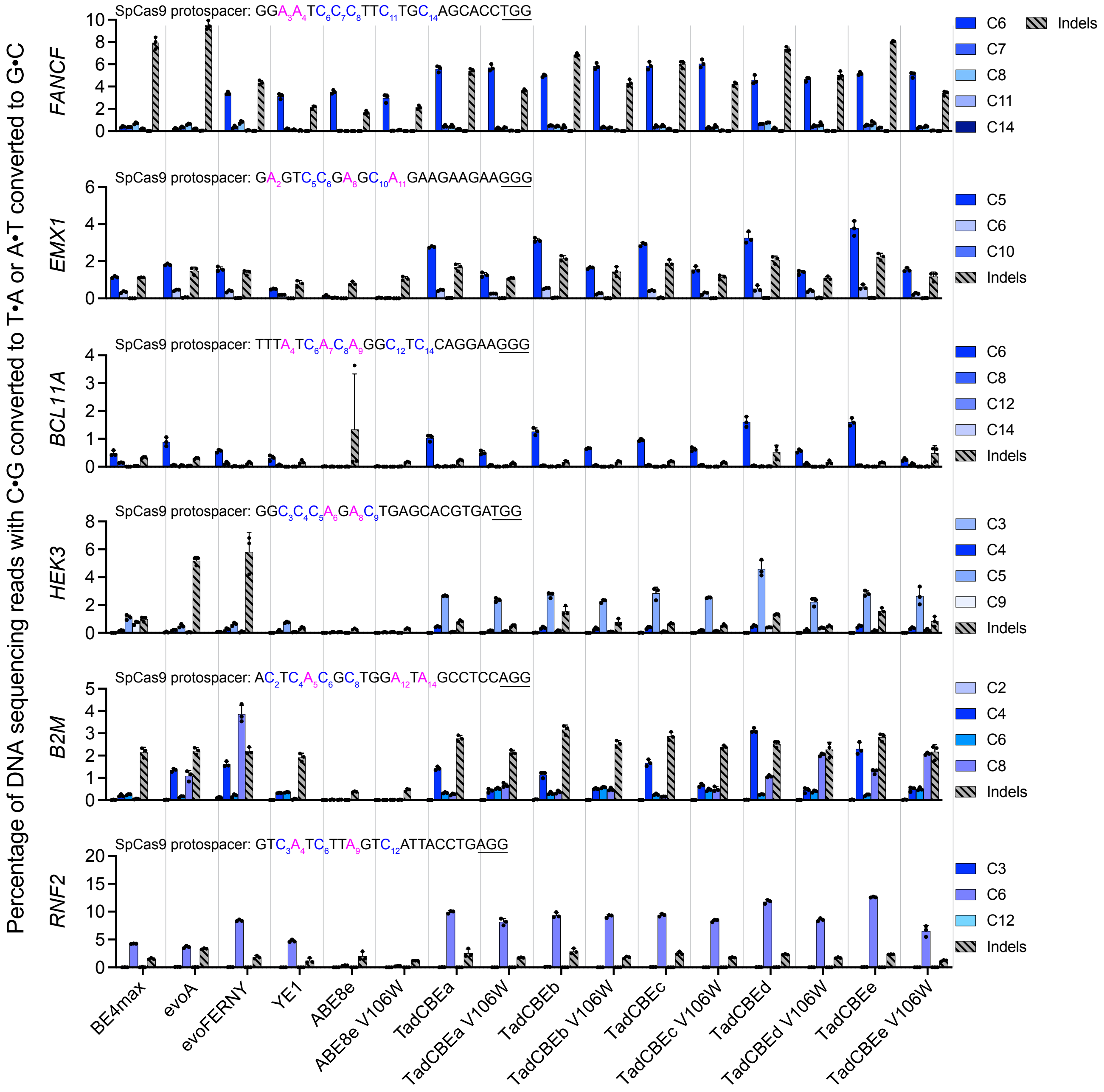

Supplementary Figure 10. Indels and C•G-to-G•C editing by V106W variants at six genomic target sites. The specified base editors using SpCas9 nickase domains in the BE4max architecture or ABE8e with 2xUGI were transfected into HEK293T cells along with each of six guide RNAs targeting the protospacers shown in each graph. C•G-to-G•C base editing is shown in shades of blue. Indels are shown in grey. Dots represent individual values and bars represent mean±s.d. of three independent biological replicates.

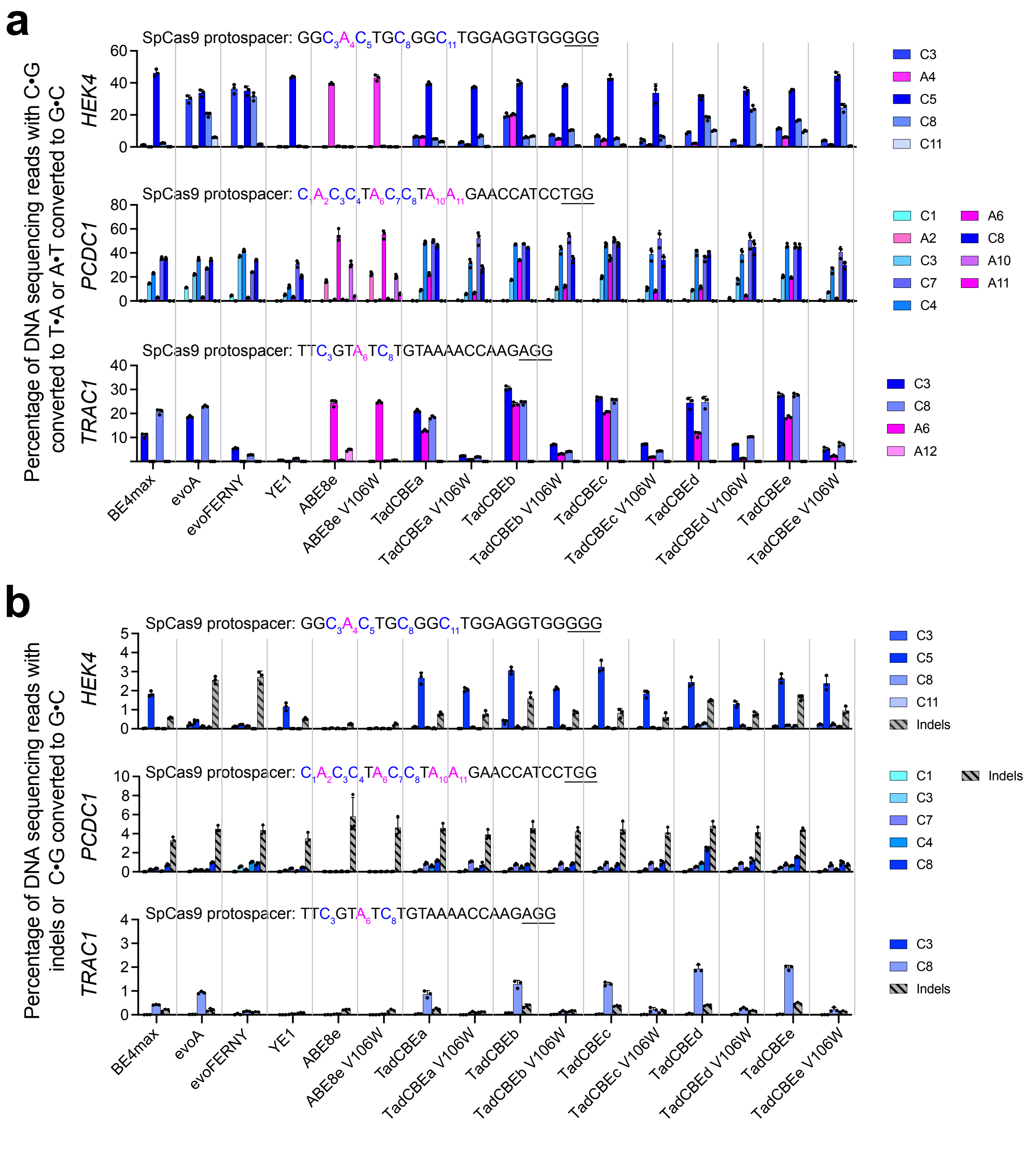

Supplementary Figure 11. Base editing, indel formation, and C•G-to-G•C editing by V106W variants at three aditional genomic target sites. The specified base editors using SpCas9 nickase domains in the BE4max architecture or ABE8e with 2xUGI were transfected into HEK293T cells along with each of three guide RNAs targeting the protospacers shown in each graph. **(a)** Target cytosines are blue, target adenines are magenta, and PAM sequences are underlined. C•G-to-T•A base editing is shown in shades of blue. A•T-to-G•C base editing is shown in shades of magenta. **(b)** C•G-to-G•C base editing is shown in shades of blue. Indels are shown in grey. Dots represent individual values and bars represent mean±s.d. of three independent biological replicates.

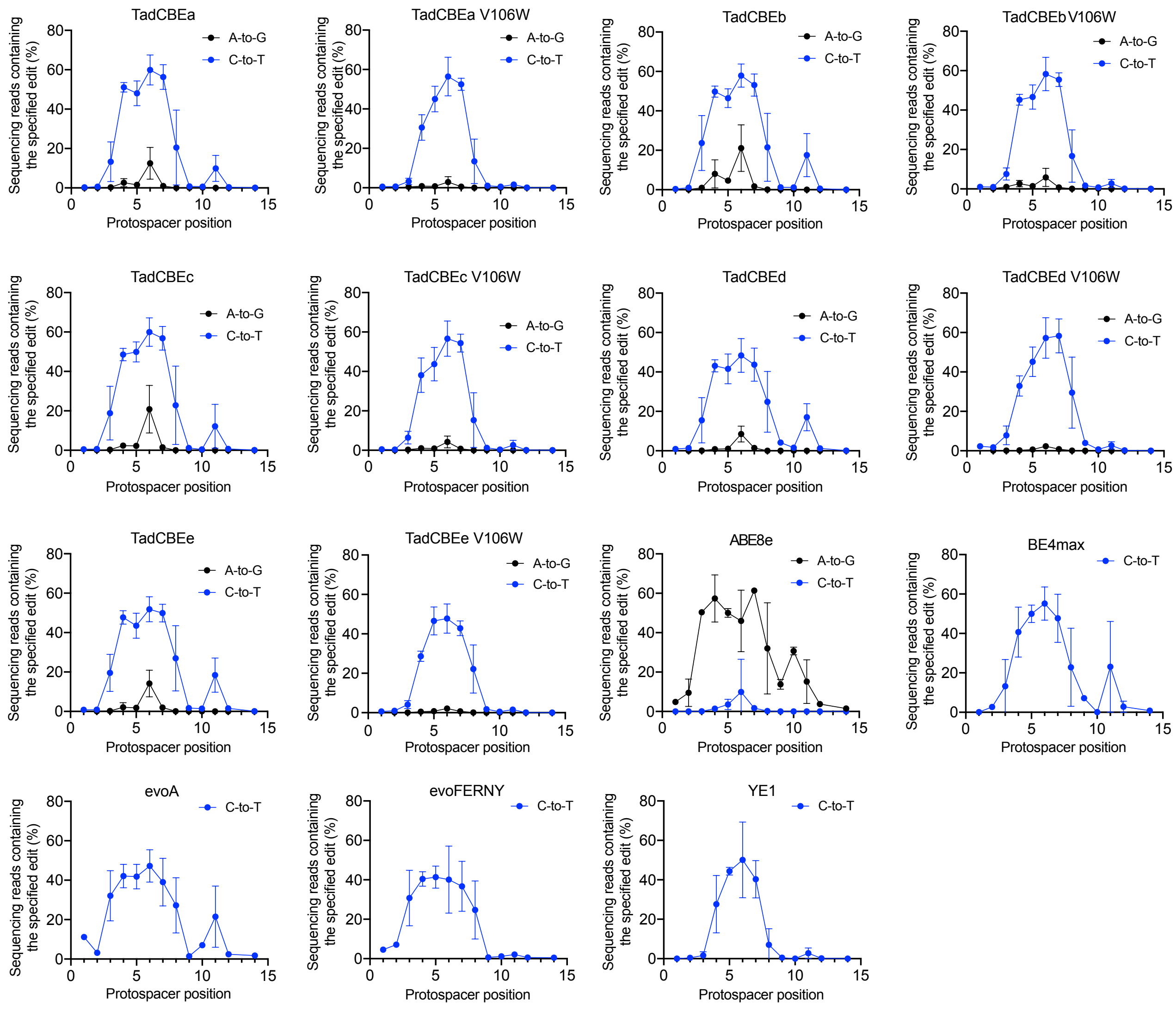

Supplementary Figure 12. Base editing activity windows of CBEs across nine genomic target sites. Mean editing at various cytosines or adenines across nine tested sites were grouped by the position of the cytosine within the protospacer (counting the PAM as positions 21-23) and averaged. Dots represent mean±s.d. for editing across all nine sites containing the specified base at the indicated position within the protospacer (n = 3 biological replicates for each of the nine sites). Individual data points for the nine sites used for this analysis are in Fig. 3 and Supplementary Figs. 9 and 11.

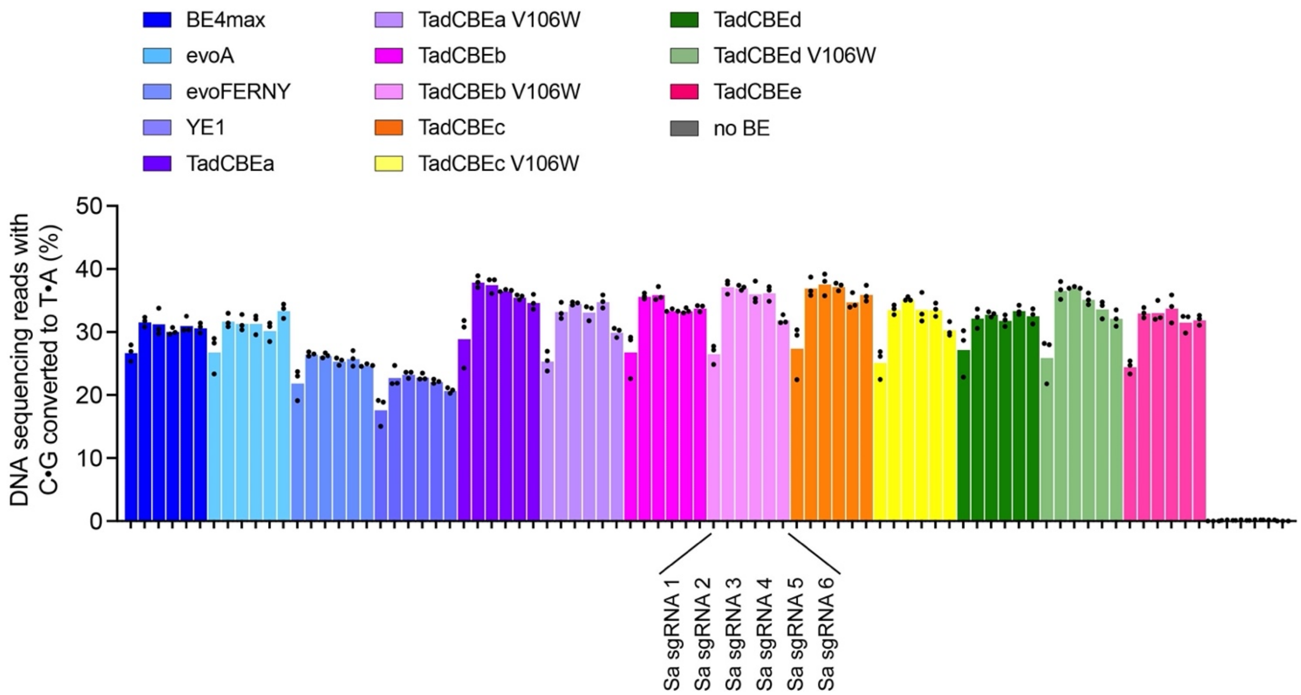

Supplementary Figure 13. On-target editing of EMX1 in the Cas-independent R-loop editing experiment. The specified base editors using SpCas9 nickase domains in the BE4max architecture or ABE8e with 2xUGI were transfected into HEK293T cells along with a SpCas9 guide RNA targeting *EMX1* as well as the indicated SaCas9 sgRNA. The average on-target C•G-to-T•A base editing across C_5 and C_6 in *EMX1* is shown for the indicated base editor. Dots represent individual values and bars represent mean±s.d. of three independent biological replicates. The corresponding Cas-dependent off-target data are shown in Figure 4c and Supplementary Figs. 14–15.

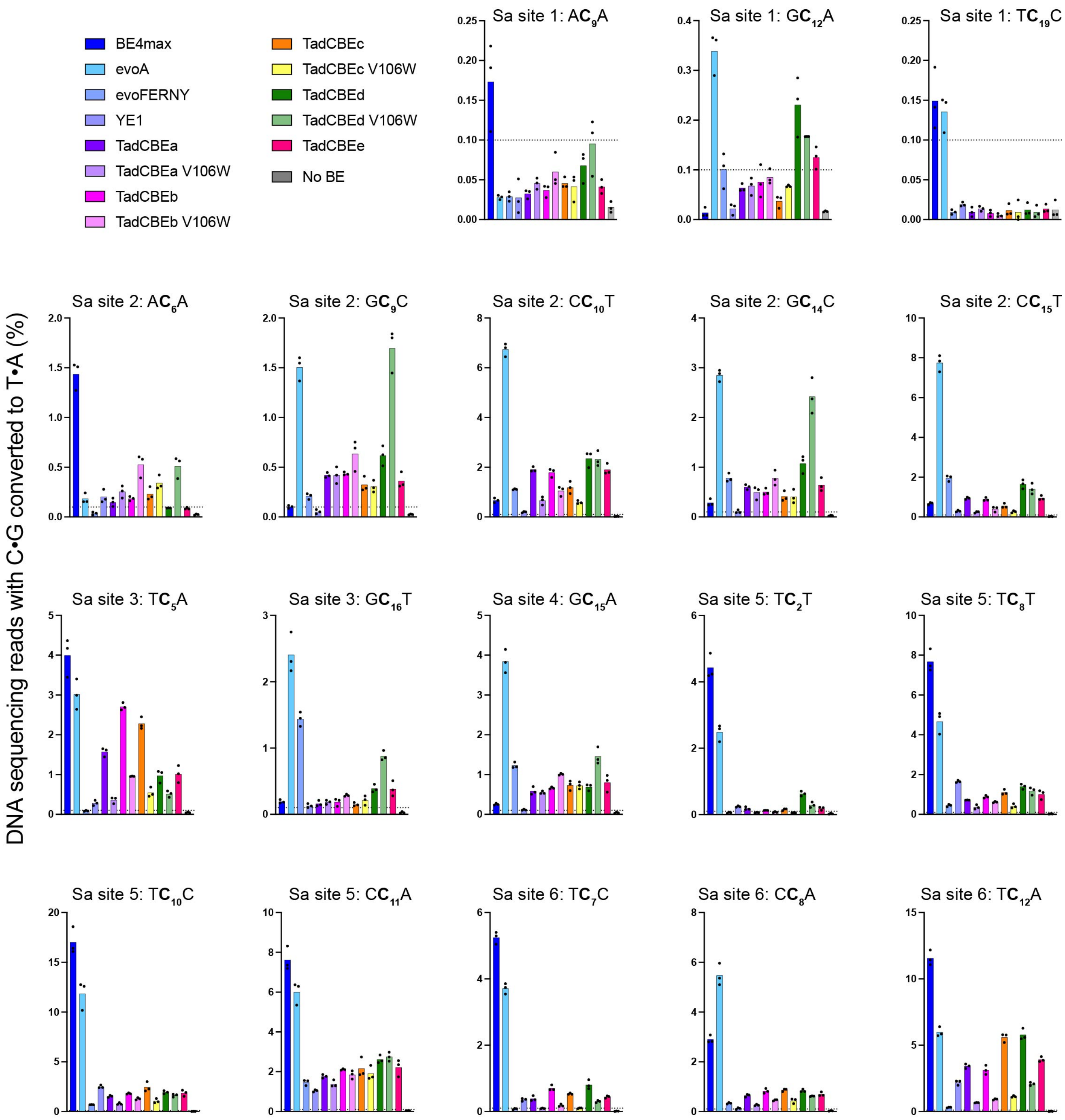

Supplementary Figure 14. Cas-independent off-target C•G-to-T•A editing at individual sites within six orthogonal R-loops generated by SaCas9. The previously published orthogonal R-loop assay was performed on CBE variants in the BE4max architecture[7]. Cells were transfected with the base editor and one SpCas9 sgRNA targeting the *EMX1* locus along with orthogonal dead SaCas9 and one SaCas9 sgRNA corresponding to Sa sites 1–6. Dots represent individual biological replicates and bars represent mean±s.d. from three independent biological replicates. Corresponding on-target data are in Supplementary Fig. 13.

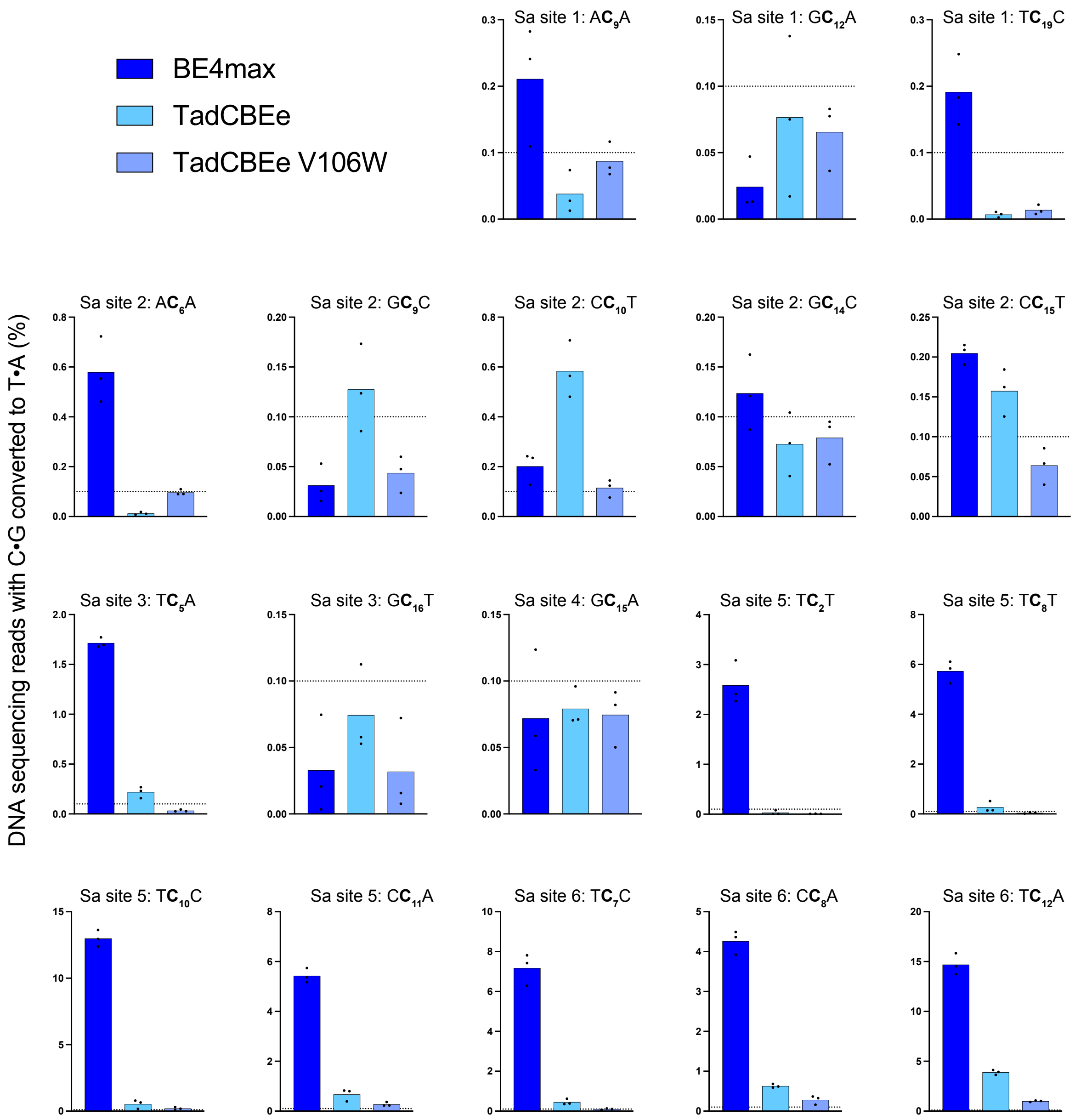

Supplementary Figure 15. Cas-independent off-target C•G-to-T•A editing by TadCBEe V106W at individual sites within six orthogonal R-loops generated by SaCas9. The previously published orthogonal R-loop assay was performed on CBE variants in the BE4max architecture[7]. Cells were transfected with the base editor and one SpCas9 sgRNA targeting the *EMX1* locus along with orthogonal dead SaCas9 and one SaCas9 sgRNA corresponding to Sa sites 1–6. Dots represent individual biological replicates and bars represent mean±s.d. from three independent biological replicates.

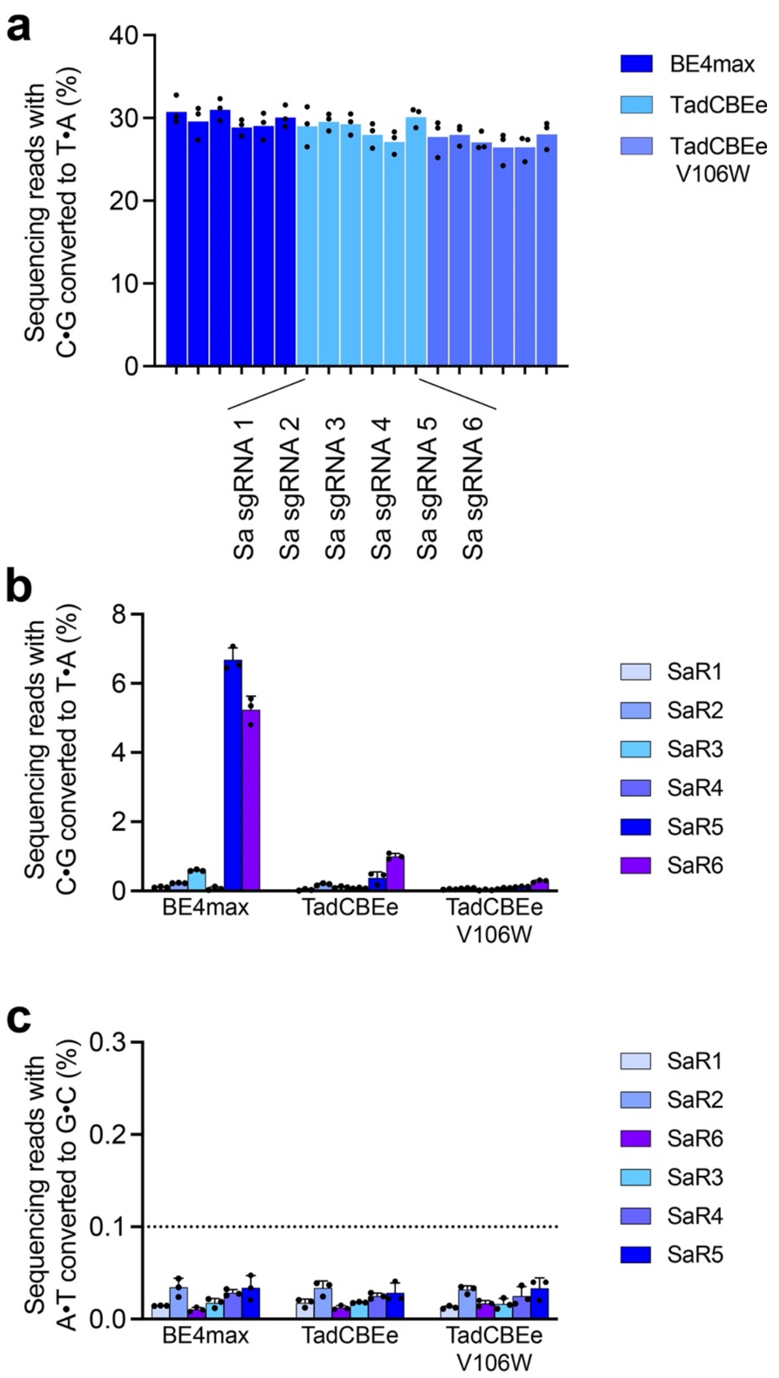

Supplementary Figure 16. Cas-independent off-target DNA editing by TadCBEe V106W at six genomic SaCas9 R-loops. The previously published orthogonal R-loop assay was performed on CBE variants in the BE4max architecture[7]. Cells were transfected with the base editor and one SpCas9 sgRNA targeting the *EMX1* locus (on-target) along with orthogonal dead SaCas9 and one SaCas9 sgRNA corresponding to Sa sites 1–6 (SaR1–SaR6). **(a)** On-target editing at the *EMX1* locus. **(b)** The average C•G-to-T•A base editing across all the adenines within the indicated protospacer is depicted on the graph. **(c)** The average A•T-to-G•C base editing across all the adenines within the indicated protospacer is depicted on the graph. Dots represent individual biological replicates and bars represent mean±s.d. from three independent biological replicates.

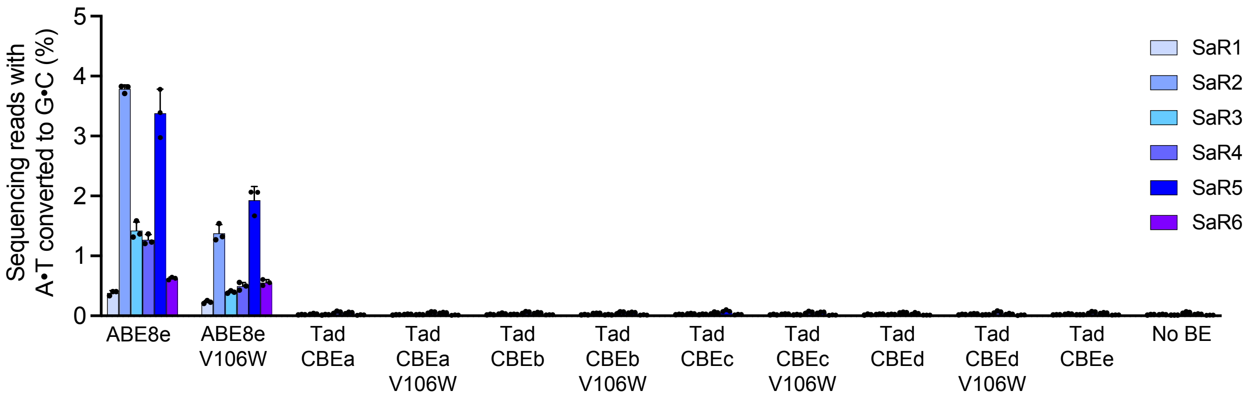

Supplementary Figure 17. Cas-independent off-target DNA editing at six genomic SaCas9 R-loops. The previously published orthogonal R-loop assay was performed on CBE variants in the BE4max architecture[7]. Cells were transfected with the base editor and one SpCas9 sgRNA targeting the *EMX1* locus (on-target) along with orthogonal dead SaCas9 and one SaCas9 sgRNA corresponding to Sa sites 1–6 (SaR1–SaR6). The average A•T-to-G•C base editing across all the adenines within the indicated protospacer is depicted on the graph. Dots represent individual biological replicates and bars represent mean±s.d. from three independent biological replicates.

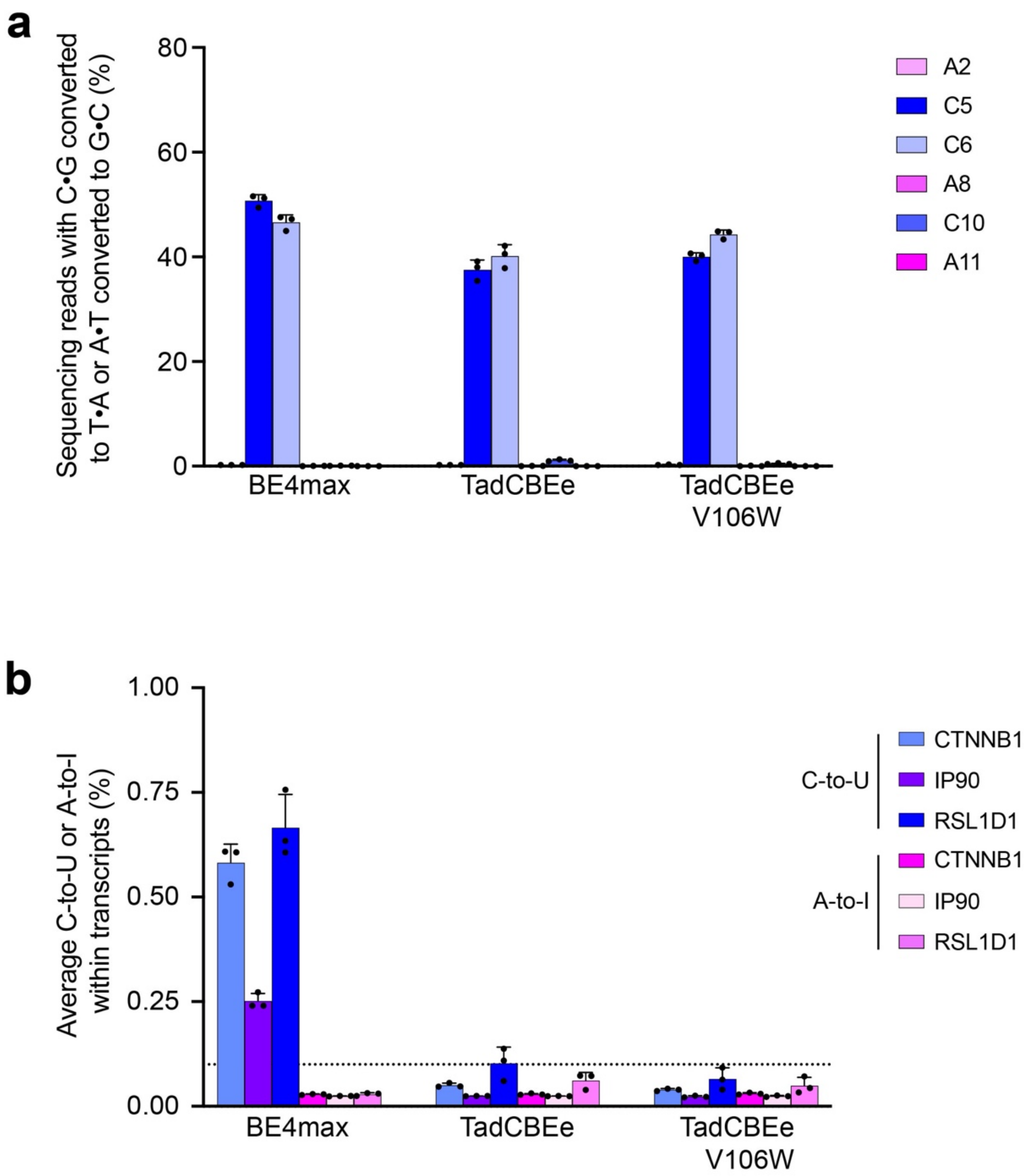

Supplementary Figure 18. Cas-independent off-target RNA editing of all cytosines and adenines examined across three transcripts for TadCBEe V106W. Total RNA was harvested from HEK293T cells 48 hours after transfection with the indicated base editor. Following cDNA synthesis, *CTNNB1*, *IP90*, and *RSL1D1* were amplified and analyzed by high-throughput sequencing. At the same time, genomic DNA was harvested from the other plate that was transfected in parallel. The genomic DNA was analyzed for on-target editing of *EMX1* as a control for base editor activity. **(a)** On-target editing of *EMX1* in samples corresponding to the RNA editing analysis. **(b)** Average C-to-U (shades of blue) or A-to-I (shades of magenta) editing across transcripts. Dots represent individual biological replicates and bars represent mean±s.d. of three independent biological replicates.

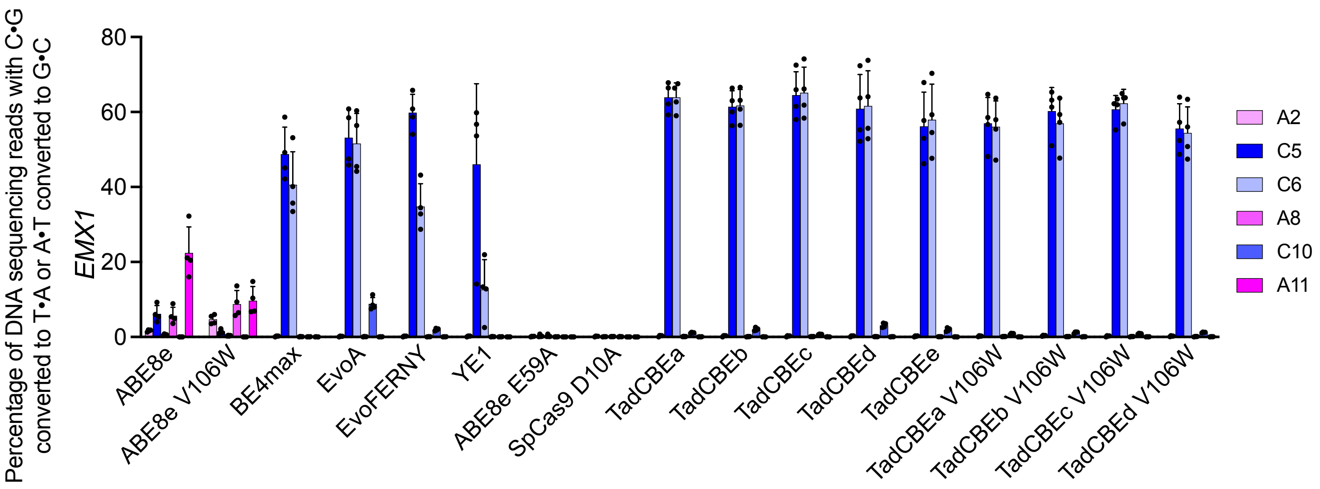

Supplementary Figure 19. On-target editing of *EMX1* in the RNA off-target editing experiment. The indicated base editor was transfected into HEK293T cells in two parallel plates. In one plate, RNA was harvested from HEK293T cells 48 hours after transfection with the indicated base editor and analyzed as described in Supplementary Fig. 18. At the same time, genomic DNA was harvested from the other plate that was transfected in parallel. The genomic DNA was analyzed for on-target editing of *EMX1* as a control for base editor activity. Dots represent individual biological replicates and bars represent mean±s.d. of three independent biological replicates.

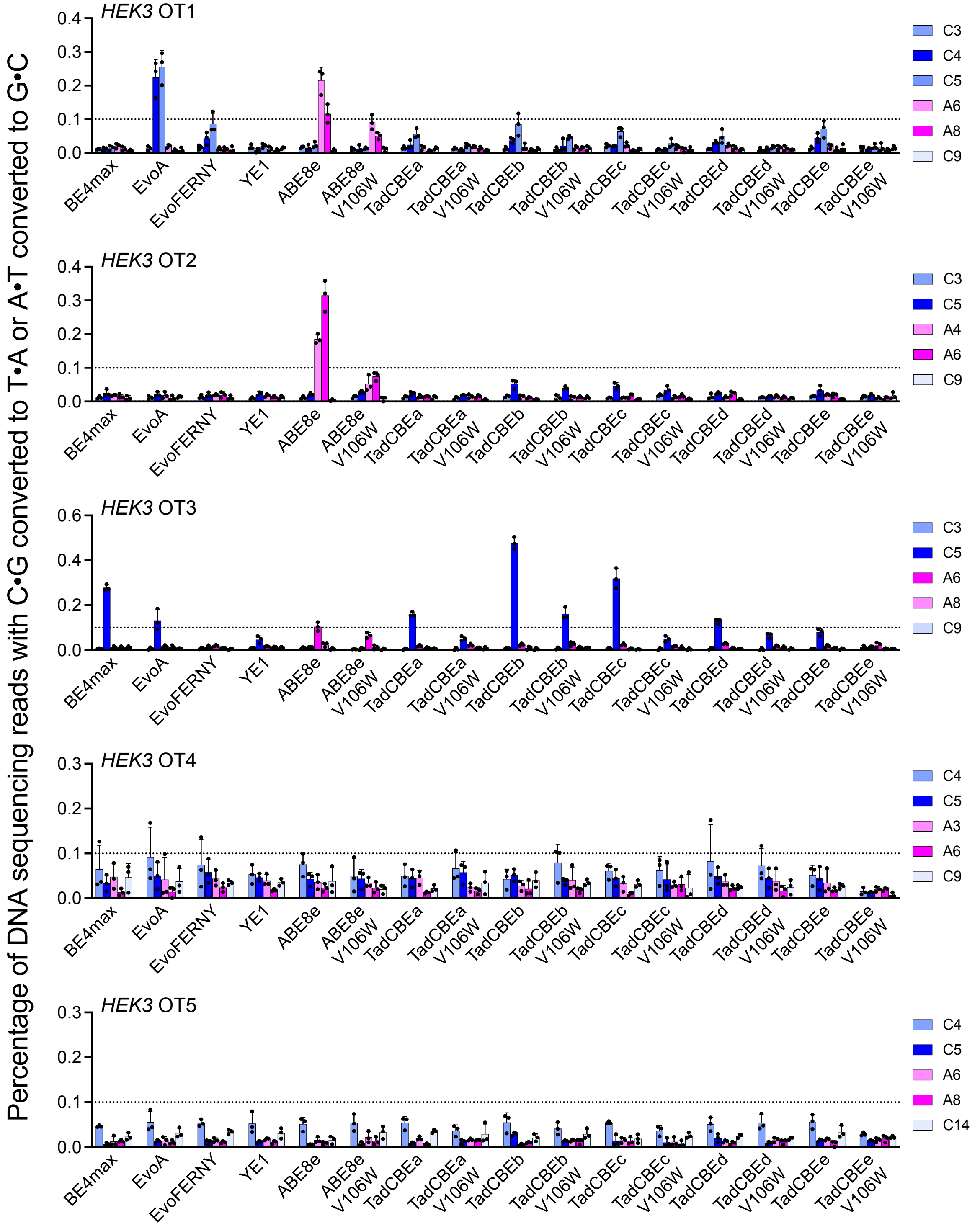

Supplementary Figure 20. Cas-dependent editing of known off-target sites for *HEK3*. The specified base editors using SpCas9 nickase domains in the BE4max architecture or ABE8e with 2xUGI were transfected into HEK293T cells along with a guide RNA targeting HEK293T site 3 (*HEK3*). 72 h after transfection, genomic DNA was harvested and known off-target sites were amplified using the primers in Supplementary Table 4. C•G-to-T•A base editing is shown in shades of blue. A•T-to-G•C base editing is shown in shades of magenta. Dots represent individual values and bars represent mean±s.d. of three independent biological replicates.

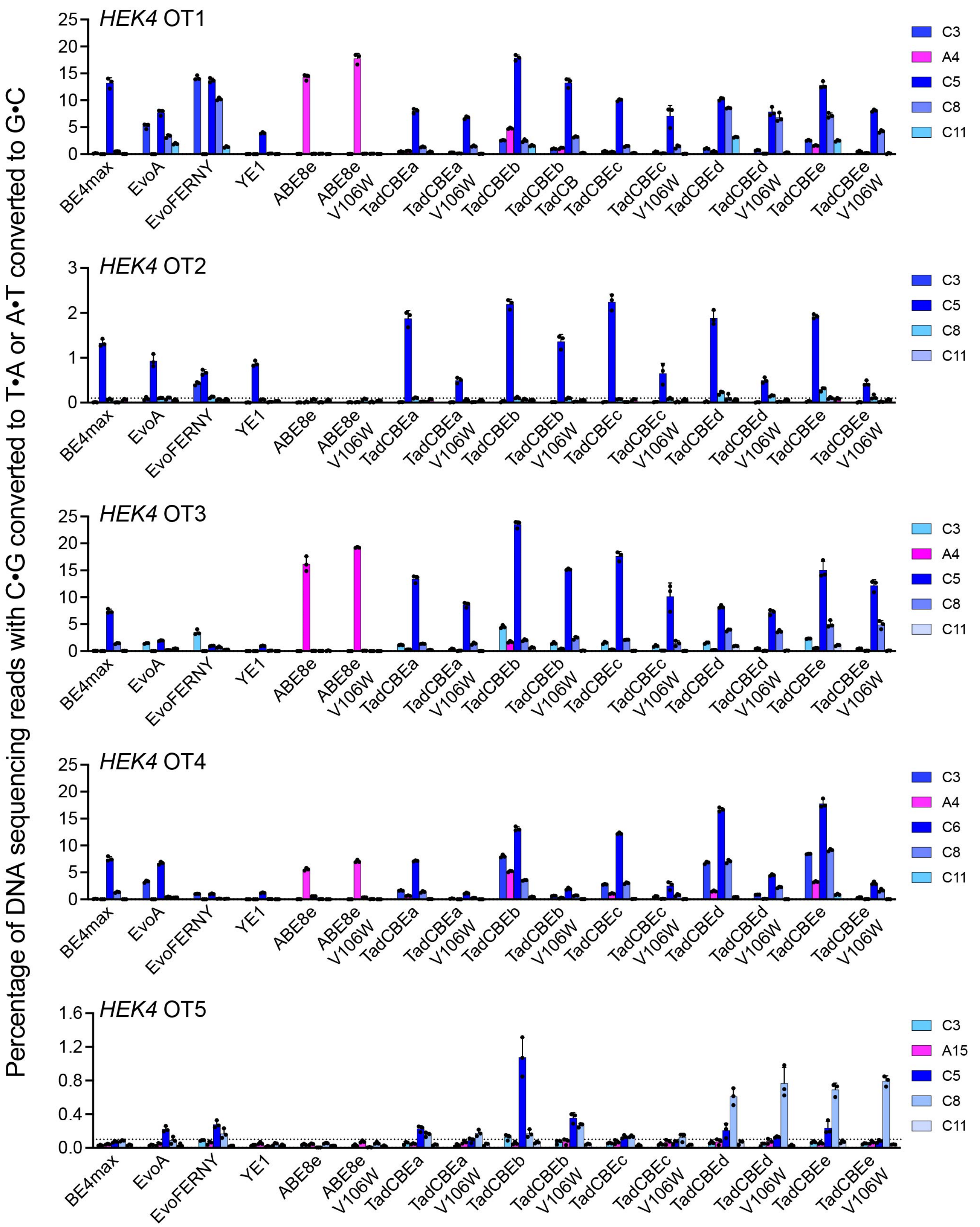

Supplementary Figure 21. Cas-dependent editing of known off-target sites for *HEK4*. The specified base editors using SpCas9 nickase domains in the BE4max architecture or ABE8e with 2xUGI were transfected into HEK293T cells along with a guide RNA targeting HEK293T site 4 (*HEK4*). 72 h after transfection, genomic DNA was harvested and known off-target sites were amplified using the primers in Supplementary Table 4. C•G-to-T•A base editing is shown in shades of blue. A•T-to-G•C base editing is shown in shades of magenta. Dots represent individual values and bars represent mean±s.d. of three independent biological replicates.

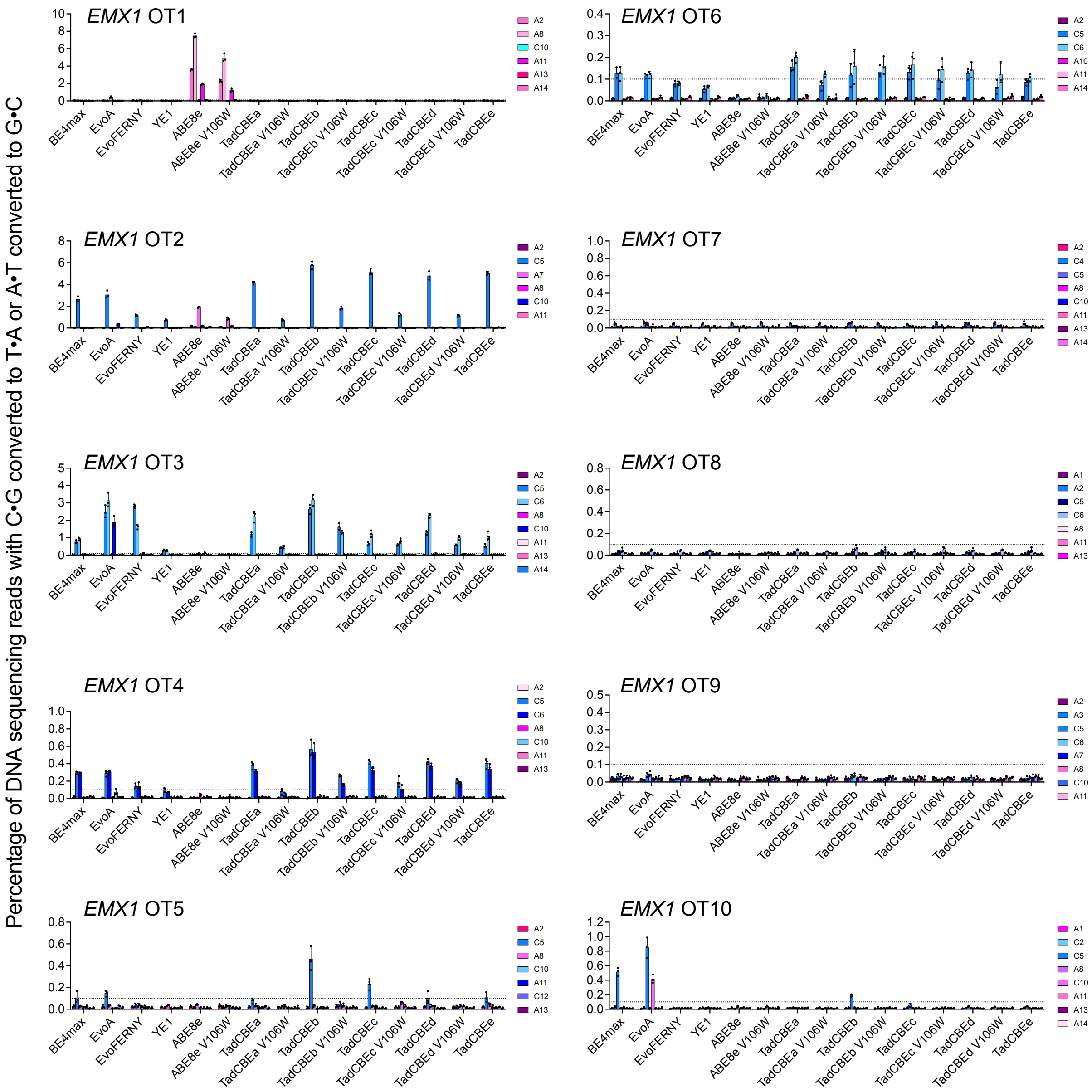

Supplementary Figure 22. Cas-dependent editing of known off-target sites for *EMX1*. The specified base editors using SpCas9 nickase domains in the BE4max architecture or ABE8e with 2xUGI were transfected into HEK293T cells along with a guide RNA targeting *EMX1*. 72 h after transfection, genomic DNA was harvested and known off-target sites were amplified using the primers in Supplementary Table 4. C•G-to-T•A base editing is shown in shades of blue. A•T-to-G•C base editing is shown in shades of magenta. Dots represent individual values and bars represent mean±s.d. of three independent biological replicates. The corresponding on-target data are in Supplementary Figure 23.

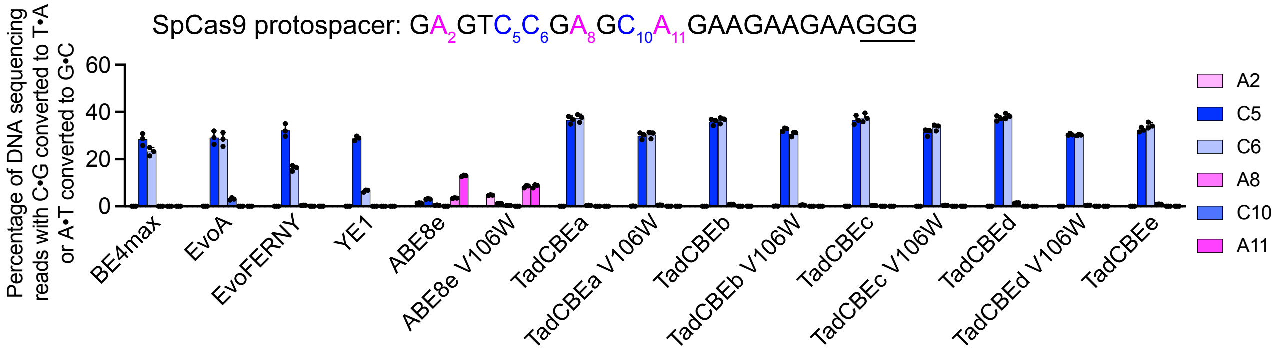

Supplementary Figure 23. On-target editing of EMX1. The specified base editors using SpCas9 nickase domains in the BE4max architecture or ABE8e with 2xUGI were transfected into HEK293T cells along with a guide RNA targeting *EMX1*. 72 h after transfection, genomic DNA was harvested and known off-target sites were amplified using the primers in Supplementary Table 4. C•G-to-T•A base editing is shown in shades of blue. A•T-to-G•C base editing is shown in shades of magenta. Dots represent individual values and bars represent mean±s.d. of three independent biological replicates. The corresponding off-target data are in Supplementary Figure 22.

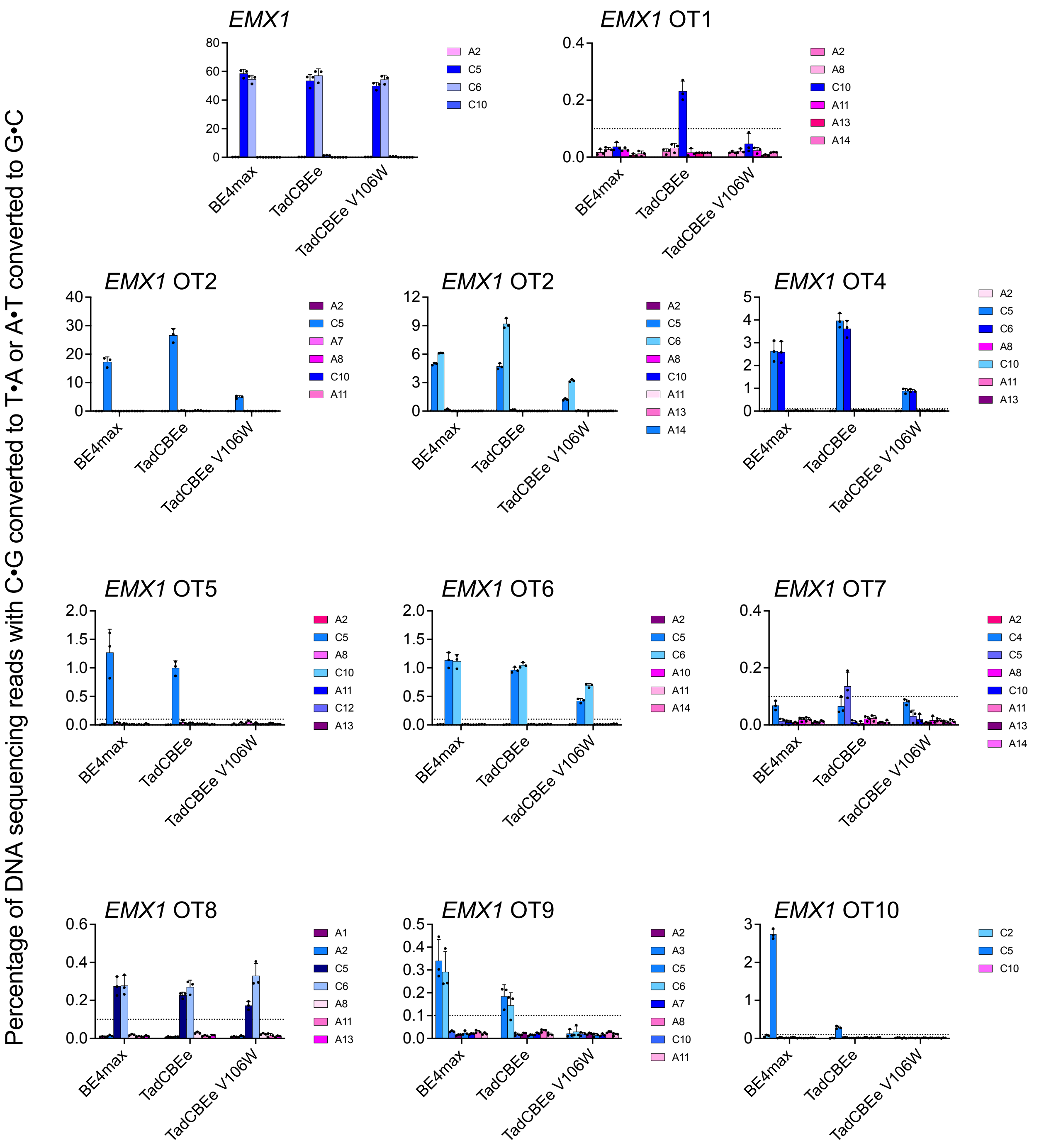

Supplementary Figure 24. On-target and off-target editing of *EMX1* by TadCBE V106W. The specified base editors using SpCas9 nickase domains in the BE4max architecture or ABE8e with 2xUGI were transfected into HEK293T cells along with a guide RNA targeting *EMX1*. 72 h after transfection, genomic DNA was harvested and known off-target sites were amplified using the primers in Supplementary Table 4. C•G-to-T•A base editing is shown in shades of blue. A•T-to-G•C base editing is shown in shades of magenta. Dots represent individual values and bars represent mean±s.d. of three independent biological replicates.

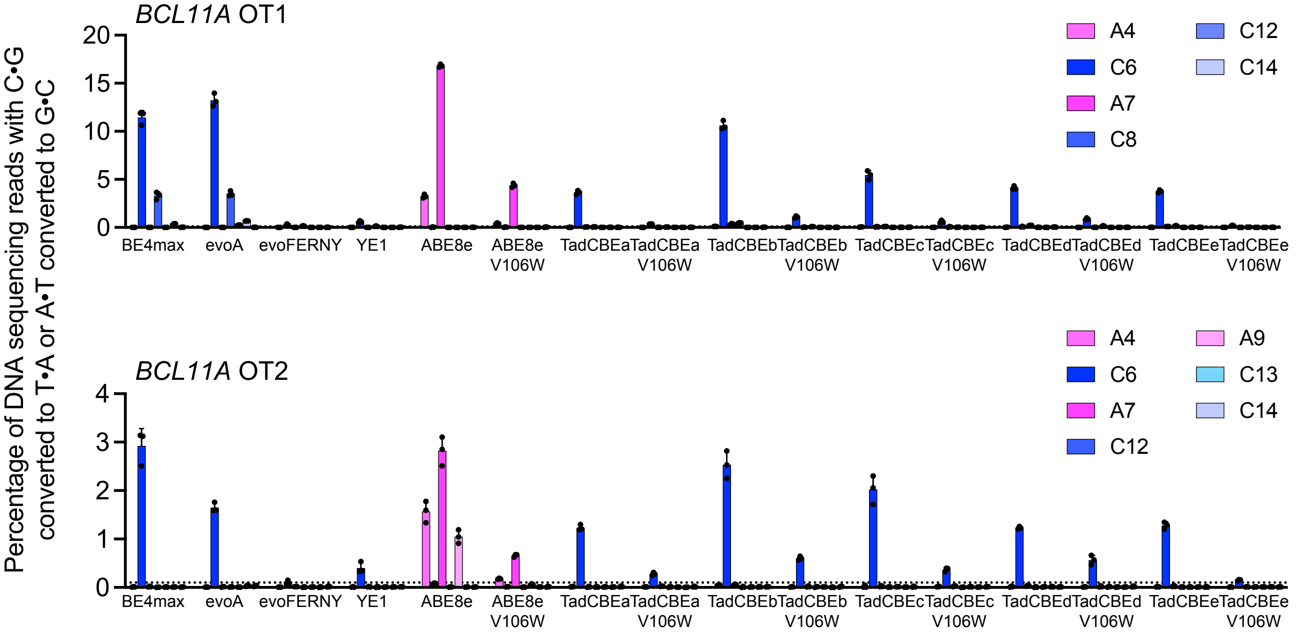

Supplementary Figure 25. **Cas-dependent editing of known off-target sites for *BCL11A.*** The specified base editors using SpCas9 nickase domains in the BE4max architecture or ABE8e with 2xUGI were transfected into primary human CD34-positive hematopoietic stem and progenitor cells (n=3 donors) along with a guide RNA targeting *BCL11A*. 72 h after transfection, genomic DNA was harvested and known off-target sites were amplified using the primers in Supplementary Table 4. C•G-to-T•A base editing is shown in shades of blue. A•T-to-G•C base editing is shown in shades of magenta. Dots represent individual values and bars represent mean±s.d. of three independent biological replicates.

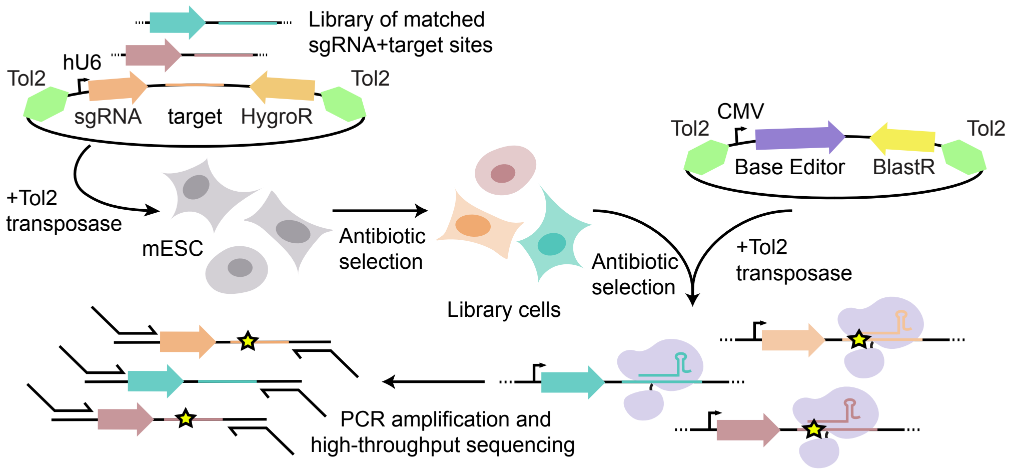

Supplementary Figure 26. **Schematic of mESC library experiment*.*** Thousands of pairs of sgRNAs and corresponding target sites are integrated into mESCs and treated with base editors. Base editor-containing cells are enriched by antibiotic selection, and library cassettes are amplified for high-throughput sequencing. Adapted from Arbab, Shen, *et al. Cell* 2020.

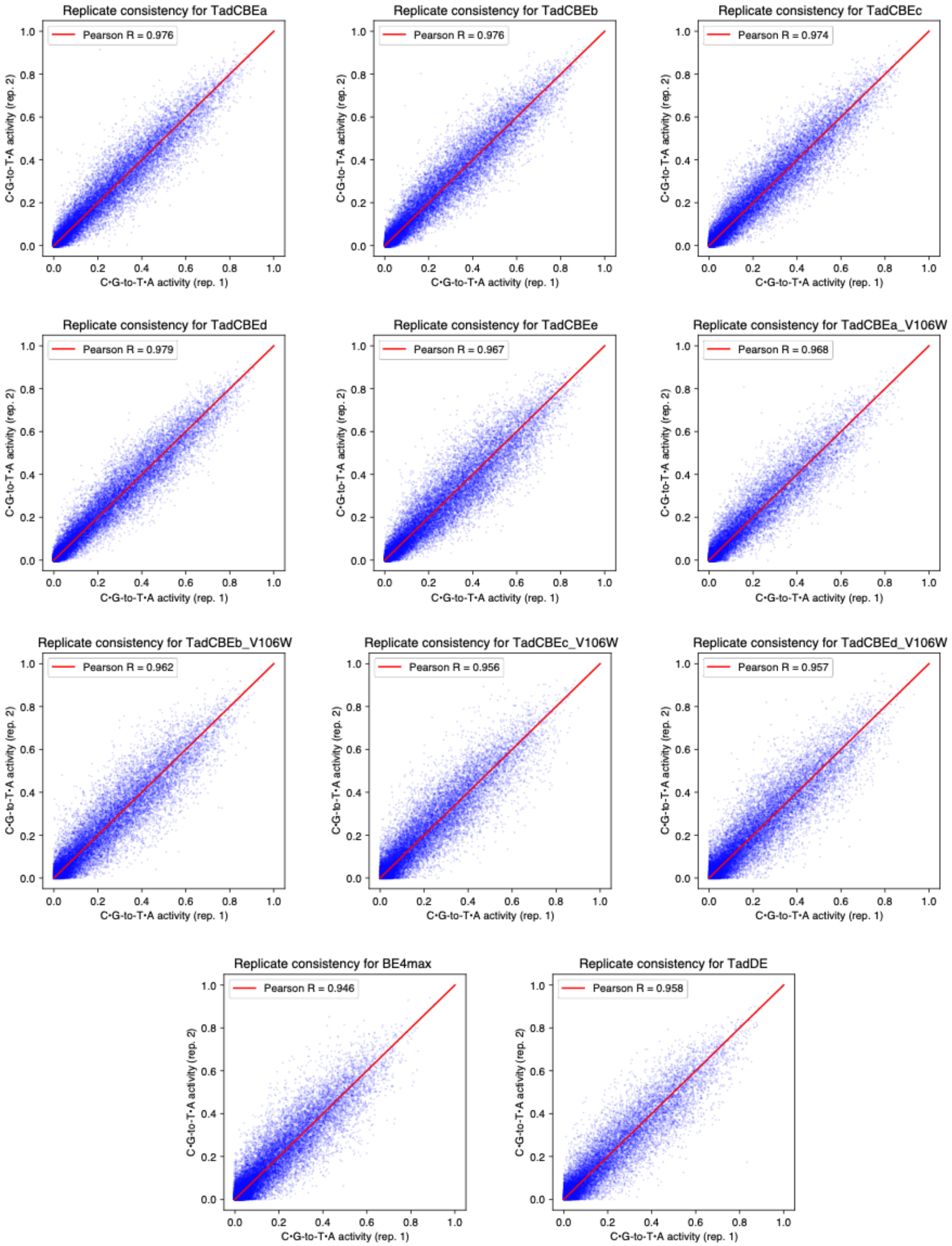

Supplementary Figure 27. **Correlation between replicates in the mESC library experiment.** Uncorrected C•G-to-T•A editing efficiency at each target site for each replicate. The red dashed line is a total least-squares regression line.

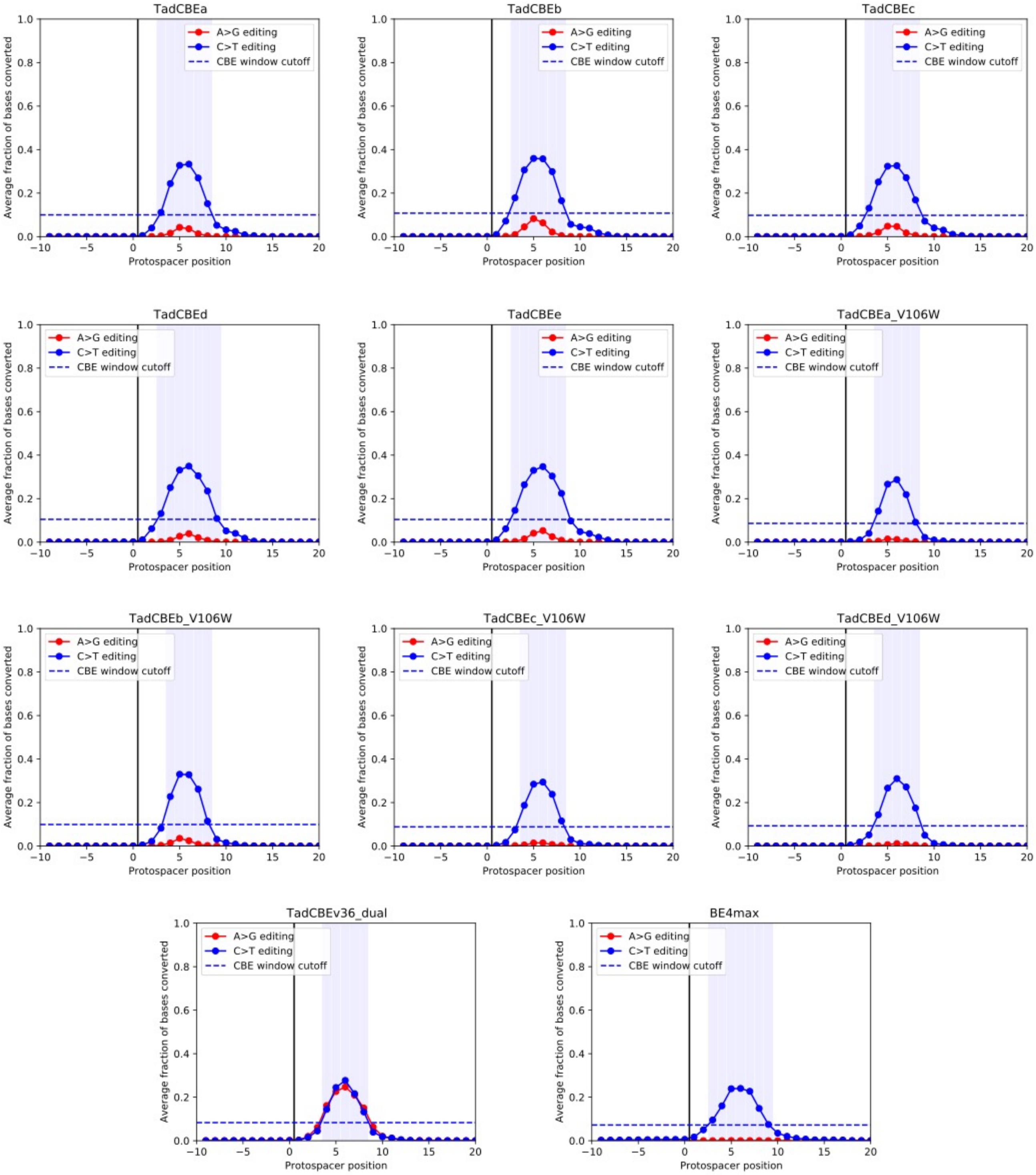

Supplementary Figure 28. **Editing windows of TadCBE V106W variants in the mESC library editing experiment.** The editing window is defined as positions within the protospacer where the average fraction of converted bases at that position is at least 30% of the average editing at the maximally edited position. C•G-to-T•A base editing is shown in blue. A•T-to-G•C base editing is shown in red.

a

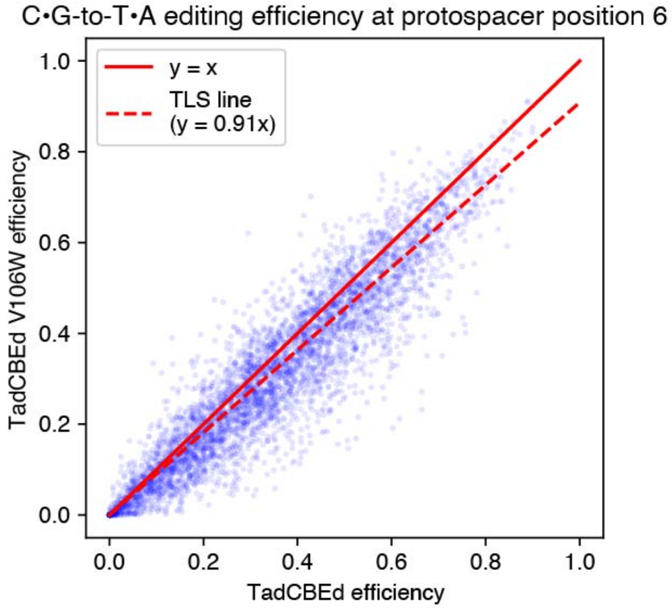

b

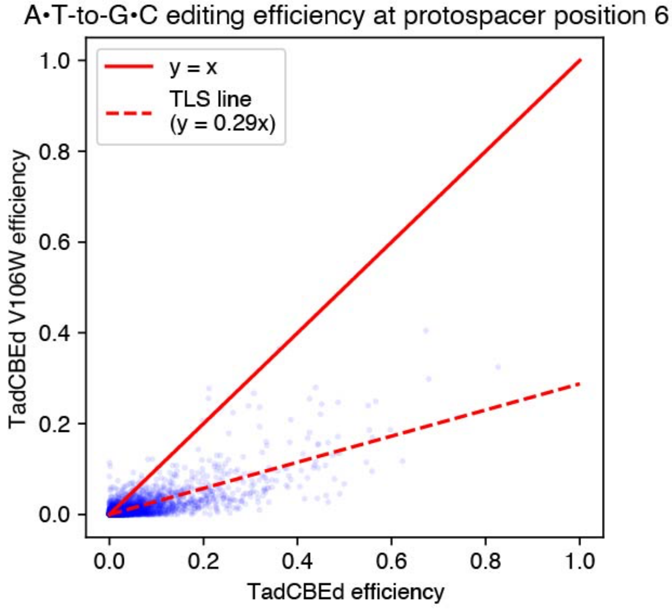

Supplementary Figure 29. Effect of V106W on peak editing in the mESC library experiment. (a) C•G-to-T•A editing efficiency with TadCBEd (with and without V106W) for each library member containing a cytosine at protospacer position 6. The red dashed line is a total least-squares regression line. **(b)** A•T-to-G•C editing efficiency with TadCBEd (with and without V106W) for each library member containing an adenine at protospacer position 6. The red dashed line is a total least-squares regression line.

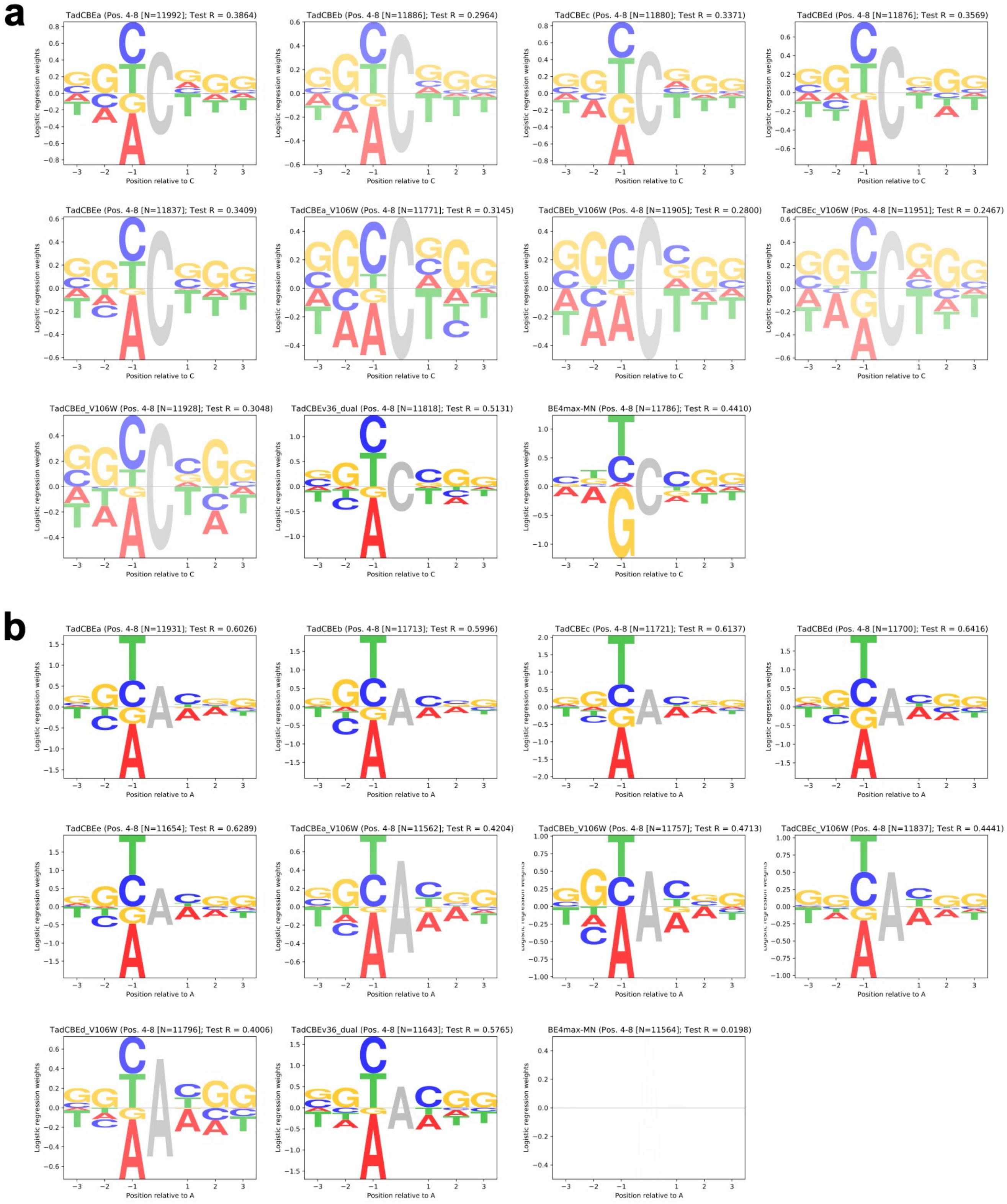

Supplementary Figure 30. **Sequence motifs for context preferences of TadCBEs.** Sequence motifs for base editing activities from performing regression on the editing efficiencies. Logo opacity is proportional to the *R* on a held-out test set. See Methods. Plots are provided for **(a)** C•G-to-T•A base editing and for **(b)** A•T-to-G•C base editing.

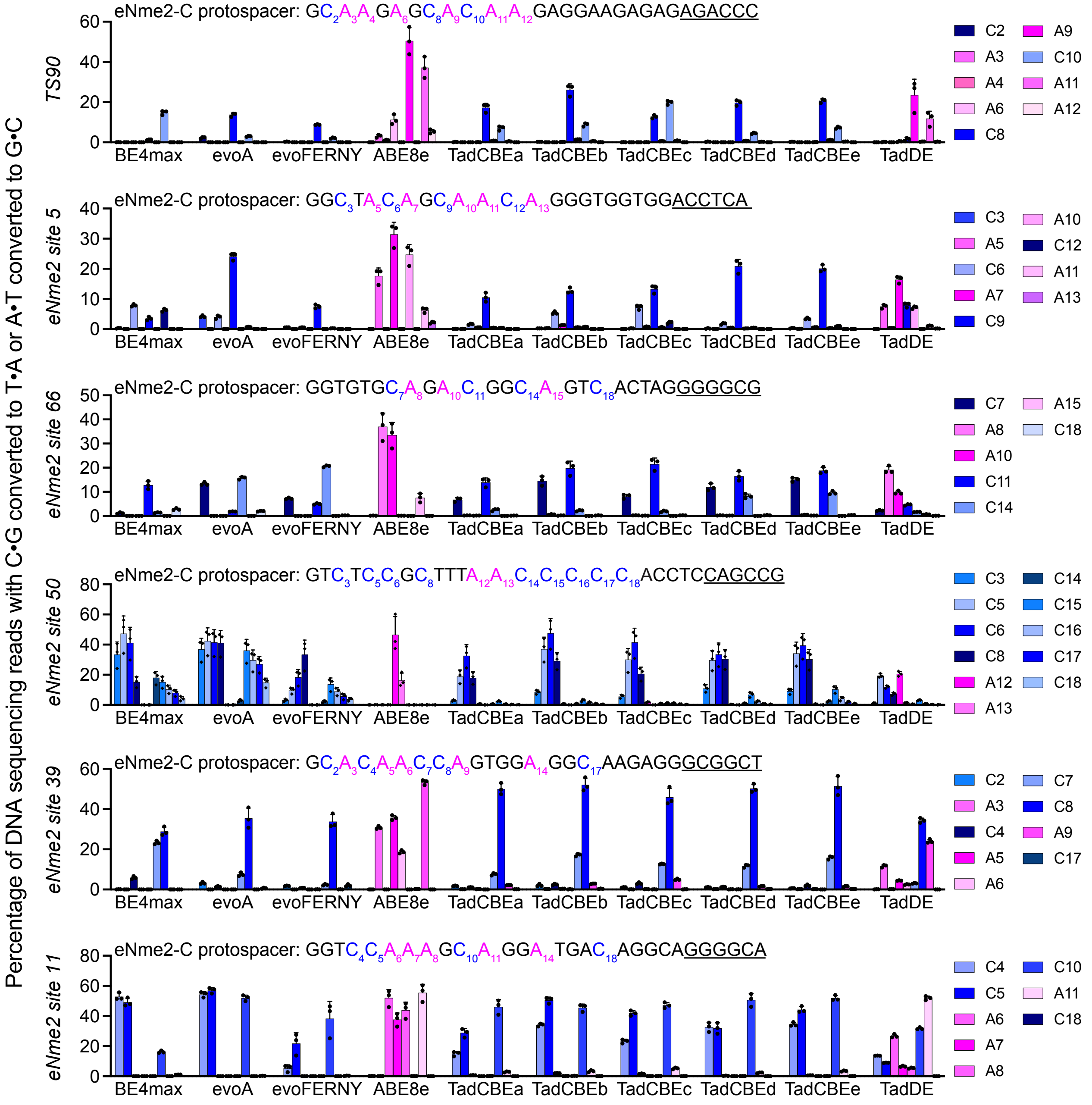

Supplementary Figure 31. Characterization of evolved deaminases with evolved eNme2-C Cas9 domains. The specified base editors using eNme2-C Cas9 nickase domains (PAM=N_4CN) in the BE4max architecture or ABE8e with 2xUGI were transfected into HEK293T cells along with each of six guide RNAs targeting the protospacers shown in each graph. Target cytosines are blue, target adenines are magenta, and PAM sequences are underlined. C•G-to-T•A base editing is shown in shades of blue. A•T-to-G•C base editing is shown in shades of magenta. Dots represent individual values and bars represent mean±s.d. of three independent biological replicates.

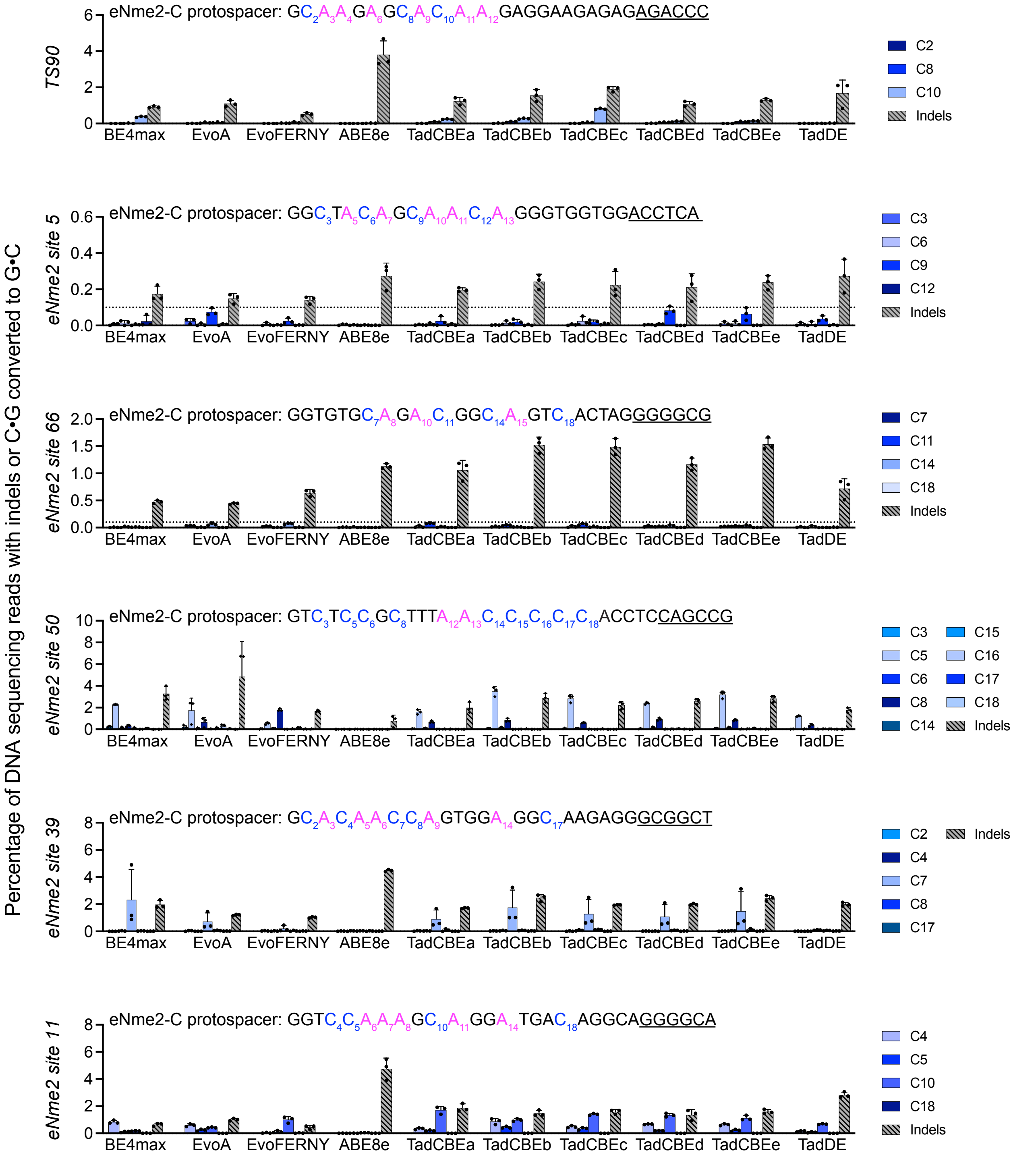

Supplementary Figure 32. Indels and C•G-to-G•C editing by eNme2-C Cas9 variants at six genomic target sites. The specified base editors using eNme2-C Cas9 nickase domains in the BE4max architecture or ABE8e with 2xUGI were transfected into HEK293T cells along with each of six guide RNAs targeting the protospacers shown in each graph. C•G-to-G•C base editing is shown in shades of blue. Indels are shown in grey. Dots represent individual values and bars represent mean±s.d. of three independent biological replicates. The corresponding on-target data are in Supplementary Figure 31.

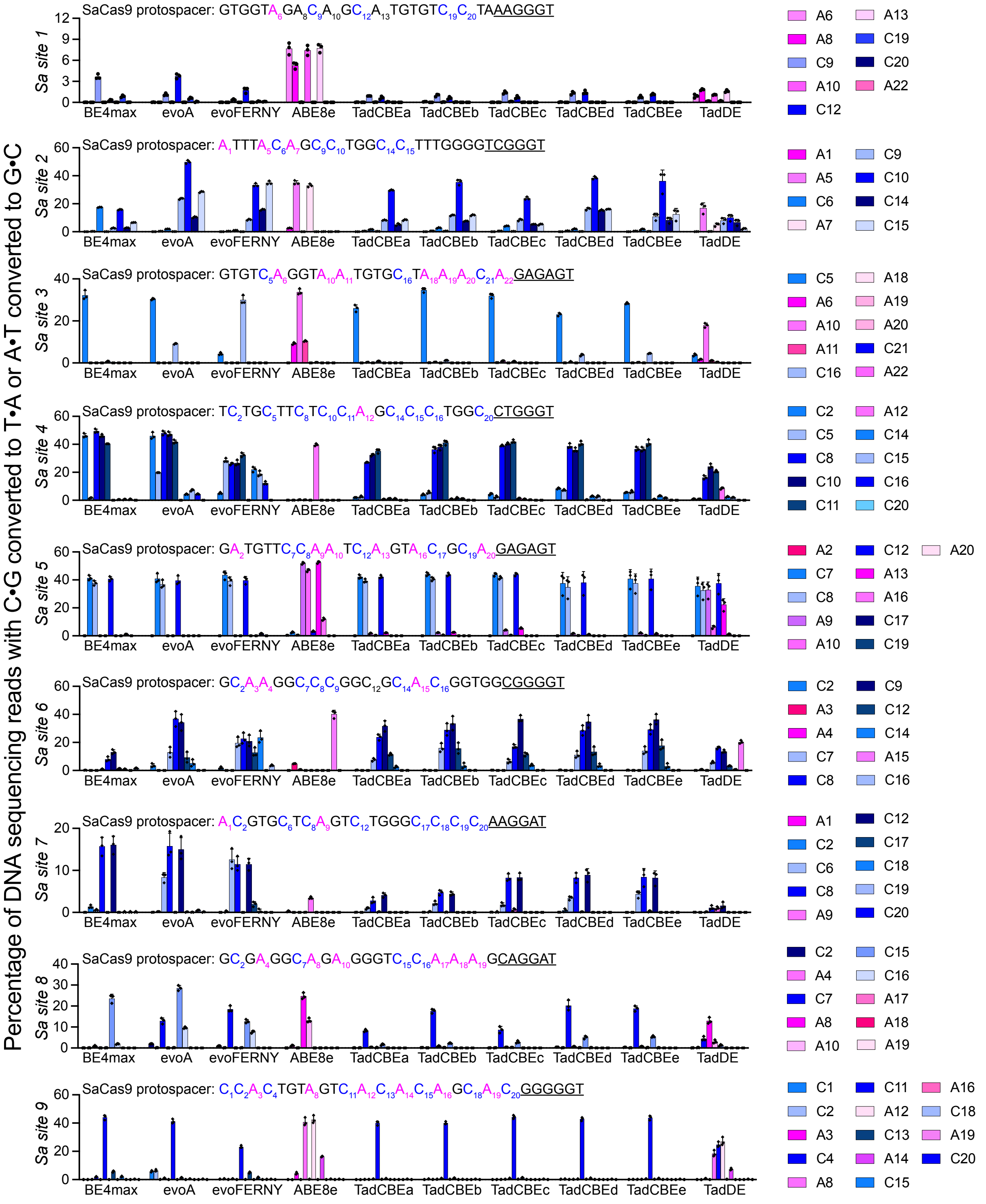

Supplementary Figure 33. Characterization of evolved deaminases with SaCas9 domains. The specified base editors using SaCas9 nickase domains (PAM=NNGRRT) in the BE4max architecture or ABE8e with 2xUGI were transfected into HEK293-T cells along with each of nine guide RNAs targeting the protospacers shown in each graph. Target cytosines are blue, target adenines are magenta, and PAM sequences are underlined. C•G-to-T•A base editing is shown in shades of blue. A•T-to-G•C base editing is shown in shades of magenta. Dots represent individual values and bars represent mean±s.d. of three independent biological replicates.

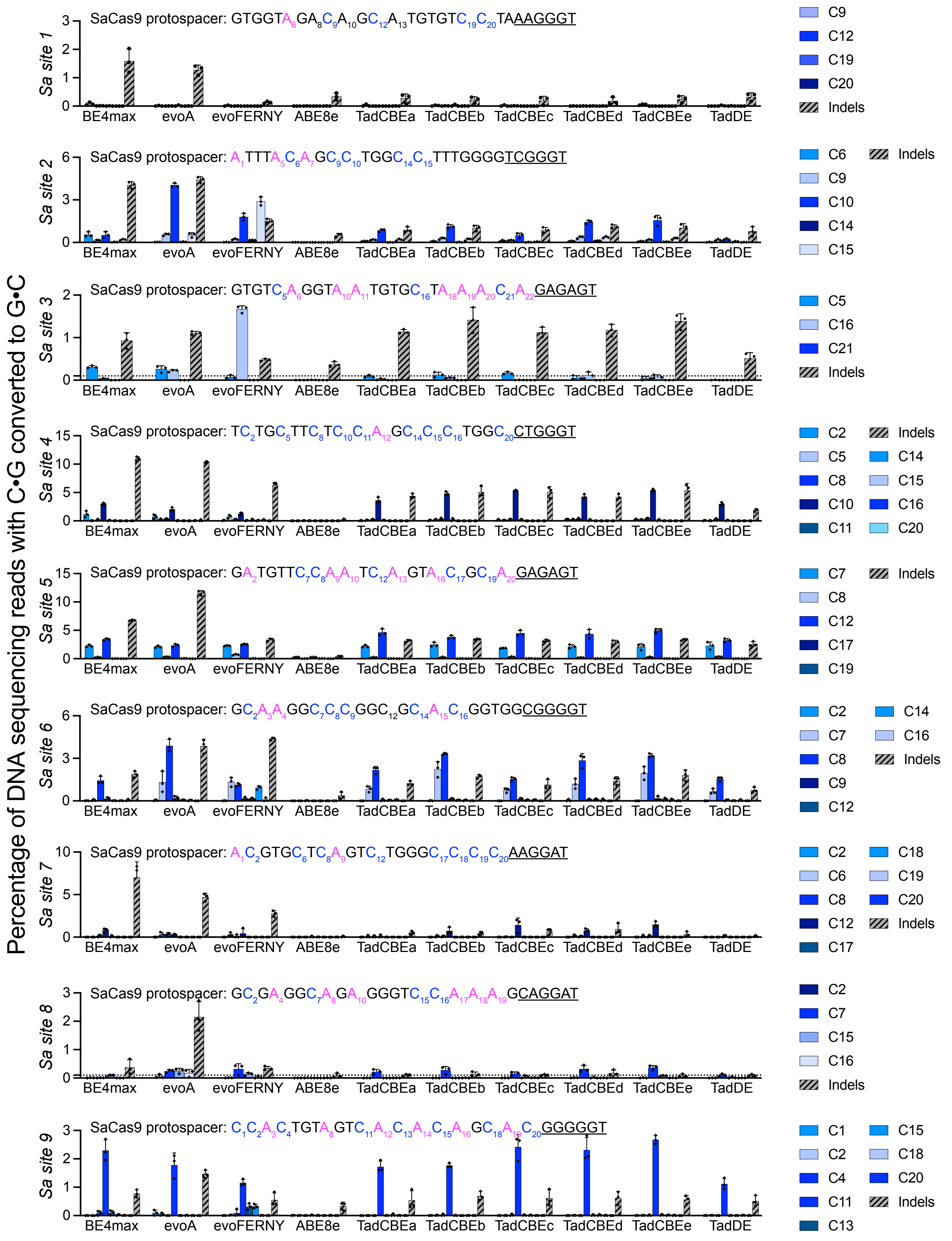

Supplementary Figure 34. Indels and C•G-to-G•C editing by SaCas9 variants at nine genomic target sites. The specified base editors using SaCas9 nickase domains in the

BE4max architecture or ABE8e with 2xUGI were transfected into HEK293T cells along with each of six guide RNAs targeting the protospacers shown in each graph. C•G-to-G•C base editing is shown in shades of blue. Indels are shown in grey. Dots represent individual values and bars represent mean±s.d. of three independent biological replicates. The corresponding on-target data are in Supplementary Figure 33.

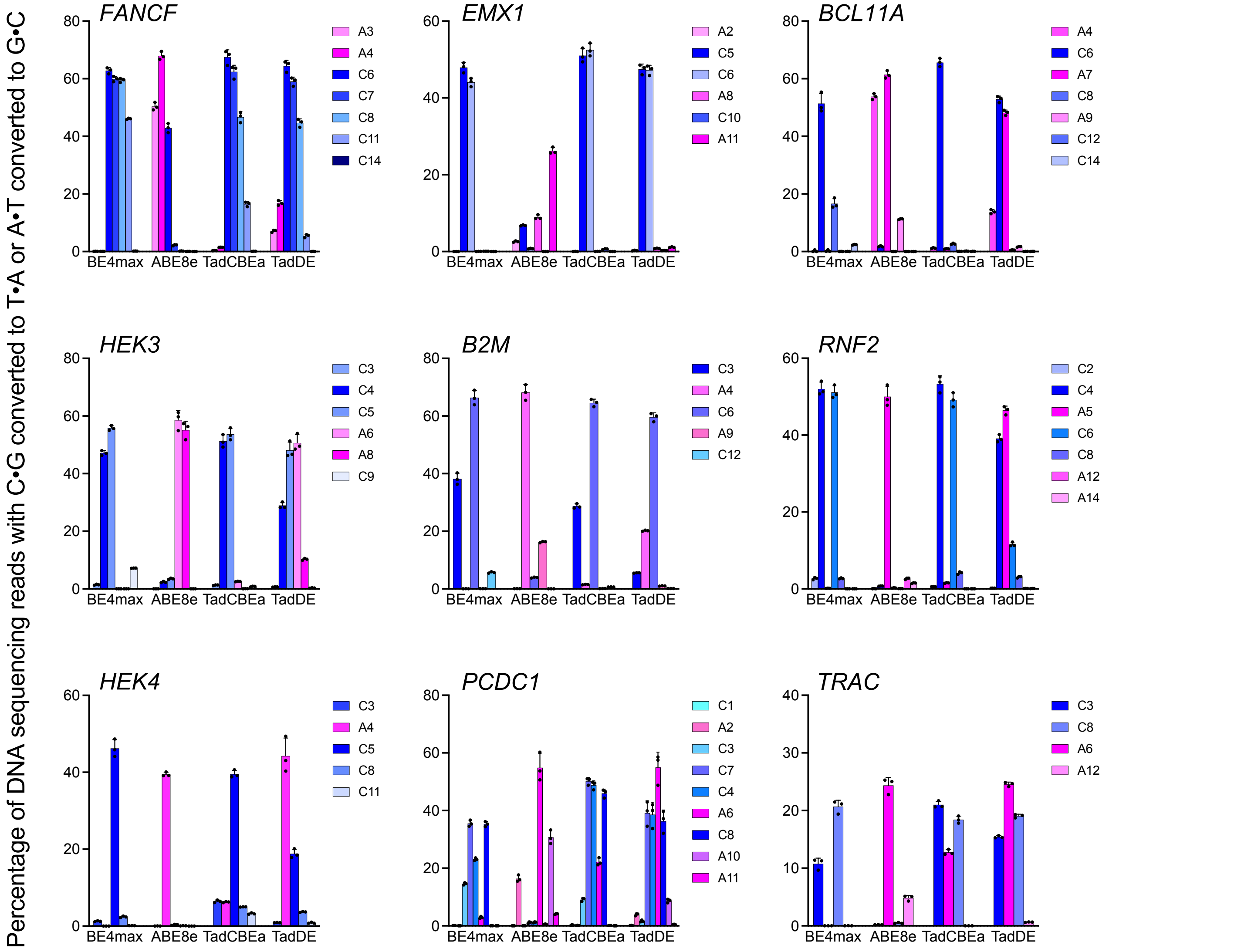

Supplementary Figure 35. Characterization of TadDE with SpCas9 in mammalian cells. The specified base editors using SpCas9 nickase domains (PAM=NGG) in the BE4max architecture or ABE8e with 2xUGI were transfected along with each of nine guide RNAs targeting the protospacers shown in each graph. Target cytosines are blue, target adenines are magenta, and PAM sequences are underlined. C•G-to-T•A base editing is shown in shades of blue. A•T-to-G•C base editing is shown in shades of magenta. Dots represent individual values and bars represent mean±s.d. of three independent biological replicates.

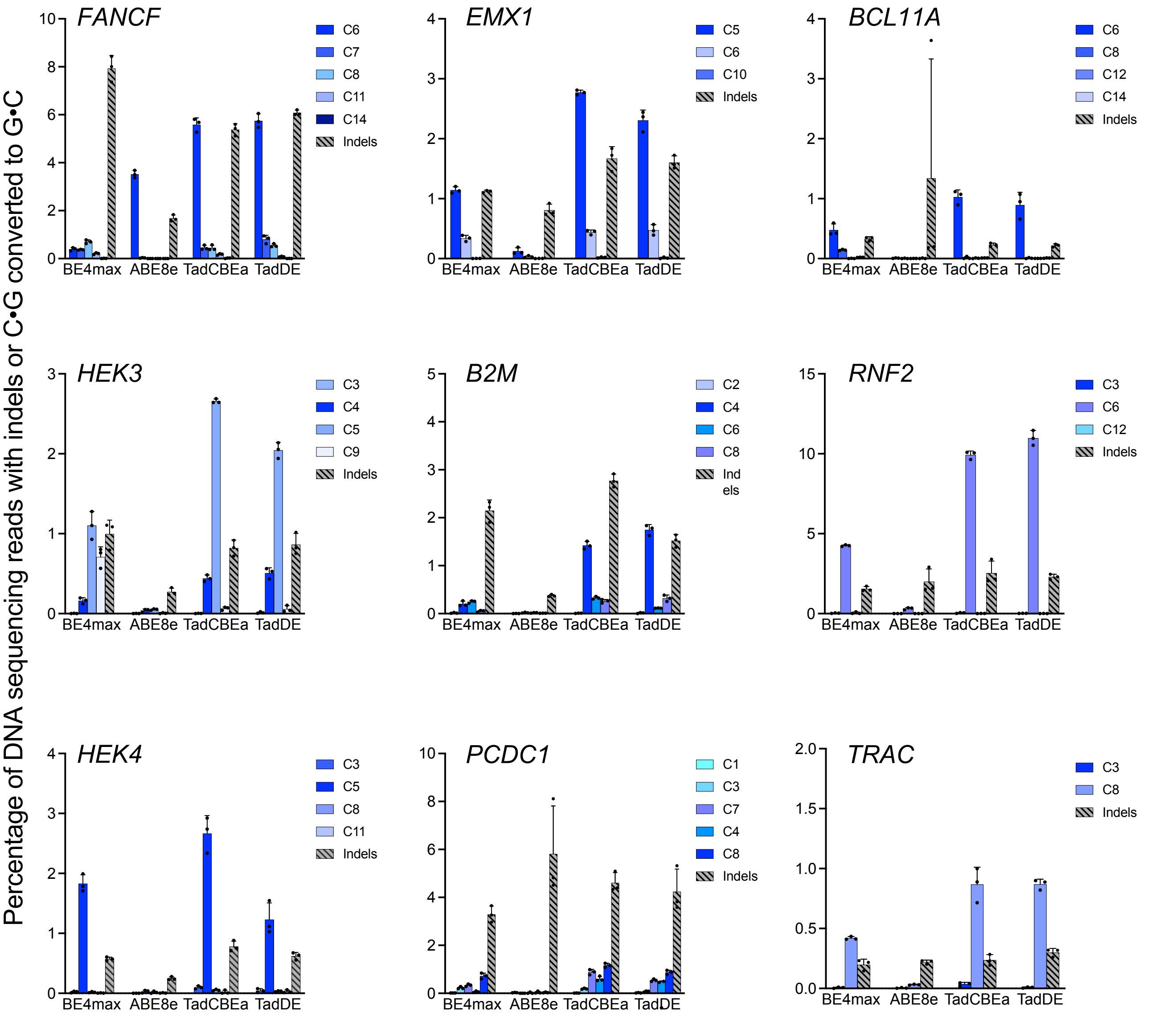

Supplementary Figure 36. Indels and C•G-to-G•C editing by SpCas9 variants at nine genomic target sites. The specified base editors using SpCas9 nickase domains in the BE4max architecture or ABE8e with 2xUGI were transfected into HEK293T cells along with each of six guide RNAs targeting the protospacers shown in each graph. C•G-to-G•C base editing is shown in shades of blue. Indels are shown in grey. Dots represent individual values and bars represent mean±s.d. of three independent biological replicates. The corresponding on-target data are in Supplementary Figure 35.

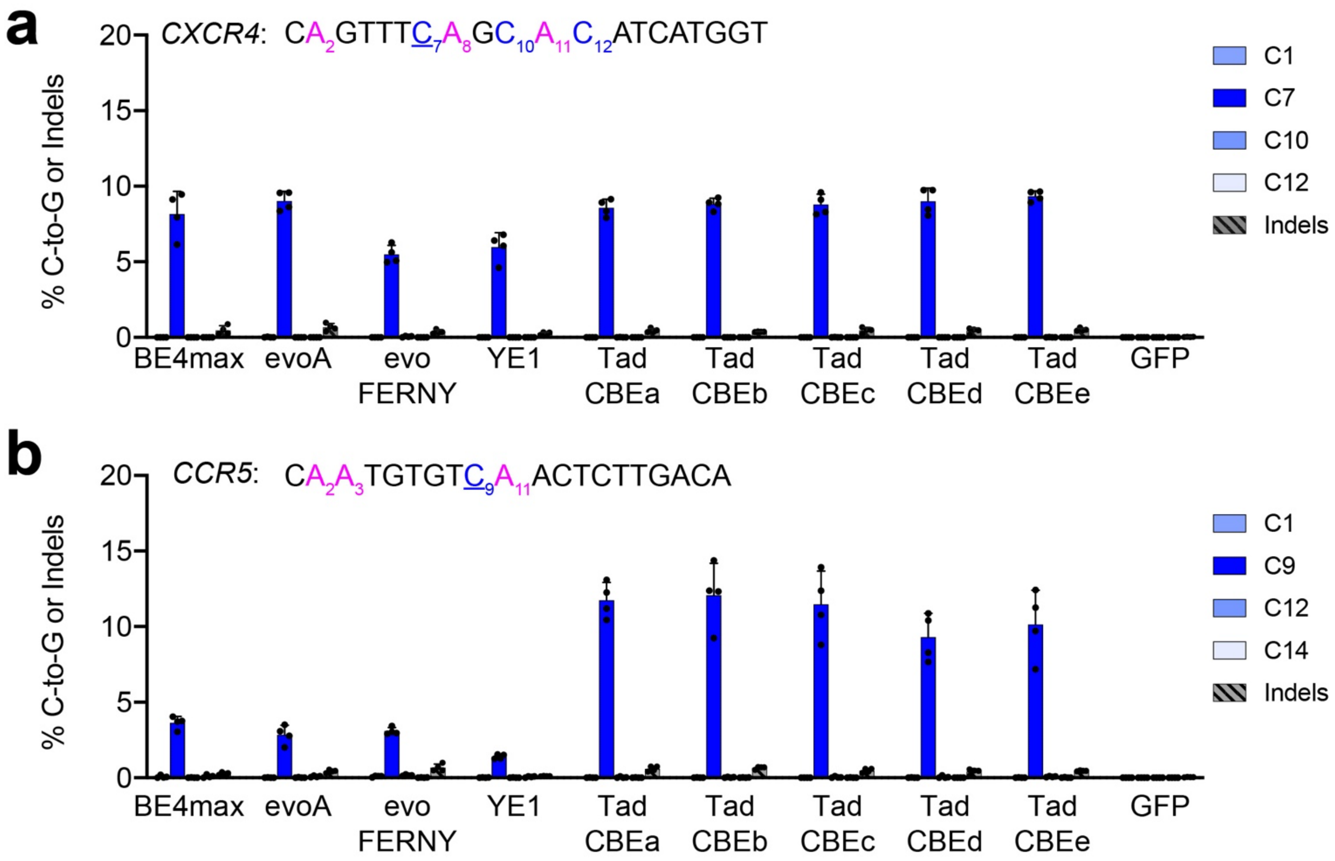

Supplementary Figure 37. C•G-to-G•C editing and indels for T-cell experiments targeting *CXCR4* and *CCR5*. mRNA encoding the indicated base editor or GFP as a negative control was electroporated into primary human T cells (n=4 donors) along with two synthetic guide RNAs targeting **(a**) *CXCR4* or **(b)** *CCR5* at the specified protospacers. After 3 days, genomic DNA was harvested from T-cell lysates and analyzed by high-throughput sequencing. C•G-to-G•C base editing is shown in shades of blue. Indels are shown in grey. Dots represent individual values and bars represent mean±s.d. of three independent biological replicates.

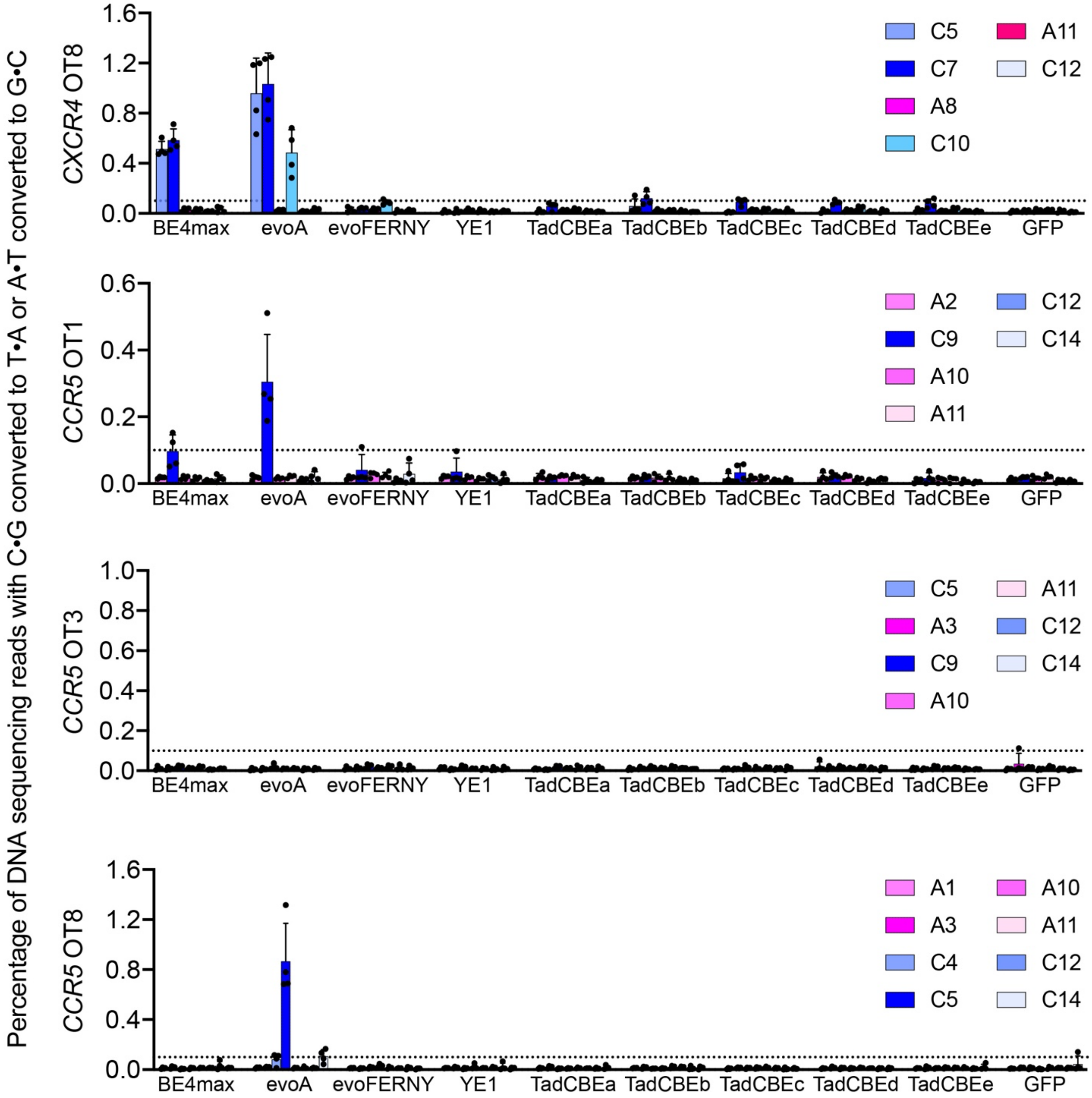

Supplementary Figure 38. **Cas-dependent off-target editing in T-cell experiments targeting *CXCR4* and *CCR5.*** mRNA encoding the indicated base editor or GFP as a negative control was electroporated into primary human T cells (n=4 donors) along with two synthetic guide RNAs targeting **(a**) *CXCR4* or **(b)** *CCR5* at the specified protospacers. After 3 days, genomic DNA was harvested from T-cell lysates and known off-target sites were amplified using the primers in Supplementary Table 4. C•G-to-T•A base editing is shown in shades of blue. A•T-to-G•C base editing is shown in shades of magenta. Dots represent individual values and bars represent mean±s.d. of three independent biological replicates.

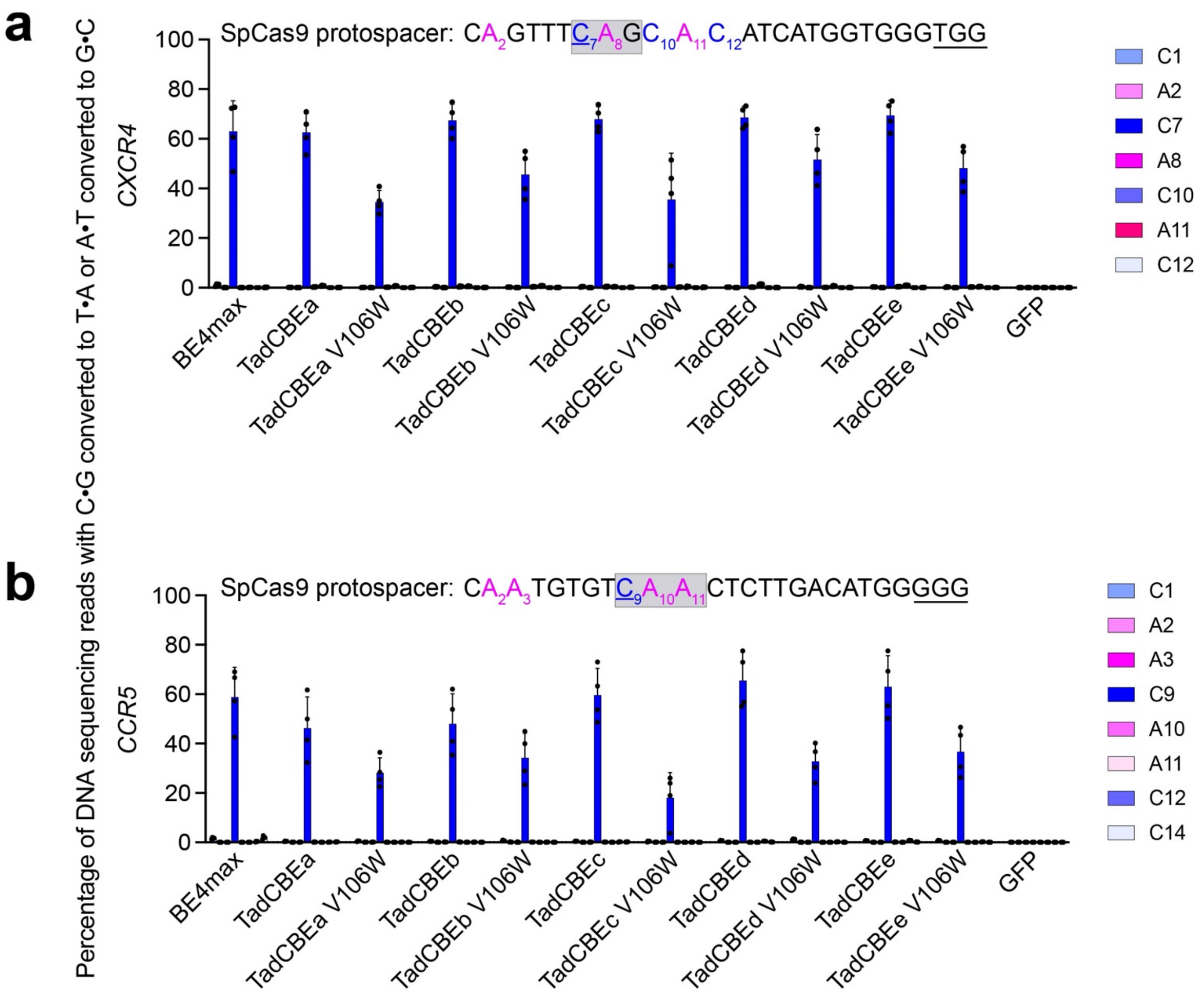

Supplementary Figure 39. On-target editing of V106W variants for T-cell experiments targeting *CXCR4* and *CCR5*. mRNA encoding the indicated base editor or GFP as a negative control was electroporated into human T cells (n=4 donors) along with two synthetic guide RNAs targeting **(a**) *CXCR4* or **(b)** *CCR5* at the specified protospacers. Target cytosines are blue, target adenines are magenta, and PAM sequences are underlined. After 3 days, genomic DNA was harvested from T-cell lysates and analyzed by high-throughput sequencing. The grey boxes indicate the desired location of stop codon installation in *CXCR4* and *CCR5*. The targeted cytidine to yield TAG (*CXCR4*) and TAA (*CCR5*) stop codons upon cytosine base editing is underlined. Dots represent individual values and bars represent mean±s.d. of three independent biological replicates.

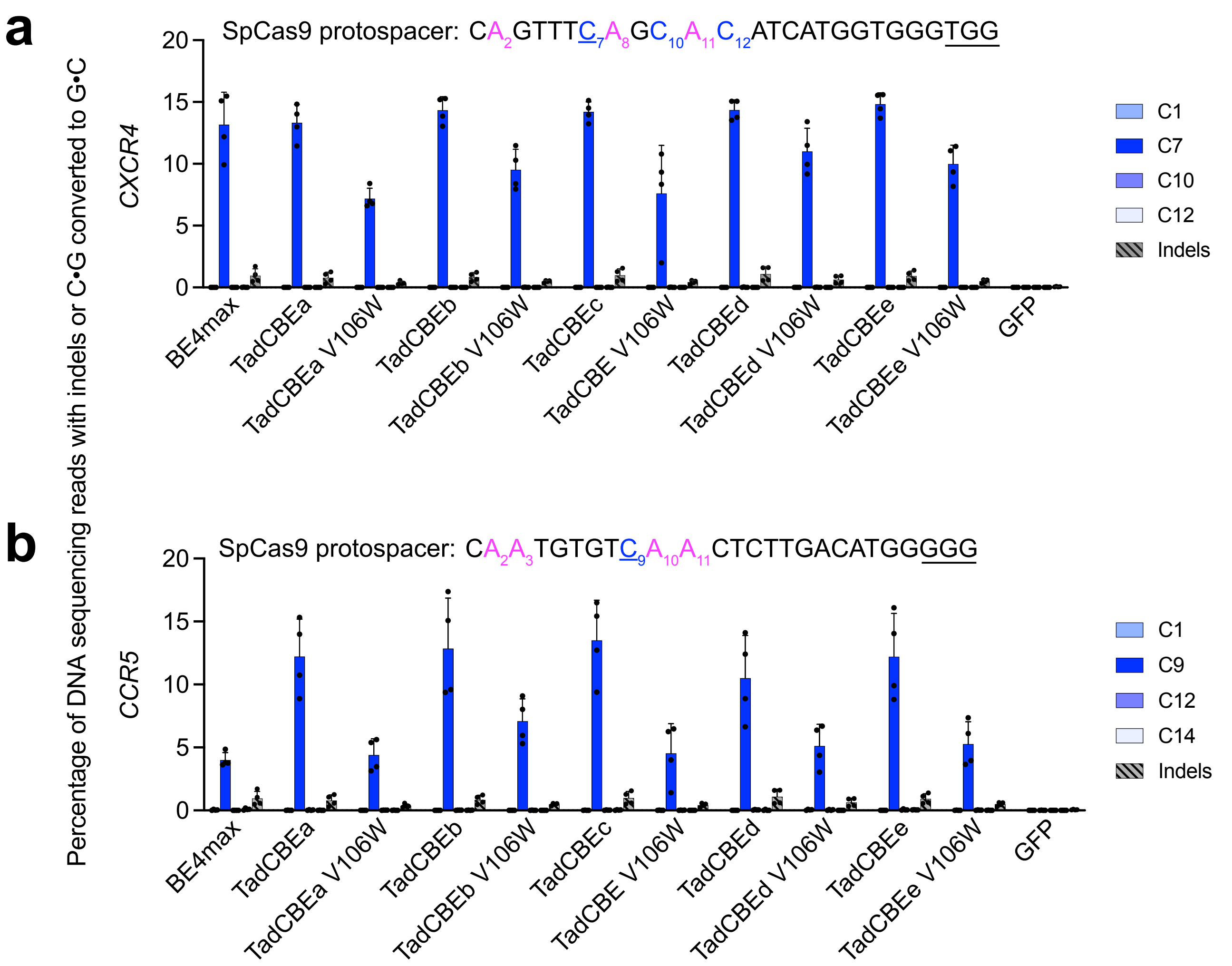

Supplementary Figure 40. C•G-to-G•C editing and indels for T-cell experiments targeting *CXCR4* and *CCR5* with TadCBEe V106W variants. mRNA encoding the indicated base editor or GFP as a negative control was electroporated into primary human T cells (n=4 donors) along with two synthetic guide RNAs targeting (**a**) *CXCR4* or **(b)** *CCR5* at the specified protospacers. After 3 days, genomic DNA was harvested from T-cell lysates and analyzed by high-throughput sequencing. C•G-to-G•C base editing is shown in shades of blue. Indels are shown in grey. Dots represent individual values and bars represent mean±s.d. of three independent biological replicates.

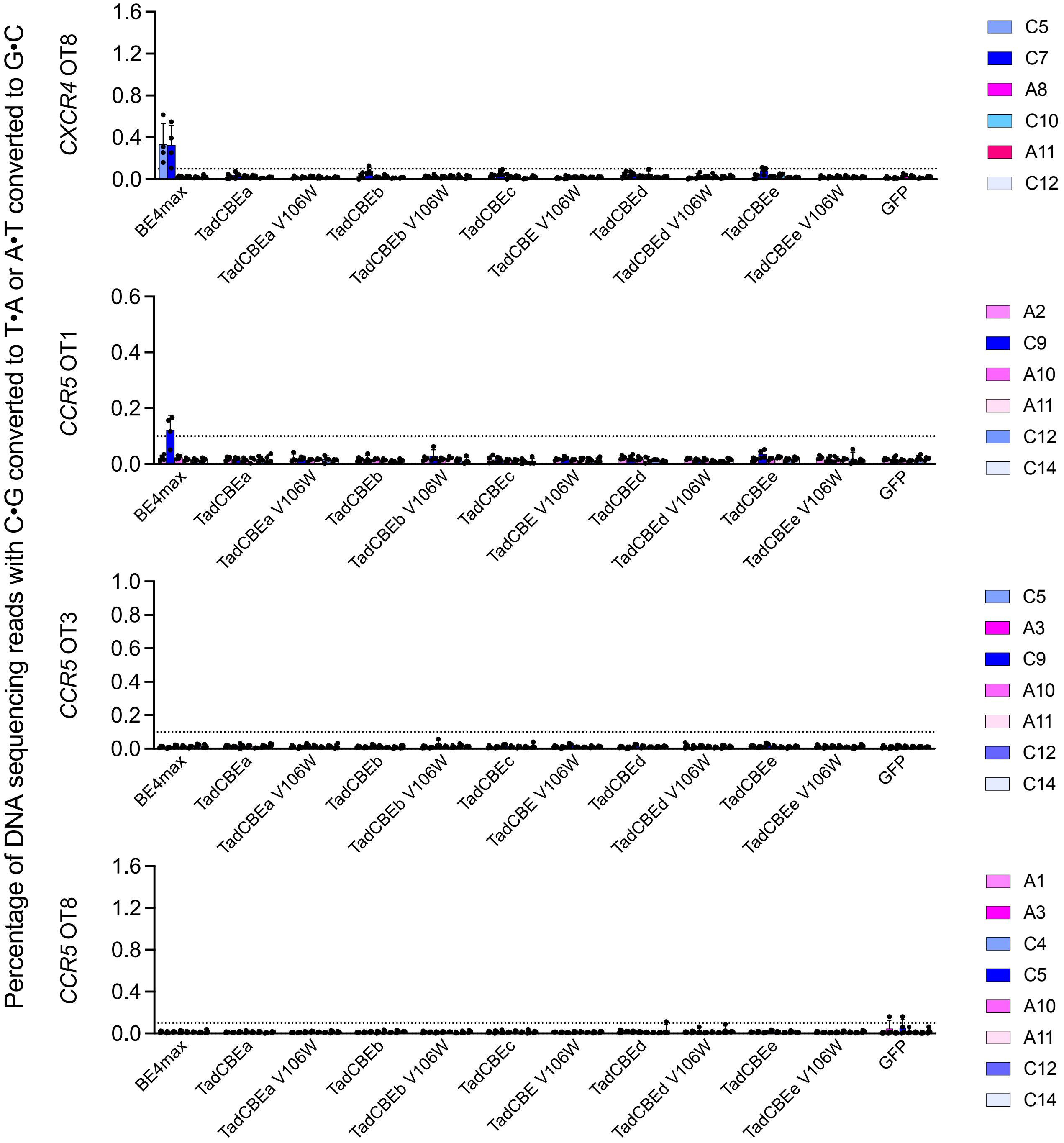

Supplementary Figure 41. **Cas-dependent off-target editing in T-cell experiments targeting *CXCR4* and *CCR5* with TadCBEe V106W variants.** mRNA encoding the indicated base editor or GFP as a negative control was electroporated into primary human T cells (n=4 donors) along with two synthetic guide RNAs targeting **(a**) *CXCR4* or **(b)** *CCR5* at the specified protospacers. After 3 days, genomic DNA was harvested from T-cell lysates and known off-target sites were amplified using the primers in Supplementary Table 4. C•G-to-T•A base editing is shown in shades of blue. A•T-to-G•C base editing is shown in shades of magenta. Dots represent individual values and bars represent mean±s.d. of three independent biological replicates.

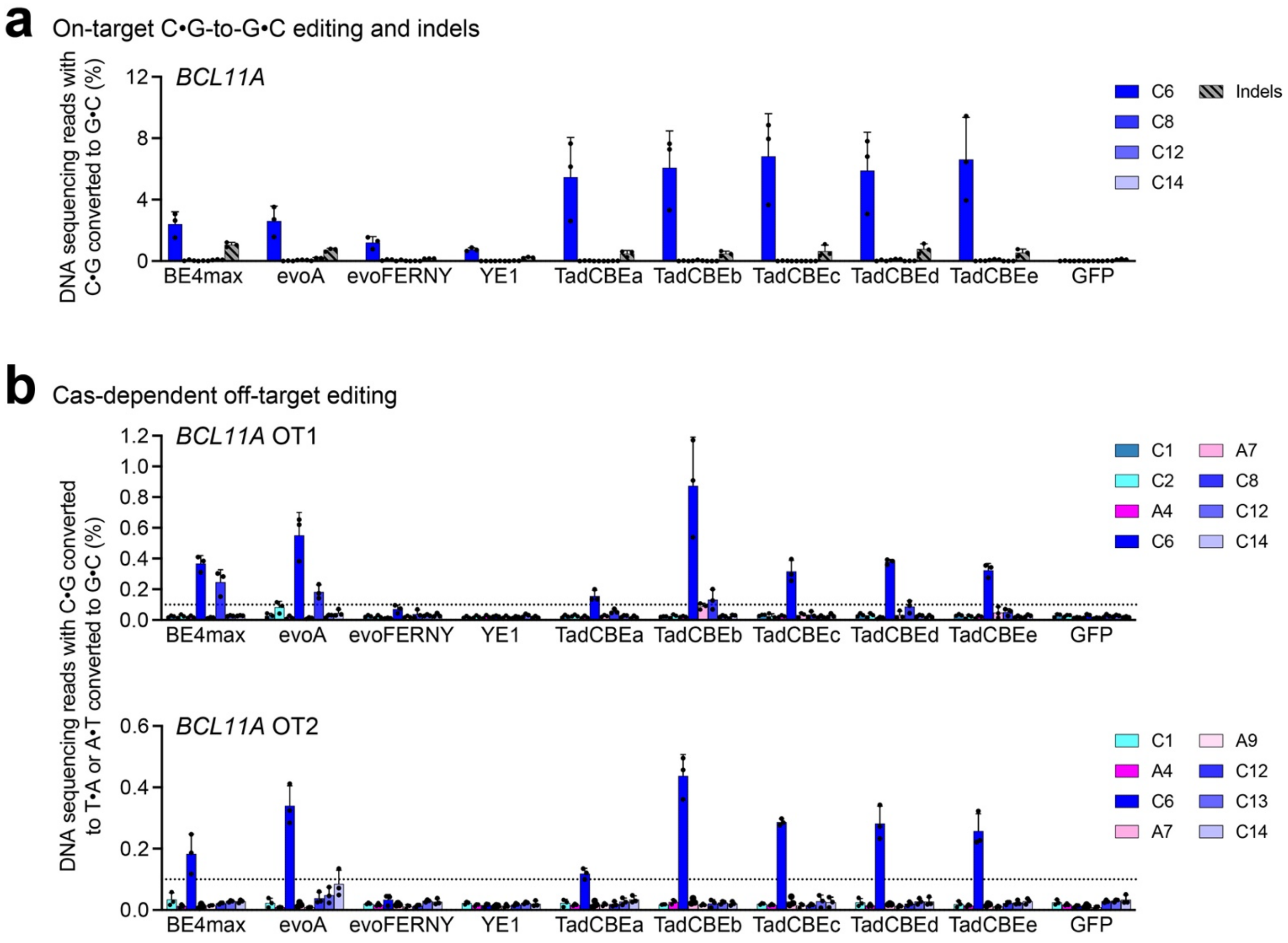

Supplementary Figure 42. C•G-to-G•C editing, indels, and Cas-dependent off-target editing for editing of *BCL11A* in hematopoietic stem and progenitor cells. mRNA encoding the indicated base editor or GFP as a negative control was electroporated into CD34-positive human hematopoietic stem and progenitor cells (n=3 donors) along a synthetic guide RNA targeting *BCL11A* at the specified protospacer. After 3 days, genomic DNA was harvested from cell lysates and analyzed by high-throughput sequencing. **(a)** C•G-to-G•C base editing is shown in shades of blue. Indels are shown in grey. **b)** Known Cas-dependent off-target sites were amplified by the primers listed in Supplementary Table 4. C•G-to-G•C base editing is shown in shades of blue. A•T-to-G•C base editing is shown in shades of magenta. Dots represent individual values and bars represent mean±s.d. of three independent biological replicates.

Supplementary Table 1. Selectivity of TadCBEs and TadDE calculated from the mESC library experiment. Selectivity is defined as the geometric mean of (the ratio of (average CBE editing at each position) to (average ABE editing at each position)) for bases in the 30% window. P(ABE|CBE) is the average probability of observing A•T-to-G•C editing in a read given that C•G-to-G•C editing was observed.

Variant	Selectivity	P (ABE|CBE) rep1	P (ABE|CBE) rep2
BE4max	>100	0.01	0.00
TadCBEa	18.19	0.10	0.11
TadCBEb	10.98	0.17	0.18
TadCBEc	13.84	0.12	0.14
TadCBEd	26.87	0.09	0.09
TadCBEe	15.72	0.12	0.13
TadCBEa V106W	31.62	0.06	0.05
TadCBEb V106W	19.91	0.08	0.09
TadCBEc V106W	28.80	0.06	0.06
TadCBEd V106W	47.80	0.04	0.05
TadDE	1.00	0.62	0.63

Supplementary Table 2. Plasmids and selection phage (SP) used in this work

Name	Usage (resistance)	Origin	ORF1	ORF2
pBT44c	Circuits 1 and 2 (KmR)	p15a	proC.SD8.intC.dCas9.UGI	
pMN404	Circuit 1 (CbR)	SC101-E93K	proT7.sd8.gIII.luxAB	proLac.sgRNA [Circuit 1]
pMN405	Circuit 1 (SpR)	ColE1	proA.SynRBS 0.4k.T7-RNAP-degron	
pMN406	Circuit 1 (SpR)	ColE1	proB.SynRBS 0.4k.T7-RNAP-degron	
pMN407	Circuit 1 (SpR)	ColE1	proC.SynRBS 0.4k.T7-RNAP-degron	
pMN408	Circuit 1 (SpR)	ColE1	proD.SynRBS 0.4k.T7-RNAP-degron	
pBT120b	Circuit 2 (CbR)	SC101-E93K	proT7.sd8.gIII.luxAB	proLac.sgRNA [Circuit 2]
pBT2dR3-ProA	Circuit 2 (SpR)	ColE1	proA.SynRBS 0.4k.T7-RNAP-degron	
pBT2dR3-ProB	Circuit 2 (SpR)	ColE1	proB.SynRBS 0.4k.T7-RNAP-degron	
pBT2dR3-ProC	Circuit 2 (SpR)	ColE1	proC.SynRBS 0.4k.T7-RNAP-degron	
pBT2dR3-ProD	Circuit 2 (SpR)	ColE1	proD.SynRBS 0.4k.T7-RNAP-degron	
pMN618	E. coli editing (CbR)	SC101	pBAD.SD8.EvoAPOBEC1-8e.dSpCas9-UGI-UGI	proLac.sgRNA [Circuit 2]
pMN619	E. coli editing (CbR)	SC101	pBAD.SD8.TadA-8e.dSpCas9-UGI-UGI	proLac.sgRNA [Circuit 2]
pMN620	E. coli editing (CbR)	SC101	pBAD.SD8.TadA-CDa.dSpCas9-UGI-UGI	proLac.sgRNA [Circuit 2]
pMN621	E. coli editing (CbR)	SC101	pBAD.SD8.TadA-CDb.dSpCas9-UGI-UGI	proLac.sgRNA [Circuit 2]
pMN622	E. coli editing (CbR)	SC101	pBAD.SD8.TadA-CDc.dSpCas9-UGI-UGI	proLac.sgRNA [Circuit 2]
pMN415	E. coli editing target (KmR)	RSF1030	Target: ACTTCGCGTTCGCCTGGACC, PAM=AGG	
pMN573	BE4max IVT template	pUC	proT7 (mutated).BE4max	
pMN574	EvoFERNY IVT template	pUC	proT7 (mutated).EvoFERNY	
pMN575	EvoAPOBEC IVT template	pUC	proT7 (mutated).EvoA	
pMN576	TadCBEa IVT template	pUC	proT7 (mutated).TadCBEa	
pMN577	TadCBEb IVT template	pUC	proT7 (mutated).TadCBEb	
pMN578	TadCBEc IVT template	pUC	proT7 (mutated).TadCBEc	
pMN579	TadCBEd IVT template	pUC	proT7 (mutated).TadCBEd	
pMN580	TadCBEe IVT template	pUC	proT7 (mutated).TadCBEe	
pMN582	YE1 IVT template	pUC	proT7 (mutated).YE1	
pMN731	TadCBEa V106W IVT template	pUC	proT7 (mutated).TadCBEa	
pMN732	TadCBEb V106W IVT template	pUC	proT7 (mutated).TadCBEb	
pMN733	TadCBEc V106W IVT template	pUC	proT7 (mutated).TadCBEc	
pMN734	TadCBEd V106W IVT template	pUC	proT7 (mutated).TadCBEd	
pMN735	TadCBEe V106W IVT template	pUC	proT7 (mutated).TadCBEe	
pMN446	Mammalian expression, SpCas9	pUC	pCMV.TadCBEa	
pMN447	Mammalian expression, SpCas9	pUC	pCMV.TadCBEb	
pMN448	Mammalian expression, SpCas9	pUC	pCMV.TadCBEc	
pMN450	Mammalian expression, SpCas9	pUC	pCMV.TadCBEd	
pMN584	Mammalian expression, SpCas9	pUC	pCMV.TadCBEe	
pMN554	Mammalian expression, SpCas9	pUC	pCMV.ABE8e V106W-UGI-UGI	
pMN556	Mammalian expression, SpCas9	pUC	pCMV.TadCBEa V106W	
pMN557	Mammalian expression, SpCas9	pUC	pCMV.TadCBEb V106W	

pMN558	Mammalian expression, SpCas9	pUC	pCMV.TadCBEc V106W	
pMN560	Mammalian expression, SpCas9	pUC	pCMV.TadCBEd V106W	
pMN585	Mammalian expression, SpCas9	pUC	pCMV.TadCBEe V106W	
pMN597	Mammalian expression, SpCas9	pUC	pCMV.TadDE	
pMN599	Mammalian expression, eNme2-C S6P	pUC	pCMV.ABE8e-UGI-UGI	
pMN607	Mammalian expression, eNme2-C S6P	pUC	pCMV.BE4max (eNme2-C S6P)	
pMN608	Mammalian expression, eNme2-C S6P	pUC	pCMV.EvoFERNY (eNme2-C S6P)	
pMN609	Mammalian expression, eNme2-C S6P	pUC	pCMV.EvoA (eNme2-C S6P)	
pMN601	Mammalian expression, eNme2-C S6P	pUC	pCMV.TadCBEa (eNme2-C S6P)	
pMN602	Mammalian expression, eNme2-C S6P	pUC	pCMV.TadCBEb (eNme2-C S6P)	
pMN603	Mammalian expression, eNme2-C S6P	pUC	pCMV.TadCBEc (eNme2-C S6P)	
pMN605	Mammalian expression, eNme2-C S6P	pUC	pCMV.TadCBEd (eNme2-C S6P)	
pMN606	Mammalian expression, eNme2-C S6P	pUC	pCMV.TadCBEe (eNme2-C S6P)	
pMN709	Mammalian expression, eNme2-C S6P	pUC	pCMV.TadDE (eNme2-C S6P)	
pMN745	Mammalian expression, SaCas9	pUC	pCMV.BE4max (SaCas9)	
pMN746	Mammalian expression, SaCas9	pUC	pCMV.evoA (SaCas9)	
pMN747	Mammalian expression, SaCas9	pUC	pCMV.evoFERNY (SaCas9)	
pMN748	Mammalian expression, SaCas9	pUC	pCMV.ABE8e-UGI-UGI (SaCas9)	
pMN749	Mammalian expression, SaCas9	pUC	pCMV.TadCBEa (SaCas9)	
pMN750	Mammalian expression, SaCas9	pUC	pCMV.TadCBEb (SaCas9)	
pMN751	Mammalian expression, SaCas9	pUC	pCMV.TadCBEc (SaCas9)	
pMN752	Mammalian expression, SaCas9	pUC	pCMV.TadCBEd (SaCas9)	
pMN753	Mammalian expression, SaCas9	pUC	pCMV.TadCBEe (SaCas9)	
pMN754	Mammalian expression, SaCas9	pUC	pCMV.TadDE (SaCas9)	
pMN589	Mammalian expression, SpCas9	pUC	pCMV.ABE8e E59A-UGI-UGI	
pMN739	Mammalian expression, SpCas9 library analysis	pUC	p2T-CMV.BE4max.BlastR	
pMN717	Mammalian expression, SpCas9 library analysis	pUC	p2T-CMV.TadCBEa.BlastR	
pMN718	Mammalian expression, SpCas9 library analysis	pUC	p2T-CMV.TadCBEb.BlastR	
pMN719	Mammalian expression, SpCas9 library analysis	pUC	p2T-CMV.TadCBEc.BlastR	
pMN720	Mammalian expression, SpCas9 library analysis	pUC	p2T-CMV.TadCBEd.BlastR	
pMN721	Mammalian expression, SpCas9 library analysis	pUC	p2T-CMV.TadCBEe.BlastR	
pMN723	Mammalian expression, SpCas9 library analysis	pUC	p2T-CMV.TadCBEa V106W.BlastR	
pMN724	Mammalian expression, SpCas9 library analysis	pUC	p2T-CMV.TadCBEb V106W.BlastR	
pMN725	Mammalian expression, SpCas9 library analysis	pUC	p2T-CMV.TadCBEc V106W.BlastR	
pMN726	Mammalian expression, SpCas9 library analysis	pUC	p2T-CMV.TadCBEd V106W.BlastR	

SPMN21	PA(N)CE, ΔgIII SP	M13 f1	p_gIII.SD4.TadA-8e-NpuN	

Supplementary Table 3. Promoter and RBS sequences used in this study.

Name	Promoter/RBS	Description	Sequence
SP	PgIII	Promoter for TadA*-NpuN on phage	AATTCACCTCGAAAGCAAGTTGATAAACTGATACAATTAAAGGCTCCT
SP	RBS	RBS for expression of TadA*-NpuN on phage	AAGGAGGAAAA
P1	ProC	Promoter for NpuC-dCas9-ugi	CACAGCTAACACCACGTCGTCCCTATCTGCTGCCCTAGGTCTATGAGTGGTTGCTGGATAACTTTACGGGCATGCATAAGGCTCGTATGATATATTCAGGGAGACCACAACGGTTTCCCTCTACAAATAATTTTGTTTAACTTTTACTAGAGTGGGACCCTACCTGCAGGTGCAGT
P1	SD8	RBS for expression of NpuC-dCas9-ugi	AAGGAGGAAAAAAAA
P2	pLac	Promoter for sgRNA	GGCTTTACACTTTATGCTTCCGGCTCGTATGTTGTGTGG
P2	proT7	T7 promoter controls gIII transcription	TAATACGACTCACTATAGGGAGA
P2	sd8	RBS for expression of gIII	AAGGAAAAAAAAA
P3	ProA	Promoter for T7RNAP. Varies by stringency (A is weakest, most stringent)	CACAGCTAACACCACGTCGTCCCTATCTGCTGCCCTAGGTCTATGAGTGGTTGCTGGATAACTTTACGGGCATGCATAAGGCTCGTAGGCTATATTCAGGGAGACCACAACGGTTTCCCTCTACAAATAATTTTGTTTAACTTTTACTAGAGTGGGACCCTACCTGCAGGTGCAGT
P3	ProB	Promoter for T7RNAP. Varies by stringency.	CACAGCTAACACCACGTCGTCCCTATCTGCTGCCCTAGGTCTATGAGTGGTTGCTGGATAACTTTACGGGCATGCATAAGGCTCGTAATATATATTCAGGGAGACCACAACGGTTTCCCTCTACAAATAATTTTGTTTAACTTTTACTAGAGTGGGACCCTACCTGCAGGTGCAGT
P3	ProC	Promoter for T7RNAP. Varies by stringency.	CACAGCTAACACCACGTCGTCCCTATCTGCTGCCCTAGGTCTATGAGTGGTTGCTGGATAACTTTACGGGCATGCATAAGGCTCGTATGATATATTCAGGGAGACCACAACGGTTTCCCTCTACAAATAATTTTGTTTAACTTTTACTAGAGTGGGACCCTACCTGCAGGTGCAGT
P3	ProD	Promoter for T7RNAP. Varies by stringency (D is strongest, least stringent)	CACAGCTAACACCACGTCGTCCCTATCTGCTGCCCTAGGTCTATGAGTGGTTGCTGGATAACTTTACGGGCATGCATAAGGCTCGTATAATATATTCAGGGAGACCACAACGGTTTCCCTCTACAAATAATTTTGTTTAACTTTTACTAGAGTGGGACCCTACCTGCAGGTGCAGT
P3	RBS_{R3}	RBS for expression of T7 RNAP	ACTACATCATCAGGC

Supplementary Table 4. Target protospacers and amplicons used in this study with corresponding primers used for genomic DNA amplification.

SpCas9 genomic loci:

Site Name	Protospacer	PAM	Amplicon	HTS-F	HTS-R
FANCF	GGAATCCCTTCTGCAGCACC	TGG	CATTGCAGAGAGGCGTATCATTTCGCGGATGTTCCAATCAGTACGCAGAGAGTCGCCGTCTCCAAGGTGAAAGCGGAAGTAGGGCCTTCGCGCACCTCATGGAATCCCTTCTGCAGCACCTGGATCGCTTTTCCGAGCTTCTGGCGGTCTCAAGCACTACCTACGTCAGCACCTGGGACCCC	ACACTCTTTCCCTACACGACGCTCTTCCGATCTNNNNCATTGCAGAGAGGCGTATCA	TGGAGTTCAGACGTGTGCTCTTCCGATCTGGGGTCCCAGGTGCTGAC
EMX1	GAGTCCGAGCAGAAGAAGAA	GGG	CAGCTCAGCCTGAGTGTTGAGGCCCCAGTGGCTGCTCTGGGGGCCTCCTGAGTTTCTCATCTGTGCCCCTCCCTCCCTGGCCCAGGTGAAGGTGTGGTTCCAGAACCGGAGGACAAAGTACAAACGGCAGAAGCTGGAGGAGGAAGGGCCTGAGTCCGAGCAGAAGAAGAAGGGCTCCCATCACATCAACCGGTGGCGCATTGCCACGAAGCAGGCCAATGGGGAGGACATCGATGTCACCTCCAATGACTAGGGTGGGCAACCACAAACCCACGAG	ACACTCTTTCCCTACACGACGCTCTTCCGATCTNNNNCAGCTCAGCCTGAGTGTTGA	TGGAGTTCAGACGTGTGCTCTTCCGATCTCTCGTGGGTTTGTGGTTGC
BCL11A	TTTATCACAGGCTCCAGGAA	GGG	GCCAGAAAAGAGATATGGCATCTACTCTTAGACATAACACACCAGGGTCAATACAACTTTGAAGCTAGTCTAGTGCAAGCTAACAGTTGCTTTTATCACAGGCTCCAGGAAGGGTTTGGCCTCTGATTAGGGTGGGGGCGTGGGTGGGGTAGAAGAGGACTGGCAGACCTCTCCATCGGTGGCCGTTTGCCCAGGGGGGCCTCTTTCGGAAGGCTCTCT	ACACTCTTTCCCTACACGACGCTCTTCCGATCTNNNNGCCAGAAAAGAGATATGGCATC	TGGAGTTCAGACGTGTGCTCTTCCGATCTAGAGAGCCTTCCGAAAGAGG
HEK3	GGCCCAGACTGAGCACGTGA	TGG	ATGTGGGCTGCCTAGAAAGGCATGGATGAGAGAAGCCTGGAGACAGGGATCCCAGGGAAACGCCCATGCAATTAGTCTATTTCTGCTGCAAGTAAGCATGCATTTGTAGGCTTGATGCTTTTTTTCTGCTTCTCCAGCCCTGGCCTGGGTCAATCCTTGGGGCCCAGACTGAGCACGTGATGGCAGAGGAAAGGAAGCCCTGCTTCCTCCAGAGGGCGTCGCAGGACAGCTTTTCCTAGACAGGGGCTAGTATGTGCAGCTCCTGCACCGGGATACTGGTTGACAAGTTTGGCTGGG	ACACTCTTTCCCTACACGACGCTCTTCCGATCTNNNNATGTGGGCTGCCTAGAAAGG	TGGAGTTCAGACGTGTGCTCTTCCGATCTCCCAGCCAAACTTGTCAACC
B2M	ACTCACGCTGGATAGCCTCC	AGG	GGCGGGCATTCCTGAAGCTGACAGCATTCGGGCCGAGATGTCTCGCTCCGTGGCCTTAGCTGTGCTCGCGCTACTCTCTCTTTCTGGCCTGGAGGCTATCCAGCGTGAGTCTCTCCTACCCTCCCGCTCTGGTCCTTCCTCTCCCGCTCTGCACCCTCTGTGGCCCTCGCTGTGCTCTCTCGCTCCGTGACTTCCCTTCTCCAAGT	ACACTCTTTCCCTACACGACGCTCTTCCGATCTNNNNGGCGGGCATTCCTGAAGCTGA	TGGAGTTCAGACGTGTGCTCTTCCGATCTACTTGGAGAAGGGAAGTCACGGA
RNF2	GTCATCTTAGTCATTACCTG	AGG	ACGTCTCATATGCCCCTTGGCAGTCATCTTAGTCATTACCTGAGGTGTTCGTTGTAACTCATATAAACTGAGTTCCCATGTTTTGCTTAATGGTTGAGTTCCGTTTGTCTGCACAGCCTGAGACATTGCTGGAAATAAAGAAGAGAGAAAAACAATTTTAGTATTTGGAAGGGAAGTGCTATGGTCTGAATGTATGTGTCCCACCAAAATTCCTACGT	ACACTCTTTCCCTACACGACGCTCTTCCGATCTNNNNACGTCTCATATGCCCCTTGG	TGGAGTTCAGACGTGTGCTCTTCCGATCTACGTAGGAATTTTGGTGGGACA
HEK4	GGCACTGCGGCTGGAGGTGG	GGG	GAACCCAGGTAGCCAGAGACCCGCTGGTCTTCTTTCCCCTCCCCTGCCCTCCCCTCCCTTCAAGATGGCTGACAAAGGCCGGGCTGGGTGGAAGGAAGGGAGGAAGGGCGAGGCAGAGGGTCCAAAGCAGGATGACAGGCAGGGGCACCGCGGCGCCCCGGTGGCACTGCGGCTGGAGGTGGGGGTTAAAGCGGAGACTCTGGTGCTGTGTGACTACAGTGGGGGCCCTGCCCTCTCTGAGCCCCCGCCTCCAGGCCTGTGTGTGTGTGTCTCCGTTCGGGTTGAAAGGA	ACACTCTTTCCCTACACGACGCTCTTCCGATCTNNNNGAACCCAGGTAGCCAGAGAC	TGGAGTTCAGACGTGTGCTCTTCCGATCTTCCTTTCAACCCGAACGGAG

PDCD1	CACCTACCTAAGAACCATCC	TGG	GACCTGCCAGGGACTGAGGGTGGAAGGTCCCTCCAGACCCCTGGCTCTGGGACACCTGACCGCCGACCCCACCTACCTAAGAACCATCCTGGCCGCCAGCCCAGTTGTAGCACCGCCCAGACGACTGGCCAGGGCGCCTGTGGGATCTGCATGCCTGGAGCAGCCCCACCAGAGTGCCGCCTTCTC	ACACTCTTTCCCTACACGACGCTCTTCCGATCTNNNNGACCTGCCAGGGACTGAGGG	TGGAGTTCAGACGTGTGCTCTTCCGATCTGAGAAGGCGGCACTCTGGTG
TRAC	TTCGTATCTGTAAAACCAAG	AGG	TCTGCCTTGGGGAAAACCGTGGGTGTGTCCTGCAGGCCATGCAGGCCTGGGACATGCAAGCCCATAACCGCTGTGGCCTCTTGGTTTTACAGATACGAACCTAAACTTTCAAAACCTGTCAGTGATTGGGTTCCGAATCCTCCTCCTGAAAGTGGCCGGGTTTAATCTGCTCATGACGCTGCG	ACACTCTTTCCCTACACGACGCTCTTCCGATCTNNNNTCTGCCTTGGGGAAAACCGT	TGGAGTTCAGACGTGTGCTCTTCCGATCTCGCAGCGTCATGAGCAGATT
R5-7	CAATGTGTCAACTCTTGACA	GGG	GCTGGTCATCCTCATCCTGATAAACTGCAAAAGGCTGAAGAGCATGACTGACATCTACCTGCTCAACCTGGCCATCTCTGACCTGTTTTTCCTTCTTACTGTCCCCTTCTGGGCTCACTATGCTGCCGCCCAGTGGGACTTTGGAAATACAATGTGTCAACTCTTGACAGGGCTCTATTTTATAGGCTTCTTCTCTGGAATCTTCTTCATCATCCTCCTGACAATCGATAGGTACCTGGCTGTCGTCCATGCTGTGTTTGCTTTAAAAGCCAGGACGGTCACCTTTGGGGTGGTGACAAGTGTGATCACTTGGGTGGTGGCTGTGTTTGCGTCTCTCCCAGGAA	ACACTCTTTCCCTACACGACGCTCTTCCGATCTNNNNGCTGGTCATCCTCATCCTG	TGGAGTTCAGACGTGTGCTCTTCCGATCTTTCCTGGGAGAGACGCAAAC
X4-2	CAGTTTCAGCACATCATGGT	TGG	AAGCCAGGATGAGGATGACTGTGGTCTTGAGGGCCTTGCGCTTCTGGTGGCCCTTGGAGTGTGACAGCTTGGAGATGATAATGCAATAGCAGGACAGGATGACAATACCAGGCAGGATAAGGCCAACCATGATGTGCTGAAACTGGAACACAACCACCCACAAGTCATTGGGGTAGAAGCGGTCACAGATATATCTGTCATCTGCCTCACTGACGTTGGCAAAGATGAAGTCGGGAATAGTCAGCAGGAGGGCAGG	ACACTCTTTCCCTACACGACGCTCTTCCGATCTNNNNAAGCCAGGATGAGGATGACTG	TGGAGTTCAGACGTGTGCTCTTCCGATCTCCTGCCCTCCTGCTGACTAT
Circuit 2	GGTCCAGGCGAACGCGAAGT	CGG	GCGCTCGACACCGATATCATAAACATTCTGTTTGCCTAAATATTTACGCGTGGCAATTTTGATTGAGCCACCTTCTGCCCTGGTCCAGGCGAACGCGAAGTCGGATCCTCCGGAATTCGGGAGATTATCCACGCGCATCAGGTCTAATTCGCGTTCAAAAATCTCATCAATGGGGAGCATCTGACCATCTACAGTCATAAATTTGTGATCCTTTGTGGCACGGATTAAAGATCCGTCTTCCAGACAATATTCGAACACTTC	ACACTCTTTCCCTACACGACGCTCTTCCGATCTNNNNGAAGTGTTCGAATATTGTCTGGAAGA	TGGAGTTCAGACGTGTGCTCTTCCGATCTGCGCTCGACACCGATATC

eNme2-C genomic loci:

Site Name	Protospacer	PAM	Amplicon	HTS-F	HTS-R
TS90	GCAAGAGCACAAGAGGAAGAGAG	AGACCC	GCTCAGAAAAAGGGCCCTGACAACTCTTTTCATCTTCTAGGTATGACAACGAATTTGGCTACAGCAACAGGGTGGTGGACCTCATGGCCCACATGGCCTCCAAGGAGTAAGACCCCTGGACCACCAGCCCCAGCAAGAGCACAAGAGGAAGAGAGAGACCCTCACTGCTGGGGAGTCCCTGCCACACTCAGTCCCCCACCACACTGAATCTC	ACACTCTTTCCCTACACGACGCTCTTCCGATCTNNNNGCTCAGAAAAAGGGCCCTGA	TGGAGTTCAGACGTGTGCTCTTCCGATCTGAGATTCAGTGTGGTGGGGG
eNme2-C site 5	GGCTACAGCAACAGGGTGGTGG	ACCTCA	GCTCAGAAAAAGGGCCCTGACAACTCTTTTCATCTTCTAGGTATGACAACGAATTTGGCTACAGCAACAGGGTGGTGGACCTCATGGCCCACATGGCCTCCAAGGAGTAAGACCCCTGGACCACCAGCCCCAGCAAGAGCACAAGAGGAAGAGAGAGACCCTCACTGCTGGGGAGTCCCTGCCACACTCAGTCCCCCACCACACTGAATCTC	ACACTCTTTCCCTACACGACGCTCTTCCGATCTNNNNGCTCAGAAAAAGGGCCCTGA	TGGAGTTCAGACGTGTGCTCTTCCGATCTGAGATTCAGTGTGGTGGGGG
eNme2-C site 66	GGTGTGCAGACGGCAGTCACTAG	GGGGCG	CATTCCCTCTTTAGCCAGAGCCGGGGTGTGCAGACGGCAGTCACTAGGGGGCGCTCGGCCACCACAGGGAAGCTGGGTGAATGGAGCGAGCAGCGTCTTCGAGAGTGAGGACGTGTGTGTCTGTGTGGGTGAGTGAGTGTGTGCGTGTGGGGTTGAGGGCGTTGGAGCGGGGAGAAGGCCAGGGGTCACTCCAGGATTCCAATAGATCTG	ACACTCTTTCCCTACACGACGCTCTTCCGATCTNNNNCATTCCCTCTTTAGCCAGAGCCGG	TGGAGTTCAGACGTGTGCTCTTCCGATCTCAGATCTATTGGAATCCTGGAGTGACC
eNme2-C site 50	GTCTCCGCTTTAACCCCCACCTC	CAGCCG	GGCGAGGCAGAGGGTCCAAAGCAGGATGACAGGCAGGGGCACCGCGGCGCCCCGGTGGCACTGCGGCTGGAGGTGGGGGTTAAAGCGGAGACTCTGGTGCTGTGTGACTACAGTGGGGGCCCTGCCCTCTCTGAGCCCCCGCCTCCAGGCCTGTGTGTGTGTCTCCGTTCGGGTTGAAAGGAGCCCGGGAAAAAGGCCCCAGAAGGAG	ACACTCTTTCCCTACACGACGCTCTTCCGATCTNNNNGGCGAGGCAGAGGGTCCAAA	TGGAGTTCAGACGTGTGCTCTTCCGATCTCTCCTTCTGGGGCCTTTTTCCC
eNme2-C site 39	CCTCTTGCCTCCACTGGTTGTGC	GCGGCT	CTACCTGCGCCACATCCATCGGCGCTTTGGTCGGCATGGCCCCATTCGCACGGCTCTGGAGCGGCGGCTGCACAACCAGTGGAGGCAAGAGGGCGGCTTTGGGCGGGGTCCAGTTCCGGGATTAGCGAACTTCCAGGCCCTCGGTCACTGTGACGTCCTGCTCTCTCTGCGCCTGCTGGAGAACCGGGCCCTCGGGGATGCAGCTCGTTACCACCTGGTGCAGCAACTCTTT	ACACTCTTTCCCTACACGACGCTCTTCCGATCTNNNNAAAGAGTTGCTGCACCAGGT	TGGAGTTCAGACGTGTGCTCTTCCGATCTCTACCTGCGCCACATCCATC
eNme2-C site 11	GGTCCAAAGCAGGATGACAGGCA	GGGGCA	GAACCCAGGTAGCCAGAGACCCGCTGGTCTTCTTTCCCCTCCCCTGCCCTCCCCTCCCTTCAAGATGGCTGACAAAGGCCGGGCTGGGTGGAAGGAAGGGAGGAAGGGCGAGGCAGAGGGTCCAAAGCAGGATGACAGGCAGGGGCACCGCGGCGCCCCGGTGGCACTGCGGCTGGAGGTGGGGGTTAAAGCGGAGACTCTGGTGCTGTGTGACTACAGTGGGGGCCCTGCCCTCTCTGAGCCCCCGCCTCCAGGCCTGTGTGTGTGTCTCCGTTCGGGTTGAAAGGA	ACACTCTTTCCCTACACGACGCTCTTCCGATCTNNNNGAACCCAGGTAGCCAGAGAC	TGGAGTTCAGACGTGTGCTCTTCCGATCTTCCTTTCAACCCGAACGGAG

SaCas9 genomic loci:

Site Name	Protospacer	PAM	Amplicon	HTS-F	HTS-R
Sa1	GTGGTAGACAGCATGTGTCCTA	AAGGGT	TGGTGGAGTGCTCTGTGTTTGTCTTTATAAACCCAGATGAGAGGATGAAGGCAACAAGCTTCTGTACCAACATACATGCCCCTTTGCCTCAAGTCTGGTTATTTTAGGGGGATGCTAGGTTGCTTTGGGTCTACCTTACTGAGAAAATGGCCCCAGGTCATTGTCATGTCCAGTTGTGGTAGACAGCATGTGTCCTAAAGGGTATATTCACATGCATGTGCAAAAATACAGGGGTCCTTCTAACCCTATCACAGAGAAGCAGGAGACTGC	ACACTCTTTCCCTACACGACGCTCTTCCGATCTNNNNTGCAGTCTCCTGCTTCTCTG	TGGAGTTCAGACGTGTGCTCTTCCGATTGGTGGAGTGCTCTGTGTTTG
Sa2	ATTTACAGCCTGGCCTTTGGGG	TCGGGT	GCTACAGAAAGGTCAGCAGCTATATTTAACCTCAGACCAGGGTGCGGTGGGAGATCTGGTTTCCGGAAGACGGAATGGGGAGAAGGGCAGGTTCCCCGAGGCGCCCAGACACCCAATCCTCCCGGTGACATTTACAGCCTGGCCTTTGGGGTCGGGTCAACGCTAGGCTGGCAGGGGAAGGGCGGGGCCGTGAGGTGAGCCGGCGCTGCAGGAAGGGGCCACCACCAGAGGGGCCATTTTGCGGTGGAAATGTCC	ACACTCTTTCCCTACACGACGCTCTTCCGATCTNNNNGGACATTTCCACCGCAAAATG	TGGAGTTCAGACGTGTGCTCTTCCGATGCTACAGAAAGGTCAGCAGC
Sa3	GTGTCAGGTAATGTGCTAAACA	GAGAGT	AAGTGTTCAGCTGCTTTTCTTTCATTTATTCCACATATAATTACTATAATTGCTAAACATTTATTTAGTGTCAGGTAATGTGCTAAACAGAGAGTTACTGCTCAGACATGTAATAATAATAAATAACACATCAAATAACCATACCATTTTAAGCTGTAGTATTATGAAGGGAAATCTGGAGCAAAGAGAATAGACTGTAGGGAAACCAGTTAAGAAATAGGACATGGAGGCTAGGTGCAG	ACACTCTTTCCCTACACGACGCTCTTCCGATCTNNNNCTGCACCTAGCCTCCATGTC	TGGAGTTCAGACGTGTGCTCTTCCGATCTGCTGTGGCATCCAGAGACAT
Sa4	TCTGCTTCTCCAGCCCTGGC	CTGGGT	ATGTGGGCTGCCTAGAAAGGCATGGATGAGAGAAGCCTGGAGACAGGGATCCCAGGGAAACGCCCATGCAATTAGTCTATTTCTGCTGCAAGTAAGCATGCATTTGTAGGCTTGATGCTTTTTTTCTGCTTCTCCAGCCCTGGCCTGGGTCAATCCTTGGGGCCCAGACTGAGCACGTGATGGCAGAGGAAAGGAAGCCCTGCTTCCTCCAGAGGGCGTCGCAGGACAGCTTTTCCTAGACAGGGGCTAGTATGTGCAGCTCCTGCACCGGGATACTGGTTGACAAGTTTGGCTGGG	ACACTCTTTCCCTACACGACGCTCTTCCGATCTNNNNATGTGGGCTGCCTAGAAAGG	TGGAGTTCAGACGTGTGCTCTTCCGATCTCCAGCCAAACTTGTCAACC
Sa5	GATGTTCCAATCAGTACGCA	GAGAGT	CATTGCAGAGAGGCGTATCATTTCGCGGATGTTCCAATCAGTACGCAGAGAGTCGCCGTCTCCAAGGTGAAAGCGGAAGTAGGGCCTTCGCGCACCTCATGGAATCCCTTCTGCAGCACCTGGATCGCTTTTCCGAGCTTCTGGCGGTCTCAAGCACTACCTACGTCAGCACCTGGGACCCC	ACACTCTTTCCCTACACGACGCTCTTCCGATCTNNNNCATTGCAGAGAGGCGTATCA	TGGAGTTCAGACGTGTGCTCTTCCGATCTGGGTCCCAGGTGCTGAC
Sa6	GCAAGGCCCGGCGCACGGTGG	CGGGGT	GATCGCTTTTCCGAGCTTCTGGCGGTCTCAAGCACTACCTACGTCAGCACCTGGGACCCCGCCACCGTGCGCCGGGCCTTGCAGTGGGCGCGCTACCTGCGCCACATCCATCGGCGCTTTGGTCGGCATGGCCCCATTCGCACGGCTCTGGAGCGGCGGCTGCACAACCAGTGGAGGCAAGAGGGCGGCTTTGGGCGGGGTCCAGTTCCGGGATTAGCGAACTTCCAGGCCCTCGGTCACTGTGACGTCCTGCTCTCTCTGCGCCTGCTGGAGAACCGGGCCCTCGGGGATGCAGCTCGTTACCACCTGGTGC	ACACTCTTTCCCTACACGACGCTCTTCCGATCTNNNNGATCGCTTTTCCGAGCTTCTGGC	TGGAGTTCAGACGTGTGCTCTTCCGATCTGCACCAGGTGGTAACGAGCTGCATC
Sa7	ACGTGCTCAGTCTGGGCCCC	AAGGAT	ATGTGGGCTGCCTAGAAAGGCATGGATGAGAGAAGCCTGGAGACAGGGATCCCAGGGAAACGCCCATGCAATTAGTCTATTTCTGCTGCA	ACACTCTTTCCCTACACGACGCTC	TGGAGTTCAGACGTGTGC

			AGTAAGCATGCATTTGTAGGCTTGATGCTTTTTTTCTGCTTCTCCAGCCCTGGCCTGGGTCAATCCTTGGGGCCCAGACTGAGCACGTGATGGCAGAGGAAAGGAAGCCCTGCTTCCTCCAGAGGGCGTCGCAGGACAGCTTTTCCTAGACAGGGGCTAGTATGTGCAGCTCCTGCACCGGGATACTGGTTGACAAGTTTGGCTGGG	TTCCGATCTNNNNNATGTGGGCTGCCTAGAAAGG	TCTTCCGATCTCCCAGCCAAACTTGTCAACC
Sa8	GCGAGGCAGAGGGTCCAAAG	CAGGAT	GAACCCAGGTAGCCAGAGACCCGCTGGTCTTCTTTCCCCTCCCCTGCCCTCCCCTCCCTTCAAGATGGCTGACAAAGGCCGGGCTGGGTGGAAGGAAGGGAGGAAGGGCGAGGCAGAGGGTCCAAAGCAGGATGACAGGCAGGGGCACCGCGGCGCCCCGGTGGCACTGCGGCTGGAGGTGGGGGTTAAAGCGGAGACTCTGGTGCTGTGTGACTACAGTGGGGGCCCTGCCCTCTCTGAGCCCCCGCCTCCAGGCCTGTGTGTGTGTCTCCGTTCGGGTTGAAAGGA	ACACTCTTTCCCTACACGACGCTCTTCCGATCTNNNNGAACCCAGGTAGCCAGAGAC	TGGAGTTCAGACGTGTGCTCTTCCGATCTTCCTTTCAACCCGAACGGAG
Sa9	CCACTGTAGTCACACAGCAC	CAGAGT	GAACCCAGGTAGCCAGAGACCCGCTGGTCTTCTTTCCCCTCCCCTGCCCTCCCCTCCCTTCAAGATGGCTGACAAAGGCCGGGCTGGGTGGAAGGAAGGGAGGAAGGGCGAGGCAGAGGGTCCAAAGCAGGATGACAGGCAGGGGCACCGCGGCGCCCCGGTGGCACTGCGGCTGGAGGTGGGGGTTAAAGCGGAGACTCTGGTGCTGTGTGACTACAGTGGGGGCCCTGCCCTCTCTGAGCCCCCGCCTCCAGGCCTGTGTGTGTGTCTCCGTTCGGGTTGAAAGGA	ACACTCTTTCCCTACACGACGCTCTTCCGATCTNNNNGAACCCAGGTAGCCAGAGAC	TGGAGTTCAGACGTGTGCTCTTCCGATCTTCCTTTCAACCCGAACGGAG

SpCas9 Cas-dependent off-target sites:

Site Name	Protospacer	PAM	Amplicon	HTS-F	HTS-R
HEK3OT1	CACCCAGACTGAGCACGTGC	TGG	TCCCCTGTTGACCTGGAGAAGCATGAACCAGTCAAAAAGTTTAAAGACAAGAGCATTAACTGCACCAGTGGGCAGCTCAGCTCAGACACCAGTAGCGTGGGCACCCAGACTGAGCACGTGCTGGAGCCCAAGAAATGCAGAGACCTGTGCACCTCTGGTCAGGGCAAGTACAGTG	ACACTCTTTCCCTACACGACGCTCTTCCGATCTNNNNTCCCCTGTTGACCTGGAGAA	TGGAGTTCAGACGTGTGCTCTTCCGATCTCACTGTACTTGCCCTGACCA
HEK3OT2	GACACAGACTGGGCACGTGA	GGG	TTGGTGTTGACAGGGAGCAACTTCACAGTCCCAGGCATCAGGACACAGACTGGGCACGTGAGGGAAGCCCAAGGGAGAGGACTGGTGTAATCGAGGCTGACTCCACTTTTAATGTTTGACTGATGATAGGTTTCAAGTCTCACTAAGTCTCCTTCCCCTTCTGCCCACATCTCAG	ACACTCTTTCCCTACACGACGCTCTTCCGATCTNNNNTTGGTGTTGACAGGGAGCAA	TGGAGTTCAGACGTGTGCTCTTCCGATCTCTGAGATGTGGGCAGAAGGG
HEK3OT3	AGCTCAGACTGAGCAAGTGA	GGG	TGAGAGGGAACAGAAGGGCTAAGACTAAAAGGAACAGAGGAGTTCATAGTGAGCGGTAAAGAGCTCAGACTGAGCAAGTGAGGGGCTCAGCCTCCCATGGAGGACAGGGGGCTGGGGCCCCTGGCTGATGTCTGGACTGAAGCCCCCACGCCCAGAGGTTCTTGGGCCTTTGGAC	ACACTCTTTCCCTACACGACGCTCTTCCGATCTNNNNTGAGAGGGAACAGAAGGGCT	TGGAGTTCAGACGTGTGCTCTTCCGATCTGTCCAAAGGCCCAAGAACCT
HEK3OT4	AGACCAGACTGAGCAAGAGA	GGG	CCTAGCACTTTGGAAGGTCGAAGCGGCAGGATGGCTTCAACCCAGGAGTTCGAGACCAGACTGAGCAAGAGAGAGGGAGAGTGTCTGTATTAACAACAAACAAACAAACAAAAAACTAAACTAAAAGAAACTGTGGTGTATAATATAAAATTCTGGCTGAGCAGATTAAGATGAGC	ACACTCTTTCCCTACACGACGCTCTTCCGATCTNNNNTCCTAGCACTTTGGAAGGTCG	TGGAGTTCAGACGTGTGCTCTTCCGATCTGCTCATCTTAATCTGCTCAGCC
HEK3OT5	GAGCCAGAATGAGCACGTGA	GGG	AAAGGAGCAGCTCTTCCTGGTGGAAATTGCGAGCAGAGGCTGCGTGAGTTCCGTAACTCGCACACAGCCTCCATTTGGAGCCAGAATGAGCACGTGAGGGACCCCGGGCAGAGGGGCCAGTGCTGACATTATGCTCCATGCAACCTCCCATCCTGTTGTGGGAGATGGTGCAGAC	ACACTCTTTCCCTACACGACGCTCTTCCGATCTNNNNAAAGGAGCAGCTCTTCCTGG	TGGAGTTCAGACGTGTGCTCTTCCGATCTGTCTGCACCATCTCCCACAA
HEK4OT1	TGCACTGCGGCCGGAGGAGG	TGG	GGCATGGCTTCTGAGACTCATAGCTGGGGCTGAAGATCCCTAGGGGGGCTCTGCTGGGCTCACTGCTCTCCAGAGTGGTCCAGCCCGGCTGCAGGGTGCTGCTTCCAGCTTGGTGCACTGCGGCCGGAGGAGGTGGAGGATGGAAAGTAAGATTCAAAGACAGGGAGTGCAAGGG	ACACTCTTTCCCTACACGACGCTCTTCCGATCTNNNNGGCATGGCTTCTGAGACTCA	TGGAGTTCAGACGTGTGCTCTTCCGATCTGTCTCCCTTGCACTCCC

					TGTCTTT
HEK4OT2	GGCTCTGCGGCTGGAGGGGG	TGG	TTTGGCAATGGAGGCATTGGGCAGGGGAAGCCTGTCTTCAGGGCACATGCACGTGCGCAGGGCTCTGCGGCTGGAGGGGGTGGGGTTGCTGTTAGTGACAGGGGCCCCAGCCAGGCAGGTTTCAGGATTGGGGAGCACTTGCTTCGGCTCCCTTGCTCTCATGGGCAGCCTCTTC	ACACTCTTTCCCTACACGACGCTCTTCCGATCTNNNNTTTGGCAATGGAGGCATTGG	TGGAGTTCAGACGTGTGCTCTTCCGATCTGAAGAGGCTGCCCATGAGAG
HEK4OT3	GGCACGACGGCTGGAGGTGG	GGG	GGTCTGAGGCTCGAATCCTGGCAGCAGGTCCTTCATGGCAAGGCGGGAAAAGAGAAAAGCCAACGGGTTCTCATGCTGGGAAAAGATGCCGGGCACGACGGCTGGAGGTGGGGGGTTGGGAGTGGGTGGGATGCTTGCGTGCCCTGCATGAGGTGCAGGGATATGGAGGCCACAG	ACACTCTTTCCCTACACGACGCTCTTCCGATCTNNNNGGTCTGAGGCTCGAATCCTG	TGGAGTTCAGACGTGTGCTCTTCCGATCTCTGTGGCCTCCATATCCCTG
HEK4OT4	GGCATCACGGCTGGAGGTGG	AGG	TTCCACCAGAACTCAGCCCAGGCTGCTGTGGGATGGAATCACCTGCACCCGGATGTTCTTTCTGGGCTGGTACATACAGGCAAGGCATCACGGCTGGAGGTGGAGGGGGCCTAACCCGGGGTTGCCCAGGAAGGGGTTTGCACATGGATTCGGTGTGTTGTGGAGGAACCGAGG	ACACTCTTTCCCTACACGACGCTCTTCCGATCTNNNNTTTCCACCAGAACTCAGCCC	TGGAGTTCAGACGTGTGCTCTTCCGATCTCTCGGTTCCTCCACAACAC
HEK4OT5	GGCGCTGCGGCGGGAGGTGG	AGG	CACGGGAAGGACAGGAGAAGGTGCTGGACCGCCTGGACTTTGTGCTGACCAGCCTTGTGGCGCTGCGGCGGGAGGTGGAGGAGCTGAGAAGCAGCCTGCGAGGGCTTGCGGGGGAGATTGTTGGGGAGGTCCGGTGAGTAATGCGGCTTCTTCTCCTGCTTTATCCCTCCCCTGC	ACACTCTTTCCCTACACGACGCTCTTCCGATCTNNNNCACGGGAAGGACAGGAGAAG	TGGAGTTCAGACGTGTGCTCTTCCGATCTGCAGGGGAGGGATAAAGCAG
EMX1OT1	GAGTTAGAGCAGAAGAAGAA	AGG	TGCCCAATCATTGATGCTTTTATACCATCTTGGGGTTACAGAAAGAATAGGGGCTTATGGCATGGCAAGACAGATTGTCAGAGTTAGAGCAGAAGAAGAAAGGCATGGAGTAAAGGCAATCTTGTGCAGATGTACAGGTAGCAGCCCTCAGAAAAAATAGGTGATAGTCTATGGTAAATGTTTCT	ACACTCTTTCCCTACACGACGCTCTTCCGATCTNNNNTGCCCAATCATTGATGCTTTT	TGGAGTTCAGACGTGTGCTCTTCCGATCTAGAAACATTTACCATAGACTATCACCT
EMX1OT2	GAGTCTAAGCAGAAGAAGAA	GAG	GTAGCCTCTTTCTCAATGTGCTTCAACCCATCACGGCCTTTGCAAATAGAGCCCTTTATTCATAGTAGACAAGAGTCTAAGCAGAAGAAGAAGAGAGCCACTACCCAACCATCTACTCTTCTAATGGT	ACACTCTTTCCCTACACGACGCTCTTCCGATCTNNNNAGTAGCCTCTTTCTCAATGTGC	TGGAGTTCAGACGTGTGCTCTTCCGATCTGCTTTCA

			GTTTTCCTACAAAGGCCAAGTCATGAGACTGCATCCTTGTGAAAGC		CAAGGATGCAGTCT
EMX1OT3	GAGGCCGAGCAGAAGAAAGA	CGG	GAGCTAGACTCCGAGGGGAGGCTGCGAGCCGCAAGCGCAGGAGCCGGGTGGGAGAGAGACCCCTTCTTCTGCAAATGAGGAGGCCGAGCAGAAGAAAGACGGCGACAGATGTTGGGGGGAGGGGACGGTTTGTGAGGGATAGGGAGAGAAAGTCTAAGTGAGAGCAGGACGAGGA	ACACTCTTTCCCTACACGACGCTCTTCCGATCTNNNNGAGCTAGACTCCGAGGGGA	TGGAGTTCAGACGTGTGCTCTTCCGATCTTCCTCGTCCTGCTCTCACTT
EMX1OT4	GAGTCCTAGCAGGAGAAGAA	GAG	AGAGGCTGAAGAGGAAGACCAGACTCAGTAAAGCCTGGAGGCTGCCAGGTAGGGCTGGGGCCAGCATGACCTGAGTCCTAGCAGGAGAAGAAGAGGCAGCCTAGAGTCTTCTGTGAAGTGCACATAGAAGAGAGACTGGGGCCAAGCCACAAAAGATAGAATGCACAGCTGGGCC	ACACTCTTTCCCTACACGACGCTCTTCCGATCTNNNNAGAGGCTGAAGAGGAAGACCA	TGGAGTTCAGACGTGTGCTCTTCCGATCTGGCCCAGCTGTGCATTCTAT
EMX1OT5	AAGTCTGAGCACAAGAAGAA	TGG	GTAGTTCTGACATTCCTCCTGAGGGAAAATAAATAAATTAATTAAAAATATATATATATATGTATAATGATAAACATGCTAACAAAGTCTGAGCACAAGAAGAATGGTGAGAAGGAATACATTTTATCTAATAAATATGTAAGCCATTAATAAAATGTAAACCATTAAAACAACAAATAAACCTTTCAGATATTGACCA	ACACTCTTTCCCTACACGACGCTCTTCCGATCTNNNNGTAGTTCTGACATTCCTCCTGAG	TGGAGTTCAGACGTGTGCTCTTCCGATCTTGGTCAATATCTGAAAGGTTTATTTGT
EMX1OT6	GAGTCCGGGAAGGAGAAGAA	AGG	CCAAGAGGGCCAAGTCCTGGCTGTCTGCCTCTGACGACGAGCAAGGTGGAGGCCCTTGGTTAGCAGGATGGGTGGTGAGGAGTCCGGGAAGGAGAAGAAAGGCTCAGCGCGGCTTGCCTGAGCCTCCCTCCTCCCAGCTCCCGGCCCCTGCTGCCGGCGGCTGTCACTCCTCGCTG	ACACTCTTTCCCTACACGACGCTCTTCCGATCTNNNNCCAAGAGGGCCAAGTCCTG	TGGAGTTCAGACGTGTGCTCTTCCGATCTCAGCGAGGAGTGACAGCC
EMX1OT7	GAGCCGGAGCAGAAGAAGGA	GGG	CACTCCACCTGATCTCGGGGCGCTGTGCGCTGAGGAAGGCGCGGGCGAGCCGGAGCAGAAGAAGGAGGGAGGGAGCCAGCCGCTGCAGCCACCACCGCCACCATGTCCTACCAAGGCAAGAAGAACATCCCGCGGATCACGGTGAGTCCGGGCGCCGCTGCTCCCTCCCTCCTCG	ACACTCTTTCCCTACACGACGCTCTTCCGATCTNNNNCACTCCACCTGATCTCGGGG	TGGAGTTCAGACGTGTGCTCTTCCGATCTCGAGGAGGGAGGGAGCAG
EMX1OT8	AAGTCCGAGGAGAGGAAGAA	AGG	ACCACAAATGCCCAAGAGACATCACCACTTGGAGAGTCAGAGGTCACAAAAGAGGGGCCCAACTCCTGTAGAAGTCCGAGGAGAGGAAGAAAGGGTTCTGGAGCTCTCAGGCGTCAGGGCCAGG	ACACTCTTTCCCTACACGACGCTCTTCCGATCTNNNNACCACAAATGCCCAAGAGAC	TGGAGTTCAGACGTGTGCTCTTCCGATCTGA

			CCTGCACCCTTCTGTGCCCCTCCATGAATGGCTGGCCGGCCCTTGACTGTGTC		CACAGTCAAGGGCCGG
EMX1OT9	GAATCCAAGCAGGAGAAGAA	GGA	CCCACCTTTGAGGAGGCAAAAGGGAATAAACTTGTGCTTATTTGTTGGAAGAGCAAATATGTTTTTTTGAAACCGAATTATGGATGGGGATGTGGGGGTGGGAACTAGGCAAGGGTCTCAGGGGAATCCAAGCAGGAGAAGAAGGAGGGAAAAACCACTCTCTTCTCAGATGGAA	ACACTCTTTCCCTACACGACGCTCTTCCGATCTNNNNCCCACCTTTGAGGAGGCAAA	TGGAGTTCAGACGTGTGCTCTTCCGATCTTTCCATCTGAGAAGAGAGTGGT
EMX1OT10	ACGTCTGAGCAGAAGAAGAA	TGG	GTCATACCTTGGCCCTTCCTCTGTACTCTATACAGAGTCCAGCTCTGGCCTGGGAAAATACTTTCAGACAAAACGTCTGAGCAGAAGAAGAATGGACAGAACTCTGAGGACATTCTTGAGGCACTGGCAGAACCTCTGCAGGAAGACGAGAGCATTGCTGGTGTGGGCCTAGGGA	ACACTCTTTCCCTACACGACGCTCTTCCGATCTNNNNGTCATACCTTGGCCCTTCCT	TGGAGTTCAGACGTGTGCTCTTCCGATCTTCCCTAGGCCCACACCAG
BCL11AOT1	CCTATCACTGGCTCCAGGAA	GGG	ACCTGTGGGCATCCTGAGTTGCTTCTGATGTCCCACCCATCACCTTGACCTGCTCAGAGCAGAGCATTGTTCTGAAATCTGAGGCATTGTCCTGCCCACTGGCCTATCACTGGCTCCAGGAAGGGCCTAGTGTCTCTGACCAGCTCTAGATCACCTCCTCCTCCTCCTGAGCCCTGTACGTTGCCAGGCTGATGAGAGGAGTGGGGCCGTGA	ACACTCTTTCCCTACACGACGCTCTTCCGATCTNNNNACCTGTGGGCATCCTGAGTTGC	TGGAGTTCAGACGTGTGCTCTTCCGATCTTCACGGCCCCACTCCTCTCA
BCL11AOT2	CTTATCATAGGCCCCAGGAA	AGG	CTTGGCGCAGTTCCTGTGTATGGATATTCTTACAGAATCGCTACTCTCCCTCTCCTTTGAGCTGGCCTAGCTTTGGCTTATCATAGGCCCCAGGAAAGGCCAGGGGACTGGGGTACCGGTTAGAGGGATATAAAAGTTCATTCTGCCTTGTACGTATGTTTAATTGATTAGAACACTTCATTTTCTCACAGCATGTA	ACACTCTTTCCCTACACGACGCTCTTCCGATCTNNNNCTTGGCGCAGTTCCTGTGTATG	TGGAGTTCAGACGTGTGCTCTTCCGATCTTACATGCTGTGAGAAAATGAAGTGT
CCR5OT1	TAGGGTGTCAACTCTTGACA	CAG	GACTTCTGGTTATCAGTAGGCTGTTAGTAGTTAAGTTTTGGGGGAGTCAAAAGTTATACCTGCATTTGACTGTGTCAAGAGTTGACACCCTAACCTCCACGTTGTTTAAGAGTTAACTGTAGTTTGAACTACACTAGTTTGTACTGGTTTGCAGTTATTTATTATACTATATTCATCATTACTTTTATGTTTCAATGAACCATTGTTGAGCCATGATCCATTTTG	ACACTCTTTCCCTACACGACGCTCTTCCGATCTNNNNGACTTCTGGTTATCAGTAGGCTGTT	TGGAGTTCAGACGTGTGCTCTTCCGATCTCAAAATGGATCATGGCTCAA
CCR5OT3	TTATCTGTCAACTCTTGACA	AAT	TGAGGACTTGGTGAATCGTGGTATCATTTGTCAAGAGTTGACAGATAAGAGGAAGGGAGTAGGTTTGTCAGGGAAAGGGTTAATTTTTTTT	ACACTCTTTCCCTACACGACGCTCTTCCGATCTNNNNTGAGGA	TGGAGTTCAGACGTGTGCTCTTCCG

			TTTTGGAGACTTGTTGCC CAGGCTGGAGTACAATGG CATGATCTTGGCTCACCA CAACTTCCGCCTCCTGGG TTCAAGTGATTTTGCTGC CTCAGCCTCCCGAGTAGC TGGGATTACAGGCATGTG CCACCACACCCAGCTAAT T	CTTGGTGAA TCGTGG	ATCTAA TTAGC TGGGT GTGGT GGC
CCR5OT8	ATACCTGCCAACTCTTGACA	TGG	CAATTACAGAAAGTGGAT TGGTTGTGGAATGTACAG ATTAACAATGGAGAGCAC ATAACCCTCAGTCTGGCA CACCAGTGGAGGCTAAAG AAACTGAAAGGTGTTCTG CCCAGGGTGCAGGGACT CCATGTCAAGAGTTGGCA GGTATGGAAAGCAAGAG GGTTGGGACAGAAACCA GGGTAATTAATTAAGTGA CTATTTTTAGGACTAACTT CAAGGGTTACATGAGCTC CCTCCCCTCCAAAAACAA ATCATTAGGAAAATCTCA	ACACTCTTT CCCTACAC GACGCTCTT CCGATCTNN NNCAATTAC AGAAAGTG GATTGGTTG	TGGAG TTCAG ACGTG TGCTC TTCCG ATCTTT GTCTT GAGAT TTTCCT AATGA TTT
CXCR4OT8	TGTTCTCAGCACATCATGGT	TGT	TTGCTGAAAGTGACGACA CAGCCAGTGAAGCTGGG ACTGGAATGTCAGTTTCC ATAACTCCAGCCCCCTTT TTTAACCGTTAGGAAACA TCCTTTCACCTCAGGTCC CCTCAGTCTCTAGTCCAC ACCATGATGTGCTGAGAA CAGGAGGCATACTATCAG CATTTTAAAAGGACCTCTT CCTTGTTTCTCCTGTTCTC TGGCCCCAGAGAGCTCTT CTGAGACCCTGGGGTCC AGAAGGGAGCCCTGGAG AACTTAGCTCCAAAGACT TTTCCTGAGCA	ACACTCTTT CCCTACAC GACGCTCTT CCGATCTNN NNTTGCTGA AAGTGACG ACACAG	TGGAG TTCAG ACGTG TGCTC TTCCG ATCTT GCTCA GGAAA AGTCT TTGGA

SaCas9 orthogonal R-loop sites:

Site Name	Protospacer	PAM	Amplicon	HTS-F	HTS-R
SaR1	GTGGTAGACAGCATGTGTCCTA	AAGGGT	TGGTGGAGTGCTCTGTGTTTGTCTTTATAAACCCAGATGAGAGGATGAAGGCAACAAGCTTCTGTACCAACATACATGCCCCTTTGCCTCAAGTCTGGTTATTTTAGGGGGATGCTAGGTTGCTTTGGGTCTACCTTACTGAGAAAATGGCCCCAGGTCATTGTCATGTCCAGTTGTGGTAGACAGCATGTGTCCTAAAGGGTATATTCACATGCATGTGCAAAAATACAGGGGTCCTTCTAACCCTATCACAGAGAAGCAGGAGACTGC	ACACTCTTTCCCTACACGACGCTCTTCCGATCTNNNNTGCAGTCTCCTGCTTCTCTG	TGGAGTTCAGACGTGTGCTCTTCCGATTGGTGGAGTGCTCTGTGTTTG
SaR2	ATTTACAGCCTGGCCTTTGGGG	TCGGGT	GCTACAGAAAGGTCAGCAGCTATATTTAACCTCAGACCAGGGTGCGGTGGGAGATCTGGTTTCCGGAAGACGGAATGGGGAGAAGGGCAGGTTCCCCGAGGCGCCCAGACACCCAATCCTCCCGGTGACATTTACAGCCTGGCCTTTGGGGTCGGGTCAACGCTAGGCTGGCAGGGGAAGGGCGGGGCCGTGAGGTGAGCCGGCGCTGCAGGAAGGGGCCACCACCAGAGGGGCCATTTTGCGGTGGAAATGTCC	ACACTCTTTCCCTACACGACGCTCTTCCGATCTNNNNGGACATTTCCACCGCAAAATG	TGGAGTTCAGACGTGTGCTCTTCCGATGCTACAGAAAGGTCAGCAGC
SaR3	GTGTCAGGTAATGTGCTAAACA	GAGAGT	AAGTGTTCAGCTGCTTTTCTTTCATTTATTCCACATATAATTACTATAATTGCTAAACATTTATTTAGTGTCAGGTAATGTGCTAAACAGAGAGTTACTGCTCAGACATGTAATAATAATAAATAACACATCAAATAACCATACCATTTTAAGCTGTAGTATTATGAAGGGAAATCTGGAGCAAAGAGAATAGACTGTAGGGAAACCAGTTAAGAAATAGGACATGGAGGCTAGGTGCAG	ACACTCTTTCCCTACACGACGCTCTTCCGATCTNNNNCTGCACCTAGCCTCCATGTC	TGGAGTTCAGACGTGTGCTCTTCCGATCTGCTGTGGCATCCAGAGACAT
SaR4	GGTGGAGGAGGGTGCATGGGGT	CAGAAT	TTTGCTTATCCAGAAAAGGGAGTGATTGCTTCCAGGGGCCTCAGGGGAATAAATCATAGAATCCTGGACAAGGTTTGAAGGACAGGTAGGATTTGGGTGGGTGGAGGAGGGTGCATGGGGTCAGAATTGTAACCGAAAACTCATTCCAGGTGGATAGAGAAAATTTCTAGTGTTGTTGTTTTTAAACTATTTGGGGGACTGGCACAGACCCTTTTTGAATACCTGATGGGCTCACATTTCTGTCGAATCCCAG	ACACTCTTTCCCTACACGACGCTCTTCCGATCTNNNNGGAGGTGGAGAGAGGATGT	TGGAGTTCAGACGTGTGCTCTTCCGATCTTCCTGAGGTCTAGGAACCCG
SaR5	TCTGCTTCTCCAGCCCTGGC	CTGGGT	ATGTGGGCTGCCTAGAAAGGCATGGATGAGAGAAGCCTGGAGACAGGGATCCCAGGGAAACGCCCATGCAATTAGTCTATTTCTGCTGCAAGTAAGCATGCATTTGTAGGCTTGATGCTTTTTTTCTGCTTCTCCAGCCCTGGCCTGGGTCAATCCTTGGGGCCCAGACTGAGCACGTGATGGCAGAGGAAAGGAAGCCCTGCTTCCTCCAGAGGGCGTCGCAGGACAGCTTTTCCTAGACAGGGGCTAGTATGTGCAGCTCCTGCACCGGGATACTGGTTGACAAGTTTGGCTGGG	ACACTCTTTCCCTACACGACGCTCTTCCGATCTNNNNATGTGGGCTGCCTAGAAAGG	TGGAGTTCAGACGTGTGCTCTTCCGATCTCCCAGCCAAACTTGTCAACC
SaR6	GATGTTCCAATCAGTACGCA	GAGAGT	CATTGCAGAGAGGCGTATCATTTCGCGGATGTTCCAATCAGTACGCAGAGAGTCGCCGTCTCCAAGGTGAAAGCGGAAGTAGGGCCTTCGCGCACCTCATGGAATCCCTTCTGCAGCACCTGGATCGCTTTTCCGAGCTTCTGGCGGTCTCAAGCACTACCTACGTCAGCACCTGGGACCCC	ACACTCTTTCCCTACACGACGCTCTTCCGATCTNNNNCATTGCAGAGAGGCGTATCA	TGGAGTTCAGACGTGTGCTCTTCCGATCTGGGTCCCAGGTGCTGAC

Supplementary Table 5. Primers for generating base editor amplicons for IVT.

Name	Sequence (5′–3′)
IVT-F	TCGAGCTCGGTACCTAATACGACTCACTATAAGG
IVT-R	TTTTTTTTTTTTTTTTTTTTTTTTTTTTTTTTTTTTTTTTTTTTTTTTTTTTTTTTTTTTTTTTTTTTTTTTTT<br>TTTTTTTTTTTTTTTTTTTTTTTTTTTTTTTTTTTTTTTTTTTTTTCTTCCTACTCAGGCTTTATTCAAA<br>GACCA

Supplementary Table 6. Chemically synthesized guide RNAs used for T cell and HSC experiments.

Site/Guide ID*	Protospacer sequence (5'–3')	sgRNA scaffold (5'–3')
CCR5	CAATGTGTCAACTCTTGACA	SpCas9 sgRNA EZ Kit scaffold from Synthego (with 2'-O-Methyl and 3' phosphorothioate bond modifications)
CXCR4	CAGTTTCAGCACATCATGGT	SpCas9 sgRNA EZ Kit scaffold from Synthego (with 2'-O-Methyl and 3' phosphorothioate bond modifications)
BCL11A	TTTATCACAGGCTCCAGGAA	SpCas9 sgRNA EZ Kit scaffold from Synthego (with 2'-O-Methyl and 3' phosphorothioate bond modifications)

*See Supplementary Table 4 for the list of target sites

Supplementary Table 7. cDNA amplicon sequences and primers for RNA off-target analysis.

Name	Amplicon	HTS-F	HTS-R
RSL1D1	TTGGCTTTCCAAATCAGTGGGTCTGACTTGAGGTCTGTGATGTGACCCTTTTCCTCACCTGCTCAACCATTATTCACATGGACTCCATCATATTCATTTGTAGTCATTCCCAGAGTGGCCCAGTGAGGGTCTCGCTGTATGAGAGTCGGCTACGGAATTTAGGAGAAACAGAAGTTTCTTGGCTTTCATGCTGAGCTTGTTGGTCTAAGCTTATGAG	ACACTCTTTCCCTACACGACGCTCTTCCGATCTNNNNTGGCTTTCCAAATCAGTGGGTC	TGGAGTTCAGACGTGTGCTCTTCCGATCTCTCATAAGCTTAGACCAACAAGC
CTNNB1	TTTGATGGAGTTGGACATGGCCATGGAACCAGACAGAAAAGCGGCTGTTAGTCACTGGCAGCAACAGTCTTACCTGGACTCTGGAATCCATTCTGGTGCCACTACCACAGCTCCTTCTCTGAGTGGTAAAGGCAATCCTGAGGAAGAGGATGTGGATACCTCCCAAGTCCTGTATGAGTGGGAACAGGGATTTTCTCAGTCCTTCACTCAAGAACAAGTAGCTGG	ACACTCTTTCCCTACACGACGCTCTTCCGATCTNNNNATTTGATGGAGTTGGACATGGCC	TGGAGTTCAGACGTGTGCTCTCCAGCTACTTGTTCTTGAGTGAAGG
IP90	CTGGTTGACCAATCTGTGGTGAATAGTGGAAATCTGCTCAATGACATGACTCCTCCTGTAAATCCTTCACGTGAAATTGAGGACCCAGAAGACCGGAAGCCCGAGGATTGGGATGAAAGACCAAAAATCCCAGATCCAGAAGCTGTCAAGCCAGATGACTGGGATGAAGATGCCCCTGCTAAGATTCCAGATGAAGAGGCCACAAAACCCGAAGGCTGGTTAGATGATGAGCCTGAGTACGTAC	ACACTCTTTCCCTACACGACGCTCTTCCGATCTNNNNCTGGTTGACCAATCTGTGGTG	TGGAGTTCAGACGTGTGCTCTCTGCGTCTGGATCAGGTACG

Supplementary Table 8. Primers for PCR1 and PCR2 in mESc 12kChar library analysis of base editors. Samples for the library analysis (11 samples with two biological replicates for 22 samples total) were amplified and barcoded for analysis. We performed 4 NextSeq runs with up to 7 samples on each. Primers were assigned to be unique among samples on each run. Run 1 contained 7 samples, barcoded with FWD PCR1A and REV PCR2-1 through PCR2-7. Run 2 contained 7 samples, barcoded with FWD PCR1B and REV PCR2-1 through PCR2-7. Run 3 contained 7 samples, barcoded with FWD PCR1C and REV PCR2-1 through PCR2-7. Run 4 contained 1 sample, barcoded with FWD PCR1D and REV PCR2-1.

Name	Purpose	Sequence
Fwd PCR1A	Library Fwd PCR1	AATGATACGGCGACCACCGAGATCTACACATTACTCGACACTCTTTCCCTACACGAC
Fwd PCR1B	Library Fwd PCR1	AATGATACGGCGACCACCGAGATCTACACTCCGGAGAACACTCTTTCCCTACACGAC
Fwd PCR1C	Library Fwd PCR1	AATGATACGGCGACCACCGAGATCTACACCGCTCATTACACTCTTTCCCTACACGAC
Fwd PCR1D	Library Fwd PCR1	AATGATACGGCGACCACCGAGATCTACACGAGATTCCACACTCTTTCCCTACACGAC
Rev PCR1 Lib	Library Rev PCR1	GTGACTGGAGTTCAGACGTGTGCTCTTC CGATCTGTGGAAAGGACGAAACACCG
Fwd PCR2 Lib	Library Fwd PCR2	AATGATACGGCGACCACCGAGATCTACAC
Rev PCR2-1	Library Rev PCR2	CAAGCAGAAGACGGCATACGAGATATCGTGATGTGACTGGAGTTCAGACGTGTGCT
Rev PCR2-2	Library Rev PCR2	CAAGCAGAAGACGGCATACGAGATATACATCGGTGACTGGAGTTCAGACGTGTGCT
Rev PCR2-3	Library Rev PCR2	CAAGCAGAAGACGGCATACGAGATATGCCTAAGTGACTGGAGTTCAGACGTGTGCT
Rev PCR2-4	Library Rev PCR2	CAAGCAGAAGACGGCATACGAGATATTGGTCAGTGACTGGAGTTCAGACGTGTGCT
Rev PCR2-5	Library Rev PCR2	CAAGCAGAAGACGGCATACGAGATATCACTGTGTGACTGGAGTTCAGACGTGTGCT
Rev PCR2-6	Library Rev PCR2	CAAGCAGAAGACGGCATACGAGATATATTGGCGTGACTGGAGTTCAGACGTGTGCT
Rev PCR2-7	Library Rev PCR2	CAAGCAGAAGACGGCATACGAGATATGATCTGGTGACTGGAGTTCAGACGTGTGCT

Supplementary Note 1. Evolved TadA-CD amino acid sequences.

TadA-CDa:

MSEVEFSHEYWMRHALTLAKRARDEGAGPVGAVLVLNNRVIGEGWNRAIGLHDPTAHAEIM
ALRQGGLVMQNYRLFDATLYVTFEPCVMCAGAMINSRIGRVVFGVRNSKRGAAGSLMNVLN
YPGMNHRVEITEGILADECAALLCDFYRMPRQVFNSQKKAQSSIN

TadA-CDb:

MSEVEFSHEYWMRHALTLAKRARDEGAGPVGAVLVLNNRVIGEGWNRAIGLHDPTAHAEIM
ALRQGGLVMQNYRLFDATLYVTFEPCVMCAGAMINSRIGRVVFGVRNSKRGAAGSLMNVLN
YPGMNHRVEITEGILADECAALLCDFYRMPRRVFNSQKKAQSSIN

TadA-CDc:

MSEVEFSHEYWMRHALTLAKRARDEGAGPVGAVLVLNNRVIGEGWNRAIGLHDPTAHAEIM
ALRQGGLVMQNYRLFDATLYVTFEPCVMCAGAMINSRIGRVVFGVRNSKRGAAGSLMNVLN
YPGMNHRVEITEGILADECAALLCDFYRIPRQVFNSQKKAQSSIN

TadA-CDd:

MSEVEFSHEYWMRHALTLAKRARDERKAPVGAVLVLNNRVIGEGWNRAIGLHDPTAHAEIIA
LRQGGLVMQNYRLIDATLYVTFEPCVMCAGAMINSRIGRVVFGVRNSKRGAAGSLMNVLNY
PGMNHRVEITEGILADECAALLCDFYRMPRQVFNAQKKAQSSIN

TadA-CDe:

MSEVEFSHEYWMRHALTLAKRARDERAGPVGAVLVLNNRVIGEGWNRAIGLHDPTAHAEIM
ALRQGGLVMQNHRLIDATLYVTFEPCVMCAGAMINSRIGRVVFGVRNSKRGAAGSLMNVLN
YPGMNHRVEITEGILADECAALLCDFYRMPRHVFNSQKKAQSSIN

TadA-Dual:

MSEVEFSHEYWMRHALTLAKRARDEGEAPVGAVLVLNNRVIGEGWNRRIGLHDPTAHAEIM
ALRQGGLVMQNSRLIDATLYVTFEPCVMCAGAMINSRIGRVVFGVRNSKRGAAGSLMNVLN
YPGMNHRVEITEGILADECAALLCDFYRMPRQVFNAQKKAQSSIN

Supplementary Note 2. Sequence of the Cas9 components of the base editors in this study.

SpCas9 D10A:

DKKYSIGLAIGTNSVGWAVITDEYKVPSKKFKVLGNTDRHSIKKNLIGALLFDSGETAEATRLK
RTARRRYTRRKNRICYLQEIFSNEMAKVDDSFFHRLEESFLVEEDKKHERHPIFGNIVDEVAY
HEKYPTIYHLRKKLVDSTDKADLRLIYLALAHMIKFRGHFLIEGDLNPDNSDVDKLFIQLVQTY
NQLFEENPINASGVDAKAILSARLSKSRRLENLIAQLPGEKKNGLFGNLIALSLGLTPNFKSNF
DLAEDAKLQLSKDTYDDDLDNLLAQIGDQYADLFLAAKNLSDAILLSDILRVNTEITKAPLSAS
MIKRYDEHHQDLTLLKALVRQQLPEKYKEIFFDQSKNGYAGYIDGGASQEEFYKFIKPILEKM
DGTEELLVKLNREDLLRKQRTFDNGSIPHQIHLGELHAILRRQEDFYPFLKDNREKIEKILTFRI
PYYVGPLARGNSRFAWMTRKSEETITPWNFEEVVDKGASAQSFIERMTNFDKNLPNEKVLP
KHSLLYEYFTVYNELTKVKYVTEGMRKPAFLSGEQKKAIVDLLFKTNRKVTVKQLKEDYFKKI
ECFDSVEISGVEDRFNASLGTYHDLLKIIKDKDFLDNEENEDILEDIVLTLTLFEDREMIEERLK

TYAHLFDDKVMKQLKRRRYTGWGRLSRKLINGIRDKQSGKTILDFLKSDGFANRNFMQLIHD
DSLTFKEDIQKAQVSGQGDSLHEHIANLAGSPAIKKGILQTVKVVDELVKVMGRHKPENIVIE
MARENQTTQKGQKNSRERMKRIEEGIKELGSQILKEHPVENTQLQNEKLYLYYLQNGRDMY
VDQELDINRLSDYDVDHIVPQSFLKDDSIDNKVLTRSDKNRGKSDNVPSEEVVKKMKNYWR
QLLNAKLITQRKFDNLTKAERGGLSELDKAGFIKRQLVETRQITKHVAQILDSRMNTKYDEND
KLIREVKVITLKSKLVSDFRKDFQFYKVREINNYHHAHDAYLNAVVGTALIKKYPKLESEFVYG
DYKVYDVRKMIAKSEQEIGKATAKYFFYSNIMNFFKTEITLANGEIRKRPLIETNGETGEIVWD
KGRDFATVRKVLSMPQVNIVKKTEVQTGGFSKESILPKRNSDKLIARKKDWDPKKYGGFDSP
TVAYSVLVVAKVEKGKSKKLKSVKELLGITIMERSSFEKNPIDFLEAKGYKEVKKDLIIKLPKYS
LFELENGRKRMLASAGELQKGNELALPSKYVNFLYLASHYEKLKGSPEDNEQKQLFVEQHK
HYLDEIIEQISEFSKRVILADANLDKVLSAYNKHRDKPIREQAENIIHLFTLTNLGAPAAFKYFDT
TIDRKRYTSTKEVLDATLIHQSITGLYETRIDLSQLGGD

eNme2-C Cas9 (S6P mutation relative to reported eNme2-C sequence):

AAFKPNPINYILGLAIGIASVGWAMVEIDEEENPIRLIDLGVRVFERAEVPKTGDSLAMARRLA
RSVRRLTRRRAHRLLRARRLLKREGVLQAADFDENGLIKSLPNTPWQLRAAALDRKLTPLE
WSAVLLHLIKHRGYLSQRKNEGETADKELGALLKGVANNAHALQTGDFRTPAELALNKFEKE
SGHIRNQRGDYSHTFSRKDLQAELILLFEKQKEFGNPHVSGGLKEGIETLLMTQRPALSGDA
VQKMLGHCTFEPAEPKAAKNTYTAERFIWLTKLNNLRILEQGSERPLTDTERATLMDEPYRK
SKLTYAQARKLLGLEDTAFFKGLRYGKDNAEASTLMEMKAYHAISRALEKEGLKDKKSPLNL
SSELQDEIGTAFSLFKTDEDITGRLKDRVQPEILEALLKHISFDKFVQISLKALRRIVPLMEQGK
RYDEACAEIYGDHYGKKNTEEKIYLPPIPADEIRNPVVLRALSQARKVINGVVRRYGSPARIHI
ETAREVGKSFKDRKEIEKRQEENRKDREKAAAKFREYFPNFVGEPKSKDILKLRLYEQQHGK
CLYSGKEINLVRLNEKGYVEIDAALPFSRTWDDSFNNKVLVLGSENQNKGNQTPYEYFNGK
DNSREWQEFKARVETSRFPRSKKQRILLQKFDEDGFKECNLNDTRYVNRFLCQFVADHILLT
GKGKRRVFASNGQITNLLRGFWGLRKVRAENDRHHALDAVVVACSTVAMQQKITRFVRYKE
MNAFDGKTIDKETGKVLHQKTHFPQPWEFFAQEVMIRVFGKPDGKPEFEEADTPEKLRTLLA
EKLSSRPEAVHEYVTPLFVSRAPNRKMSGAHKDTLRSAKRFVKHNEKISVKRVWLTEIKLAD
LENMVNYKNGREIELYEALKARLEAYGGNAKQAFDPKDNPFYKKGGQLVKAVRVEKTQESG
VLLNKKNAYTIADNGDMVRVDVFCKVDKKGKNQYFIVPIYAWQVAENILPDIDCKGYRIDDSY
TFCFSLHKYDLIAFQKDEKSKVEFAYYINCDSSNGRFYLAWHDKGSKEQQFRISTQNLVLIQK
YQVNELGKEIRPCRLKKRPPV

SaCas9 D10A:

GKRNYILGLAIGITSVGYGIIDYETRDVIDAGVRLFKEANVENNEGRRSKRGARRLKRRRRHRI
QRVKKLLFDYNLLTDHSELSGINPYEARVKGLSQKLSEEEFSAALLHLAKRRGVHNVNEVEE
DTGNELSTKEQISRNSKALEEKYVAELQLERLKKDGEVRGSINRFKTSDYVKEAKQLLKVQK
AYHQLDQSFIDTYIDLLETRRTYYEGPGEGSPFGWKDIKEWYEMLMGHCTYFPEELRSVKY
AYNADLYNALNDLNNLVITRDENEKLEYYEKFQIIENVFKQKKKPTLKQIAKEILVNEEDIKGYR
VTSTGKPEFTNLKVYHDIKDITARKEIIENAELLDQIAKILTIYQSSEDIQEELTNLNSELTQEEIE
QISNLKGYTGTHNLSLKAINLILDELWHTNDNQIAIFNRLKLVPKKVDLSQQKEIPTTLVDDFILS
PVVKRSFIQSIKVINAIIKKYGLPNDIIIELAREKNSKDAQKMINEMQKRNRQTNERIEEIIRTTGK
ENAKYLIEKIKLHDMQEGKCLYSLEAIPLEDLLNNPFNYEVDHIIPRSVSFDNSFNNKVLVKQE
ENSKKGNRTPFQYLSSSDSKISYETFKKHILNLAKGKGRISKTKKEYLLEERDINRFSVQKDFI
NRNLVDTRYATRGLMNLLRSYFRVNNLDVKVKSINGGFTSFLRRKWKFKKERNKGYKHHAE
DALIIANADFIFKEWKKLDKAKKVMENQMFEEKQAESMPEIETEQEYKEIFITPHQIKHIKDFKD
YKYSHRVDKKPNRELINDTLYSTRKDDKGNTLIVNNLNGLYDKDNDKLKKLINKSPEKLLMYH

HDPQTYQKLKLIMEQYGDEKNPLYKYYEETGNYLTKYSKKDNGPVIKKIKYYGNKLNAHLDIT
DDYPNSRNKVVKLSLKPYRFDVYLDNGVYKFVTVKNLDVIKKENYYEVNSKCYEEAKKLKKI
SNQAEFIASFYNNDLIKINGELYRVIGVNNDLLNRIEVNMIDITYREYLENMNDKRPPRIIKTIAS
KTQSIKKYSTDILGNLYEVKSKKHPQIIKKG

Supplementary References

1. Lapinaite, A. *et al.* DNA capture by a CRISPR-Cas9-guided adenine base editor. *Science* **369**, 566–571 (2020).
2. Pettersen, E. F. *et al.* UCSF Chimera--a visualization system for exploratory research and analysis. *J Comput Chem* **25**, 1605–1612 (2004).
3. Jumper, J. *et al.* Highly accurate protein structure prediction with AlphaFold. *Nature* **596**, 583–589 (2021).
4. Rees, H. A., Wilson, C., Doman, J. L. & Liu, D. R. Analysis and minimization of cellular RNA editing by DNA adenine base editors. *Sci Adv* **5**, eaax5717 (2019).
5. Richter, M. F. *et al.* Phage-assisted evolution of an adenine base editor with improved Cas domain compatibility and activity. *Nat Biotechnol* **38**, 883–891 (2020).
6. Gaudelli, N. M. *et al.* Directed evolution of adenine base editors with increased activity and therapeutic application. *Nat Biotechnol* **38**, 892–900 (2020).
7. Doman, J. L., Raguram, A., Newby, G. A. & Liu, D. R. Evaluation and minimization of Cas9-independent off-target DNA editing by cytosine base editors. *Nat Biotechnol* **38**, 620–628 (2020).
