# Supplementary material for: Evolution of an adenine base editor into a small, efficient cytosine base editor with low off-target activity
Source: Nat Biotechnol. 2022 Nov 10;41(5):673–85. doi: 10.1038/s41587-022-01533-6 (PMC10188366; doi:10.1038/s41587-022-01533-6)
Supplement: Supplementary file 2 — Reporting Summary [file 41587_2022_1533_MOESM2_ESM.pdf]

nature portfolio

Corresponding author(s): David R. Liu

Last updated by author(s): 09/26/2022

Reporting Summary

Nature Portfolio wishes to improve the reproducibility of the work that we publish. This form provides structure for consistency and transparency in reporting. For further information on Nature Portfolio policies, see our Editorial Policies and the Editorial Policy Checklist.

Statistics

For all statistical analyses, confirm that the following items are present in the figure legend, table legend, main text, or Methods section.

n/a	Confirmed	
☐	☒	The exact sample size (*n*) for each experimental group/condition, given as a discrete number and unit of measurement
☐	☒	A statement on whether measurements were taken from distinct samples or whether the same sample was measured repeatedly
☐	☒	The statistical test(s) used AND whether they are one- or two-sided<br>*Only common tests should be described solely by name; describe more complex techniques in the Methods section.*
☒	☐	A description of all covariates tested
☐	☒	A description of any assumptions or corrections, such as tests of normality and adjustment for multiple comparisons
☐	☒	A full description of the statistical parameters including central tendency (e.g. means) or other basic estimates (e.g. regression coefficient) AND variation (e.g. standard deviation) or associated estimates of uncertainty (e.g. confidence intervals)
☒	☐	For null hypothesis testing, the test statistic (e.g. *F*, *t*, *r*) with confidence intervals, effect sizes, degrees of freedom and *P* value noted<br>*Give P values as exact values whenever suitable.*
☒	☐	For Bayesian analysis, information on the choice of priors and Markov chain Monte Carlo settings
☒	☐	For hierarchical and complex designs, identification of the appropriate level for tests and full reporting of outcomes
☐	☒	Estimates of effect sizes (e.g. Cohen's *d*, Pearson's *r*), indicating how they were calculated

Our web collection on statistics for biologists contains articles on many of the points above.

Software and code

Policy information about availability of computer code

Data collection	High-throughput sequencing data was collected on Illumina Miseq Control software (3.1) and instruments. CRISPResso files were imported into Microsoft Excel (version 16.64) for analyses.
Data analysis	High-throughput sequencing data was analyzed using CRISPresso2 (v2.0.34). Sequencing reads were demultiplexed using the MiSeq Reporter (3.1, Illumina) and fastq files were analyzed using Crispresso2. Microsoft Excel (version 16.64) was used for analyses. Prism 9 (v9.4.1,GraphPad) was used to generate dot plots and bar plots of these data. Mutato is available as a docker image at https://hub.docker.com/r/araguram/mutato. Further details and references are provided in the Methods. Library data processing and analysis were performed with Python 3.9. Library analysis code can be accessed on Github at https://github.com/alvin-hsu/BELib_Processing as indicated in the Code Availability Statement. Library samples were demultiplexed for each editor/replicate with bcl2fastq2 (v2.20, Illumina).

For manuscripts utilizing custom algorithms or software that are central to the research but not yet described in published literature, software must be made available to editors and reviewers. We strongly encourage code deposition in a community repository (e.g. GitHub). See the Nature Portfolio guidelines for submitting code & software for further information.

Data

Policy information about availability of data

All manuscripts must include a data availability statement. This statement should provide the following information, where applicable:

- Accession codes, unique identifiers, or web links for publicly available datasets
- A description of any restrictions on data availability
- For clinical datasets or third party data, please ensure that the statement adheres to our policy

High-throughput DNA sequencing FASTQ files for base editing and indel quantification are available from the NCBI SRA under BioProject PRJNA848090 (http://www.ncbi.nlm.nih.gov/bioproject/848090,). Amino acid sequences of deaminases in this study are provided in the Supplementary Information as Supplementary Note Sequences 1 and 2. CSV files containing processed data for library experiments have been uploaded to FigShare and assigned DOI 10.6084/m9.figshare.21210845. Processed data from Figures 1–6 are included as Source Data. The published structure of ABE8e (PDB ID 6VPC) can be accessed here:https://www.rcsb.org/structure/6vpc . Other data, including phage titers from evolution and mammalian cell data analysis (PRISM and Excel files) are available from the corresponding authors upon request. Plasmids encoding TadCBEs are available at Addgene.

Human research participants

Policy information about studies involving human research participants and Sex and Gender in Research.

Reporting on sex and gender	NA- samples for T cell isolation (Memorial Blood Center) and HSPCs (Fred Hutchinson Cancer Center) were purchased as de-identified samples and are IRB exempt.
Population characteristics	NA- samples for T cell isolation and HSPCs were purchased as de-identified samples and are IRB exempt.
Recruitment	NA- samples for T cell isolation and HSPCs were purchased as de-identified samples and are IRB exempt.
Ethics oversight	NA- samples for T cell isolation and HSPCs were purchased as de-identified samples and are IRB exempt.

Note that full information on the approval of the study protocol must also be provided in the manuscript.

Field-specific reporting

Please select the one below that is the best fit for your research. If you are not sure, read the appropriate sections before making your selection.

☒ Life sciences ☐ Behavioural & social sciences ☐ Ecological, evolutionary & environmental sciences

For a reference copy of the document with all sections, see nature.com/documents/nr-reporting-summary-flat.pdf

Life sciences study design

All studies must disclose on these points even when the disclosure is negative.

Sample size	Sample sizes were n = 3 or n = 4 independent biological replicates, which we and others have found to be sufficient in mammalian cell gene editing experiments to yield reproducible mean values (e.g. Chen, Hussman, et al. Cell 2021). For Figure 4d, one replicate for the YE1 samples gave low read count, likely due to RNA degradation. Therefore, the full set of RNA editing experiments was repeated a fourth time, leading to n=4 replicates for all samples except YE1 (n=3). For the library experiment, sequencing depth was chosen to obtain a minimum average of 2800 reads per designed library oligos based on our previously published work optimizing coverage of members (Arbab, Shen, et al., Cell 2020).
Data exclusions	For analysis of individual amplicons, no data were excluded. Some data have 4 replicates rather than three in cases when low read count warranted a repeat of transfections for the full set (based on low read count until sequencing). When analyzing library data, library members with fewer than 1000 reads total (across both replicates) were excluded from some analyses as indicated in the text. Furthermore, mutations in sequencing data that were likely to arise from sequencing error or batch effects (as described in the Methods) were "corrected" to their unmutated counterparts.
Replication	For individual target amplicons experiments, biological triplicate experiments were done with distinct aliquots of cells at intervals ranging from days to weeks between experiments. For Figure 4d, one replicate for the YE1 samples gave low read count, likely due to RNA degradation. Therefore, the full set of RNA editing experiments was repeated a fourth time, leading to n=4 replicates for all samples except YE1 (n=3). All other attempts at replication were successful. Findings have been replicated and the base editors developed in this work have been independently tested by several researchers in the lab. For library experiments, two biological replicates were performed and were shown to be consistent in editing outcomes (Correlation plots in Supplementary Fig. 27 of this manuscript show consistency; methods developed in Arbab, Shen, et al., Cell 2020).
Randomization	All independent biological replicates were treated identically. Thus randomization was not relevant to this study. We minimize impact of cell-to-cell variability by ensuring a minimum cell population diversity of 2,000 cells per designed library oligonucleotide and an average of 2,800 reads per designed library oligonucleotide.

Blinding | Mammalian cells used in this study were treated under identical conditions; blinding was not used.

Reporting for specific materials, systems and methods

We require information from authors about some types of materials, experimental systems and methods used in many studies. Here, indicate whether each material, system or method listed is relevant to your study. If you are not sure if a list item applies to your research, read the appropriate section before selecting a response.

Materials & experimental systems

n/a	Involved in the study
☒	☐ Antibodies
☐	☒ Eukaryotic cell lines
☒	☐ Palaeontology and archaeology
☒	☐ Animals and other organisms
☒	☐ Clinical data
☒	☐ Dual use research of concern

Methods

n/a	Involved in the study
☒	☐ ChIP-seq
☒	☐ Flow cytometry
☒	☐ MRI-based neuroimaging

Eukaryotic cell lines

Policy information about cell lines and Sex and Gender in Research

Cell line source(s)	HEK293T (ATCC CRL-3216). De-identified Human T cells were obtained from Memorial Blood Centers (St. Paul, MN). De-identified CD34+ cells were procured from the Core Center for Excellence in Hematology at the Fred Hutchinson Cancer Research Center. mESC lines containing the 10,683 library members were previously reported (Arbab and Shen Cell 2020).
Authentication	Cells were authenticated by the supplier using STR analysis.
Mycoplasma contamination	HEK293T cell lines and mESCs tested negative for mycoplasma.
Commonly misidentified lines (See ICLAC register)	None used.
